# Supplementary figures and images for: Computational analysis of functional SNPs in Alzheimer’s disease-associated endocytosis genes
Source: PeerJ. 2019 Sep 30;7:e7667. doi: 10.7717/peerj.7667 (PMC6776068; doi:10.7717/peerj.7667)

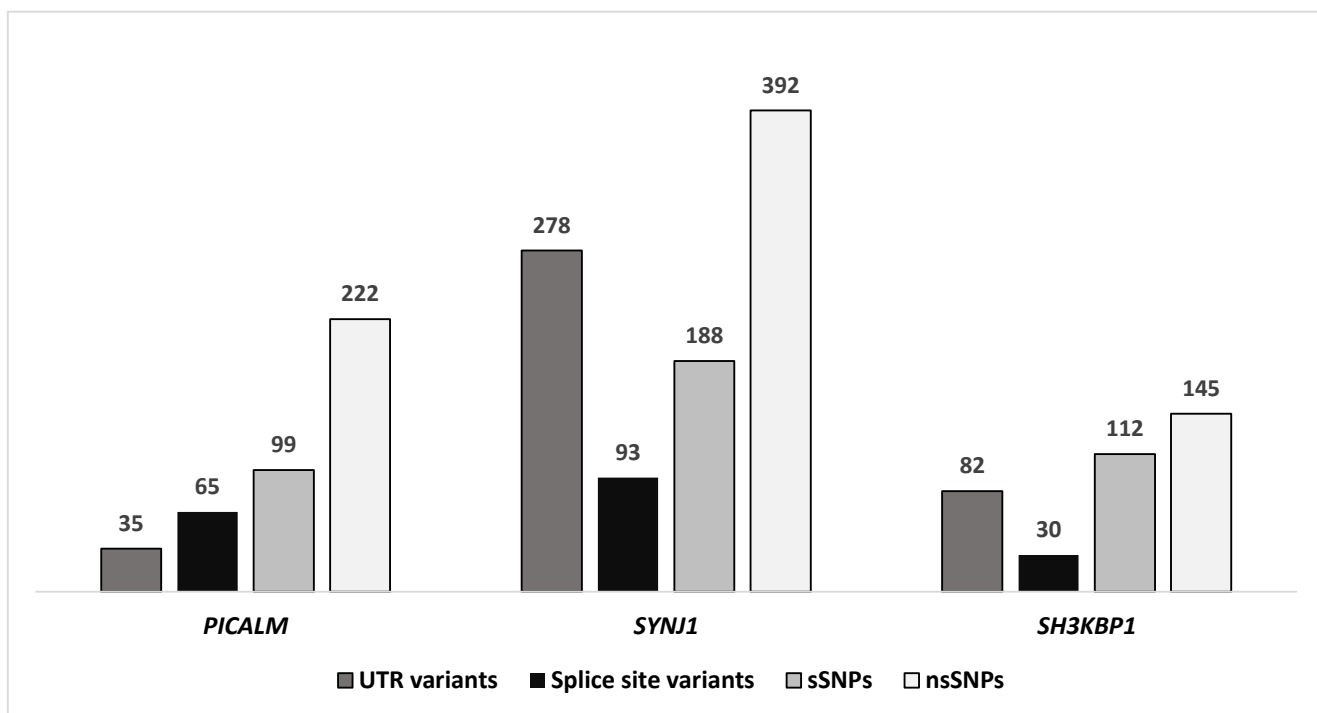

Supplement: Figure S1 [file peerj-07-7667-s001.pdf]

# ConSurf Results

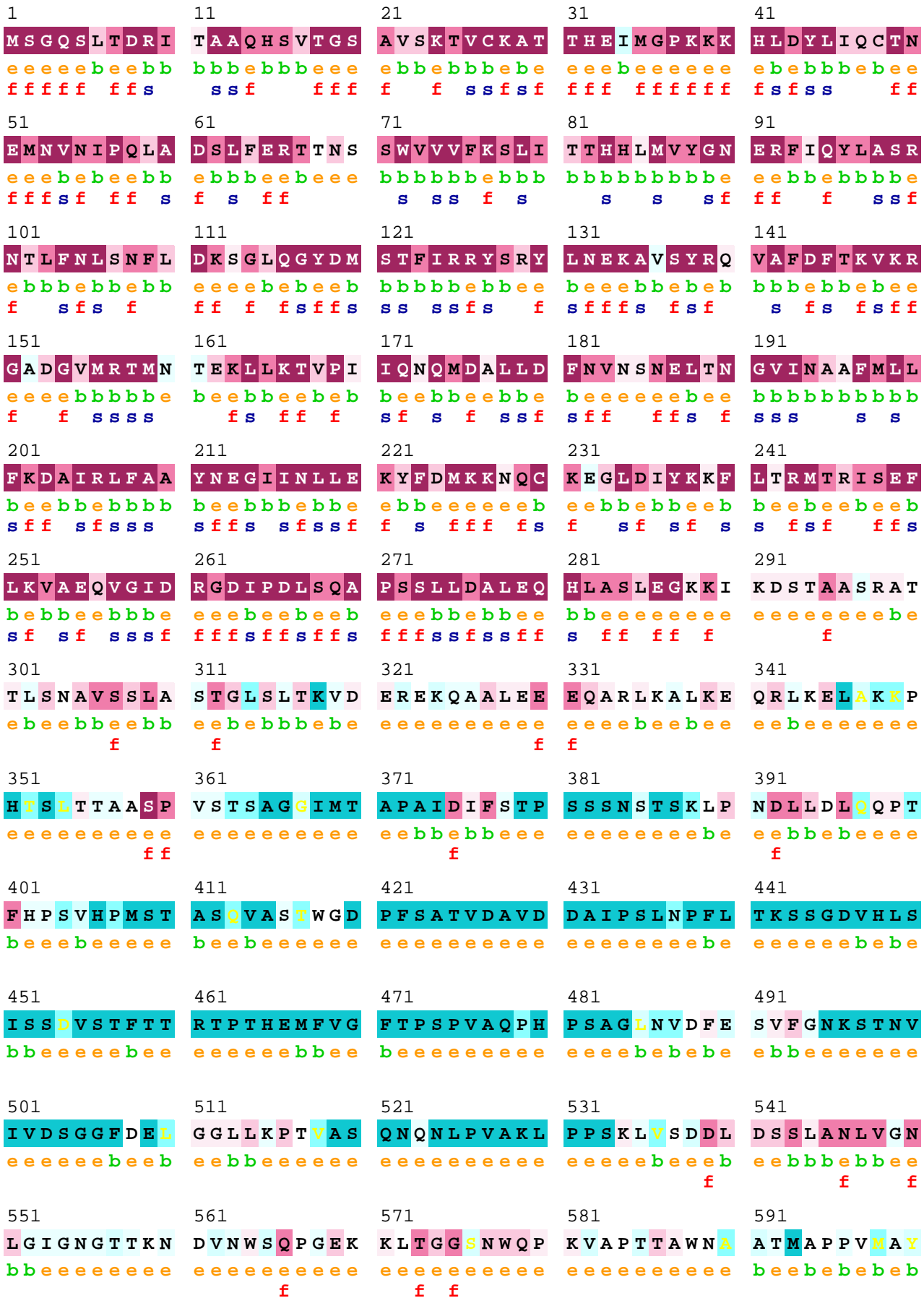

Supplement: Figure S2 — The result file was generated by ConSurf server. [file peerj-07-7667-s002.pdf]
