# Supplementary material for: Computational analysis of functional SNPs in Alzheimer’s disease-associated endocytosis genes
Source: PeerJ. 2019 Sep 30;7:e7667. doi: 10.7717/peerj.7667 (PMC6776068; doi:10.7717/peerj.7667)
Supplement: Figure S3 [file peerj-07-7667-s003.pdf]

ConSurf Results

```
1            11           21           31           41
MRKRWACWSG   SDAPGGCGGG   CGRRRRRSRR   KRAASEEERM   AFSKGFRIYH
eeeebbbbeb   eeeeeebebe   eeeeeeeeee   eeebeeeeeb   bbeebbebbe
                                                s   s  f

51           61           71           81           91
KLDPPPFSLI   VETRHKEECL   MFESGAVAVL   SSAEKEAIKG   TYSKVLDAYG
eeeeeebbbb   beeeeeeebb   bbeeebbbbb   eeeeeeebee   ebeebbebbb
f      s      f f           ff            f               f  s

101          111          121          131          141
LLGVLRLNLG   DTMLHYLVLV   TGCMSVGKIQ   ESEVFRVTST   EFISLRIDSS
bbebbebeee   eebbbbbbbb   bbbbeebebe   eeebbebbbe   ebbebeeeee
 sf s               ss     ss ffsf      ff  f   f      fs

151          161          171          181          191
DEDRISEVRK   VLNSGNFYFA   WSASGISLDL   SLNAHRSMQE   QTTDNRFFWN
eeeebeebee   bbeebbbbbb   beeeeeebeb   bbebeeeeee   eeeeeebbbb
         f     ffs sss            f    s   ff          f fs  s

201          211          221          231          241
QSLHLHLKHY   GVNCDDWLLR   LMCGGVEIRT   IYAAHKQAKA   CLISRLSCER
bbbebbbeeb   ebebeebbbe   bbbebbebeb   bbbeeeebeb   bbbbebbbee
   f               s        sf sf fs    s    f f       sf s ff

251          261          271          281          291
AGTRFNVRGT   NDDGHVANFV   ETEQVVYLDD   SVSSFIQIRG   SVPLFWEQPG
bebebebebe   eeeeebbebb   ebeebbbbee   ebbbbbebeb   ebebbbeeee
sfsfs sfs    ff f ssfss   fsff    f       s  f fs   fsfsssffff

301          311          321          331          341
LQVGSHRVRM   SRGFEANAPA   FDRHFRTLKN   LYGKQIIVNL   LGSKEGEHML
eebbeeebeb   beebebebeb   beebbebbee   eeeeebbbbb   bbeeeeeebb
 fssff        ff f f  s     fs          ff f   ss   s  ffff  s

351          361          371          381          391
SKAFQSHLKA   SEHAADIQMV   NFDYHQMVKG   GKAEKLHSVL   KPQVQKFLDY
eebbeeebee   eeeeeebebb   bbbbeeebee   eeeeebeebb   eeebeebbeb
f     f      f             ss f             s   s

401          411          421          431          441
GFFYFNGSEV   QRCQSGTVRT   NCLDCLDRTN   SVQAFLGLEM   LAKQLEALGL
ebbbeeeeee   eeeeeeebeb   ebbebbeeee   bbebbbbbeb   beeebeebee
                f f  f    fssfssffff    sf          s  f

451          461          471          481          491
AEKPQLVTRF   QEVFRSMWSV   NGDSISKIYA   GTGALEGKAK   LKDGARSVTR
eeeeebbeeb   eebbebbbbe   eeeebbebbb   beebbeeebe   beeebeebbe
  f     fs    f    ss     fff  sf ss   sffs ff  f    fffsff  f

501          511          521          531          541
TIQNNFFDSS   KQEAIDVLLL   GNTLNSDLAD   KARALLTTGS   LRVSEQTLQS
bbeeebeeeb   eeebbebbbb   bebbeeebee   ebebbbeebe   bebbeebbee
ssfffsff     fffs    ss   s    f  ff     f s                 f

551          561          571          581          591
ASSKVLKSMC   ENFYKYSKPK   KIRVCVGTWN   VNGGKQFRSI   AFKNQTLTDW
beeebbeebb   eeeeeeeeee   ebebbbbbee   beeeeebebe   bbeeeebbeb
 f   s             f             s f   sfffff fsf      f f  f
```

```
601         611         621         631         641
LLDAPKLAGI  QEFQDKRSKP  TDIFAIGFEE  MVELNAGNIV  SASTTNQKLW
beebeeeeee  eeeeeeeeee  bebbbbbbee  bbebebbbbb  bbeeeeeebb
sff                      f  s ssff  ssfsfs ss     f  fff s

651         661         671         681         691
AVELQKTISR  DNKYVLLASE  QLVGVCLFVF  IRPQHAPFIR  DVAVDTVKTG
beebeeebee  eeebbbbbbe  ebbbbbbbbb  bbeeebebbe  ebbbeebeeb
  f ff  ff  f  s        fssssss  s   sf f    f  fss ffsffs

701         711         721         731         741
MGGATGNKGA  VAIRMLFHTT  SLCFVCSHFA  AGQSQVKERN  EDFIEIARKL
eeeeeeeeeb  bbbbbbbbbb  bbbbbbbbbe  eeeeebeeee  eebeebbeeb
ffff ffff     ss        s  s s s f  fffff  fff   f  fs

751         761         771         781         791
SFPMGRMLFS  HDYVFWCGDF  NYRIDLPNEE  VKELIRQQNW  DSLIAGDQLI
bbebbebbbb  eebbbbbbeb  ebebebeeee  beebbeeeeb  eebeebeebe
 sf  f      ff  ssssfs  fsf f        f                ffs

801         811         821         831         841
NQKNAGQVFR  GFLEGKVTFA  PTYKYDLFSD  DYDTSEKCRT  PAWTDRVLWR
eeeeeeebbe  ebeeeebebe  eebeeebbee  eeeeeeebee  eebbeebbbe
 f      s      ff   s   ffsfff  f   fffffff f   ffssff s

851         861         871         881         891
RRKWPFDRSA  EDLDLLNASF  QDESKILYTW  TPGTLLHYGR  AELKTSDHRP
eeeeeeeeeb  eebebbeeeb  eeeeeeeeee  eeeebebbbe  bebeeeeeee
f                                     f    s f   fsfffffff

901         911         921         931         941
VVALIDIDIF  EVEAEERQNI  YKEVIAVQGP  PDGTVLVSIK  SSLPENNFFD
bbbbbebebb  ebeeeeeeeb  beebbeeeee  eebebbbbbe  eeeeeeeebe
s    f            f             ff   f f

951         961         971         981         991
DALIDELLQQ  FASFGEVILI  RFVEDKMWVT  FLEGSSALNV  LSLNGKELLN
eebbeebbee  beebbebbbb  bbbeeebbbb  beebeebbeb  beeeeeebee
f               s   s   s        s  s     ss

1001        1011        1021        1031        1041
RTITIALKSP  DWIKNLEEEM  SLEKISIALP  SSTSSTLLGE  DAEVAADFDM
eebebebeee  ebbeebeeeb  eeeeeeeebe  eeeeeebbee  ebebbeeeee
             s      f                        f

1051        1061        1071        1081        1091
EGDVDDYSAE  VEELLPQHLQ  PSSSSGLGTS  PSSSPRTSPC  QSPTISEGPV
eeeeeeeeee  eeebbeeebe  eeeeeeeeee  eeeeeeeeee  eeeeeeeeee

1101        1111        1121        1131        1141
PSLPIRPSRA  PSRTPGPPSA  QSSPIDAQPA  TPLPQKDPAQ  PLEPKRPPPP
eeeeeeeeee  eeeeeeeeee  eeeeeeeeee  eeeeeeeeee  eeeeeeeeee

1151        1161        1171        1181        1191
RPVAPPTRPA  PPQRPPPPSG  ARSPAPTRKE  FGGIGAPPSP  GVARREMEAP
eeeeeeeeee  eeeeeeeeee  eeeeeeeeee  eeeeeeeeee  eeeeeeeeee
 f   f      f   f
```

```
1201        1211        1221        1231        1241
KSPGTTRKDN  IGRSQPSPQA  GLAGPGPAGY  STARPTIPPR  AGVISAPQSH
eeeeeeeeee  eeeeeeeeee  eeeeeeeeee  eeeeeebeee  bbebebeeee
                                             f

1251        1261        1271        1281        1291
ARASAGRLTP  ESQSKTSETS  KGSTFLPEPL  KPQAAFPPQS  SLPPPAQRLQ
eeeeeeeeee  eeeeeeeeee  eeeeeeeeee  eeeeeeeeee  eeeeeeeeee

1301        1311        1321        1331        1341
EPLVPVAAPM  PQSGPQPNLE  TPPQPPPRSR  SSHSLPSEAS  SQPQQEQPSG
eeeeeebeee  eeeeeeeeee  eeeeeeeeee  eeeebeeeee  eeeeeeeeee
                                    f
```

The conservation scale:

1	2	3	4	5	6	7	8	9
Variable				Average			Conserved	

e - An exposed residue according to the neural-network algorithm.

b - A buried residue according to the neural-network algorithm.

f - A predicted functional residue (highly conserved and exposed).

s - A predicted structural residue (highly conserved and buried).

X - Insufficient data - the calculation for this site was performed on less than 10% of the sequences.
