# Supplementary material for: Computational analysis of functional SNPs in Alzheimer’s disease-associated endocytosis genes
Source: PeerJ. 2019 Sep 30;7:e7667. doi: 10.7717/peerj.7667 (PMC6776068; doi:10.7717/peerj.7667)
Supplement: Figure S4 — The result file was generated by ConSurf server. [file peerj-07-7667-s004.pdf]

ConSurf Results

1	11	21	31	41
MVEAIVEFDY	QAQHDDELTI	SVGEIITNIR	KEDGGWWEGQ	INGRRGLFPD
eeebbbebeb	eeeeeeebbb	ebbebbeebe	eeeeeebebe	eeeeeebbee
fff sf s	f f ffsss	s s fs	f ff fsf	f ff sff

51	61	71	81	91
NFVREIKKEM	KKDPLTNKAP	EKPLHEVPSG	NSLLSSETIL	RTNKRGERRR
ebbeebeeee	eeeeeeeeee	eeeeeeeeee	ebeeeeeeee	eeeeeeeeee
fssff f		f		f

101	111	121	131	141
RRCQVAFSYL	PQNDDELELK	VGDIIEVVGE	VEEGWWEGVL	NGKTGMFPSN
eebebbbeeb	eeeeeebebe	beebbebbee	beebbbebee	eeeeeebeee
ffs f	fff ffsfsf	f f	sffss s	ff f sfff

151	161	171	181	191
FIKELSGESD	ELGISQDEQL	SKSSLRETTG	SESDGGDSSS	TKSEGANGTV
bbeebeeeee	eeeeeeeeee	eeeeeeeeee	eeeeeeeeee	eeeeeeeeeb
ss f			f f	f

201	211	221	231	241
ATAAIQPKKV	KGVGFGDIFK	DKPIKLRPRS	IEVENDFLPV	EKTIGKKLPA
eeeeeeeeeb	eebbbbebbe	eeeeebeeee	eeeeeeeeee	eeeeeeeeee
f ff	f sssfssf	ffffs f		f

251	261	271	281	291
TTATPDSSKT	EMDSRTKSKD	YCKVIFPYEA	QNDDELTIKE	GDIVTLINKD
eeeeeeeeee	eeeeeeeeee	bbebbbeeee	eeeeebebee	eebbbbbbee
f	f ff	sf	f ffs f	f f

301	311	321	331	341
CIDVGWWEGE	LNGRRGVFPD	NFVKLLPPDF	EKEGNRPKKP	PPPSAPVIKQ
eeeebbbebe	eeeeeeeeee	ebbebbeeeb	eeeeeeeeee	eeeeeeeeee
f s s sf	ff fffff	fss	f fffff	fff f

351	361	371	381	391
GAGTTERKHE	IKKIPPERPE	MLPNRTEEKE	RPEREPKLDL	QKPSVPAIPP
eeeeeeeeee	beeeeeeeee	eeeeeeeeee	eeeeeeeeee	eeeebeeeee
f	f f ff		f	ff f f

401	411	421	431	441
KKPRPPKTNS	LSRPGALPPR	RPERPVGPLT	HTRGDSPKID	LAGSSLSGIL
eeeeeeeeee	eeeeeeeeee	eeeeeeeeee	eeeeeeeeee	eeeeeeebeb
fff f		ff ff		

451	461	471	481	491
DKDLSDRSND	IDLEGFDSVV	SSTEKLSHPT	TSRPKATGRR	PPSQSLTSSS
eeeeeeeeee	bebeebeebb	ebeeebeeee	eeeeeeeeee	eeeebeeeee
	f f f	fs fff	ffff fff	ff f f

501	511	521	531	541
LSSPDIFDSP	SPEEDKEEHI	SLAHRGVDAS	KKTSKTVTIS	QVSDNKASLP
eeeeeeeeee	eeeeeeeeee	eeeeeeeeee	eeeeeebebb	ebeeeeeeee

551	561	571	581	591
PKPGTMAAGG	GGPAPLSSAA	PSPLSSSLGT	AGHRANSPSL	FGTEGKPKME
eeeeeeeeee	eeeeebeeee	eeebeeeeee	eeeeeeeeee	eeeeeeeeee
			f	

```
601         611         621         631         641
PAASSQAAVE  ELRTQVRELR  SIIETMKDQQ  KREIKQLLSE  LDEEKKIRLR
eeeeeeeebe  ebeeebeebe  ebbeebeeee  eeebeebbee  beeeeeeebe
             s  f             f ff    f   s     sfff   f

651         661
LQMEVNDIKK  ALQSK
bebebeebee  eeeee
 f fs   f      ff
```

The conservation scale:

1	2	3	4	5	6	7	8	9
Variable				Average			Conserved	

- e - An exposed residue according to the neural-network algorithm.
- b - A buried residue according to the neural-network algorithm.
- f - A predicted functional residue (highly conserved and exposed).
- s - A predicted structural residue (highly conserved and buried).
