# Supplementary material for: Computational analysis of functional SNPs in Alzheimer’s disease-associated endocytosis genes
Source: PeerJ. 2019 Sep 30;7:e7667. doi: 10.7717/peerj.7667 (PMC6776068; doi:10.7717/peerj.7667)
Supplement: Table S1 [file peerj-07-7667-s005.docx]

**Supplemental Table S1. Possible consequences of splice site variants predicted by MaxEntScan.**

| **Consensus score** | **Splicing?** | **Score difference between wild type and mutant sequences** | **Consequences** |
| --- | --- | --- | --- |
| Above 3 | Donor or acceptor splice site | Increase | Splice site is retained |
|  |  | Decrease > 30% (-30%) | Splice site is broken |
| Below 3 | No splicing | Increase > 30% (+30%) | New splice site is created |
|  |  | Decrease | No splicing |
