# Supplementary material for: Computational analysis of functional SNPs in Alzheimer’s disease-associated endocytosis genes
Source: PeerJ. 2019 Sep 30;7:e7667. doi: 10.7717/peerj.7667 (PMC6776068; doi:10.7717/peerj.7667)
Supplement: Table S2 [file peerj-07-7667-s006.docx]

**Supplemental Table S2. UTR sequences information and the total number of variants of PICALM, SYNJ1 and SH3KBP1 genes.**

| **Gene** | **Locus** | **Region** | **Length (nt)** | **Number of variants** |
| --- | --- | --- | --- | --- |
| ***PICALM*** | 5HSAR013622 | 5’UTR | 286 | 17 |
|  | 3HSAR015450 | 3’UTR | 1604 | 18 |
|  | | | **Total** | **35** |
| ***SYNJ1*** | 5HSAR059427 | 5’UTR | 125 | 0 |
|  | 3HSAR062660 | 3’UTR | 3046 | 278 |
|  | | | **Total** | **278** |
| ***SH3KBP1*** | 5HSAR053069 | 5’UTR | 316 | 3 |
|  | 3HSAR056203 | 3’UTR | 2452 | 79 |
|  | | | **Total** | **82** |
