# Supplementary material for: Computational analysis of functional SNPs in Alzheimer’s disease-associated endocytosis genes
Source: PeerJ. 2019 Sep 30;7:e7667. doi: 10.7717/peerj.7667 (PMC6776068; doi:10.7717/peerj.7667)
Supplement: Table S5 — The result file was generated by PredictSNP2. [file peerj-07-7667-s009.pdf]

PredictSNP overview

Date: 2017/03/06
Genome version: GRCh38/hg38
PredictSNP version: 2.1

If you use PredictSNP and obtain scientific results that you publish, we would ask you to acknowledge the usage of PredictSNP by referencing the article below.

Bendl, J., Musil, M., Stourac, J., Zendulka, J., Damborsky, J., Brezovsky, J., 2016: PredictSNP2: A unified platform for accurately evaluating SNP effects by exploiting the different characteristics of variants in distinct genomic regions. *PLOS Computational Biology* 12: e1004962

	PredictSNP2		CADD		DANN		FATHMM		FunSeq2		GWAVA	
	Neu	Del	Neu	Del	Neu	Del	Neu	Del	Neu	Del	Neu	Del
Regulatory	0	0	0	0	0	0	0	0	0	0	0	0
Splicing	0	0	0	0	0	0	0	0	0	0	0	0
Missense	0	0	0	0	0	0	95	5	90	10	94	6
Synonymous	33	67	96	4	44	46	0	0	0	0	0	0
Nonsense	0	0	0	0	0	0	0	0	0	0	0	0
Others	0	0	0	0	0	0	0	0	0	0	0	0

Variant:	11 : 85974750, $G \to$C				
**Position:**	chr11:85974750	**Ref. allele:**	G	**Alt. allele:**	C
**Region:**	exonic	**Exonic. func.:**	synonymous	**Gene ID:**	PICALM
**RS number:**	-	**Clin. sign.:**	-	-	

Results

Tool:	PredictSNP2	CADD	DANN	FATHMM	FunSeq2	GWAVA
**Prediction:**	neutral	neutral	neutral	deleterious	neutral	neutral
**Score:**	-0.5876	5.7230	0.6814	0.8185	0.0000	0.2400
**Exp. accuracy:**	0.93	0.92	0.94	0.57	0.93	0.58

External links

[GenBank] [UCSC] [Ensemble]

Variant:	11 : 85974777, $T \to$C				
**Position:**	chr11:85974777	**Ref. allele:**	T	**Alt. allele:**	C
**Region:**	exonic	**Exonic. func.:**	synonymous	**Gene ID:**	PICALM
**RS number:**	rs200565799	**Clin. sign.:**	-	-	

Results

Tool:	PredictSNP2	CADD	DANN	FATHMM	FunSeq2	GWAVA
**Prediction:**	neutral	neutral	neutral	neutral	neutral	neutral
**Score:**	-1.0000	0.0090	0.3471	0.1083	0.0000	0.3000
**Exp. accuracy:**	0.96	0.95	0.97	0.87	0.93	0.57

External links

[dbSNP] [GenBank] [HaploReg] [RegulomeDB] [UCSC] [Ensemble]

Variant:	11 : 85974780, $C \to$T				
**Position:**	chr11:85974780	**Ref. allele:**	C	**Alt. allele:**	T
**Region:**	exonic	**Exonic. func.:**	synonymous	**Gene ID:**	PICALM
**RS number:**	-	**Clin. sign.:**	-	-	

Results

Tool:	PredictSNP2	CADD	DANN	FATHMM	FunSeq2	GWAVA
**Prediction:**	neutral	neutral	neutral	neutral	neutral	neutral
**Score:**	-1.0000	11.8800	0.5722	0.4095	0.0000	0.2800
**Exp. accuracy:**	0.96	0.83	0.97	0.72	0.93	0.55

External links

[GenBank] [UCSC] [Ensemble]

**Variant:** 11 : 85974795, $A \to$G					
**Position:**	chr11:85974795	**Ref. allele:**	A	**Alt. allele:**	G
**Region:**	exonic	**Exonic. func.:**	synonymous	**Gene ID:**	PICALM
**RS number:**	-	**Clin. sign.:**	-	-	

Results

**Tool:**	**PredictSNP2**	**CADD**	**DANN**	**FATHMM**	**FunSeq2**	**GWAVA**
**Prediction:**	neutral	neutral	neutral	neutral	neutral	neutral
**Score:**	-1.0000	0.4050	0.7045	0.6867	0.0000	0.2200
**Exp. accuracy:**	0.96	0.95	0.94	0.69	0.93	0.59

External links

[GenBank] [UCSC] [Ensemble]

**Variant:** 11 : 85974807, $T \to$C					
**Position:**	chr11:85974807	**Ref. allele:**	T	**Alt. allele:**	C
**Region:**	exonic	**Exonic. func.:**	synonymous	**Gene ID:**	PICALM
**RS number:**	-	**Clin. sign.:**	-	-	

Results

**Tool:**	**PredictSNP2**	**CADD**	**DANN**	**FATHMM**	**FunSeq2**	**GWAVA**
**Prediction:**	neutral	neutral	neutral	deleterious	neutral	neutral
**Score:**	-0.5853	5.0280	0.7135	0.8323	0.0000	0.2100
**Exp. accuracy:**	0.93	0.93	0.93	0.57	0.93	0.60

External links

[GenBank] [UCSC] [Ensemble]

**Variant:** 11 : 85981138, $A \to$C					
**Position:**	chr11:85981138	**Ref. allele:**	A	**Alt. allele:**	C
**Region:**	exonic	**Exonic. func.:**	synonymous	**Gene ID:**	PICALM
**RS number:**	rs76719109	**Clin. sign.:**	-	-	

Results

**Tool:**	**PredictSNP2**	**CADD**	**DANN**	**FATHMM**	**FunSeq2**	**GWAVA**
**Prediction:**	neutral	neutral	neutral	deleterious	neutral	neutral
**Score:**	-0.4724	6.8470	0.6763	0.9410	0.0000	0.2800
**Exp. accuracy:**	0.93	0.92	0.94	0.88	0.93	0.55

External links

[dbSNP] [GenBank] [HaploReg] [RegulomeDB] [UCSC] [Ensemble]

**Variant:** 11 : 85981138, $A \to$T					
**Position:**	chr11:85981138	**Ref. allele:**	A	**Alt. allele:**	T
**Region:**	exonic	**Exonic. func.:**	synonymous	**Gene ID:**	PICALM
**RS number:**	-	**Clin. sign.:**	-	-	

Results

**Tool:**	**PredictSNP2**	**CADD**	**DANN**	**FATHMM**	**FunSeq2**	**GWAVA**
**Prediction:**	neutral	neutral	neutral	deleterious	neutral	neutral
**Score:**	-0.4825	6.9340	0.6506	0.9278	0.0000	0.2800
**Exp. accuracy:**	0.93	0.92	0.96	0.82	0.93	0.55

External links

[GenBank] [UCSC] [Ensemble]

**Variant:** 11 : 85981150, $G \to$A					
**Position:**	chr11:85981150	**Ref. allele:**	G	**Alt. allele:**	A
**Region:**	exonic	**Exonic. func.:**	synonymous	**Gene ID:**	PICALM
**RS number:**	rs12295435	**Clin. sign.:**	-	-	

Results

**Tool:**	**PredictSNP2**	**CADD**	**DANN**	**FATHMM**	**FunSeq2**	**GWAVA**
**Prediction:**	neutral	neutral	neutral	neutral	neutral	neutral
**Score:**	-1.0000	12.0500	0.5906	0.4934	0.0000	0.2600
**Exp. accuracy:**	0.96	0.83	0.97	0.70	0.93	0.54

External links

[dbSNP] [GenBank] [HaploReg] [RegulomeDB] [UCSC] [Ensemble]

**Variant:** 11 : 85981153, $T \to$C					
**Position:**	chr11:85981153	**Ref. allele:**	T	**Alt. allele:**	C
**Region:**	exonic	**Exonic. func.:**	synonymous	**Gene ID:**	PICALM
**RS number:**	-	**Clin. sign.:**	-	-	

Results

**Tool:**	**PredictSNP2**	**CADD**	**DANN**	**FATHMM**	**FunSeq2**	**GWAVA**
**Prediction:**	neutral	neutral	neutral	deleterious	neutral	neutral
**Score:**	-0.5814	8.3060	0.7544	0.8430	0.0000	0.2400
**Exp. accuracy:**	0.93	0.92	0.90	0.57	0.93	0.58

External links

[GenBank] [UCSC] [Ensemble]

**Variant:** 11 : 85981222, $T \rightarrow$C					
**Position:**	chr11:85981222	**Ref. allele:**	T	**Alt. allele:**	C
**Region:**	exonic	**Exonic. func.:**	synonymous	**Gene ID:**	PICALM
**RS number:**	-	**Clin. sign.:**	-	-	

Results

**Tool:**	**PredictSNP2**	**CADD**	**DANN**	**FATHMM**	**FunSeq2**	**GWAVA**
**Prediction:**	neutral	neutral	neutral	deleterious	neutral	neutral
**Score:**	-0.5518	9.6600	0.7521	0.8790	0.0000	0.2400
**Exp. accuracy:**	0.93	0.91	0.90	0.64	0.93	0.58

External links

[GenBank] [UCSC] [Ensemble]

**Variant:** 11 : 85981753, $G \rightarrow$A					
**Position:**	chr11:85981753	**Ref. allele:**	G	**Alt. allele:**	A
**Region:**	exonic	**Exonic. func.:**	synonymous	**Gene ID:**	PICALM
**RS number:**	-	**Clin. sign.:**	-	-	

Results

**Tool:**	**PredictSNP2**	**CADD**	**DANN**	**FATHMM**	**FunSeq2**	**GWAVA**
**Prediction:**	neutral	neutral	neutral	deleterious	neutral	neutral
**Score:**	-0.5867	9.6870	0.6784	0.8295	0.0000	0.3900
**Exp. accuracy:**	0.93	0.91	0.94	0.57	0.93	0.52

External links

[GenBank] [UCSC] [Ensemble]

**Variant:** 11 : 85981762, $T \rightarrow$A					
**Position:**	chr11:85981762	**Ref. allele:**	T	**Alt. allele:**	A
**Region:**	exonic	**Exonic. func.:**	synonymous	**Gene ID:**	PICALM
**RS number:**	-	**Clin. sign.:**	-	-	

Results

**Tool:**	**PredictSNP2**	**CADD**	**DANN**	**FATHMM**	**FunSeq2**	**GWAVA**
**Prediction:**	neutral	neutral	neutral	deleterious	neutral	neutral
**Score:**	-0.4534	7.7210	0.6798	0.9539	0.0000	0.4200
**Exp. accuracy:**	0.93	0.91	0.94	0.92	0.93	0.54

External links

[GenBank] [UCSC] [Ensemble]

**Variant:** 11 : 85981765, $G \rightarrow$A					
**Position:**	chr11:85981765	**Ref. allele:**	G	**Alt. allele:**	A
**Region:**	exonic	**Exonic. func.:**	synonymous	**Gene ID:**	PICALM
**RS number:**	-	**Clin. sign.:**	-	-	

Results

**Tool:**	**PredictSNP2**	**CADD**	**DANN**	**FATHMM**	**FunSeq2**	**GWAVA**
**Prediction:**	neutral	neutral	neutral	neutral	neutral	neutral
**Score:**	-1.0000	11.1000	0.7879	0.2926	0.0000	0.4600
**Exp. accuracy:**	0.96	0.88	0.89	0.73	0.93	0.54

External links

[GenBank] [UCSC] [Ensemble]

**Variant:** 11 : 85981873, $G \rightarrow$A					
**Position:**	chr11:85981873	**Ref. allele:**	G	**Alt. allele:**	A
**Region:**	exonic	**Exonic. func.:**	synonymous	**Gene ID:**	PICALM
**RS number:**	rs371001564	**Clin. sign.:**	-	-	

Results

**Tool:**	**PredictSNP2**	**CADD**	**DANN**	**FATHMM**	**FunSeq2**	**GWAVA**
**Prediction:**	neutral	neutral	neutral	deleterious	neutral	neutral
**Score:**	-0.4266	13.5900	0.8618	0.9569	0.0000	0.4300
**Exp. accuracy:**	0.88	0.73	0.90	0.93	0.93	0.54

External links

[dbSNP] [GenBank] [RegulomeDB] [UCSC] [Ensemble]

**Variant:** 11 : 85981903, $G \rightarrow$A					
**Position:**	chr11:85981903	**Ref. allele:**	G	**Alt. allele:**	A
**Region:**	exonic	**Exonic. func.:**	synonymous	**Gene ID:**	PICALM
**RS number:**	-	**Clin. sign.:**	-	-	

Results

**Tool:**	**PredictSNP2**	**CADD**	**DANN**	**FATHMM**	**FunSeq2**	**GWAVA**
**Prediction:**	neutral	neutral	neutral	deleterious	neutral	neutral
**Score:**	-0.4624	12.2500	0.5484	0.9565	0.0000	0.4000
**Exp. accuracy:**	0.93	0.83	0.97	0.93	0.93	0.56

External links

[GenBank] [UCSC] [Ensemble]

**Variant:** 11 : 85981942, $A \rightarrow C$					
**Position:**	chr11:85981942	**Ref. allele:**	A	**Alt. allele:**	C
**Region:**	exonic	**Exonic. func.:**	synonymous	**Gene ID:**	PICALM
**RS number:**	-	**Clin. sign.:**	-	-	

Results

**Tool:**	**PredictSNP2**	**CADD**	**DANN**	**FATHMM**	**FunSeq2**	**GWAVA**
**Prediction:**	neutral	neutral	neutral	deleterious	neutral	neutral
**Score:**	-0.5683	0.1730	0.6676	0.8631	0.0000	0.4300
**Exp. accuracy:**	0.93	0.95	0.95	0.60	0.93	0.54

External links

[GenBank] [UCSC] [Ensemble]

**Variant:** 11 : 85981945, $A \rightarrow G$					
**Position:**	chr11:85981945	**Ref. allele:**	A	**Alt. allele:**	G
**Region:**	exonic	**Exonic. func.:**	synonymous	**Gene ID:**	PICALM
**RS number:**	-	**Clin. sign.:**	-	-	

Results

**Tool:**	**PredictSNP2**	**CADD**	**DANN**	**FATHMM**	**FunSeq2**	**GWAVA**
**Prediction:**	neutral	neutral	neutral	deleterious	neutral	neutral
**Score:**	-0.4553	9.1930	0.7753	0.9543	0.0000	0.4700
**Exp. accuracy:**	0.93	0.92	0.90	0.92	0.93	0.54

External links

[GenBank] [UCSC] [Ensemble]

**Variant:** 11 : 85981972, $T \rightarrow C$					
**Position:**	chr11:85981972	**Ref. allele:**	T	**Alt. allele:**	C
**Region:**	exonic	**Exonic. func.:**	synonymous	**Gene ID:**	PICALM
**RS number:**	-	**Clin. sign.:**	-	-	

Results

**Tool:**	**PredictSNP2**	**CADD**	**DANN**	**FATHMM**	**FunSeq2**	**GWAVA**
**Prediction:**	neutral	neutral	neutral	deleterious	neutral	neutral
**Score:**	-0.5184	7.4540	0.6746	0.9028	0.0000	0.4300
**Exp. accuracy:**	0.93	0.91	0.94	0.75	0.93	0.54

External links

[GenBank] [UCSC] [Ensemble]

**Variant:**	11 : 85983936, $T \to$G				
**Position:**	chr11:85983936	**Ref. allele:**	T	**Alt. allele:**	G
**Region:**	exonic	**Exonic. func.:**	synonymous	**Gene ID:**	PICALM
**RS number:**	-	**Clin. sign.:**	-	-	

Results

**Tool:**	**PredictSNP2**	**CADD**	**DANN**	**FATHMM**	**FunSeq2**	**GWAVA**
**Prediction:**	neutral	neutral	neutral	deleterious	neutral	deleterious
**Score:**	-0.4801	10.2000	0.6877	0.9275	0.0000	0.6000
**Exp. accuracy:**	0.93	0.88	0.94	0.82	0.93	0.64

External links

[GenBank] [UCSC] [Ensemble]

**Variant:**	11 : 85983951, $T \to$G				
**Position:**	chr11:85983951	**Ref. allele:**	T	**Alt. allele:**	G
**Region:**	exonic	**Exonic. func.:**	synonymous	**Gene ID:**	PICALM
**RS number:**	-	**Clin. sign.:**	-	-	

Results

**Tool:**	**PredictSNP2**	**CADD**	**DANN**	**FATHMM**	**FunSeq2**	**GWAVA**
**Prediction:**	neutral	neutral	neutral	deleterious	neutral	deleterious
**Score:**	-0.5321	7.6520	0.6450	0.8891	0.0000	0.6000
**Exp. accuracy:**	0.93	0.91	0.96	0.68	0.93	0.64

External links

[GenBank] [UCSC] [Ensemble]

**Variant:**	11 : 85983957, $T \to$C				
**Position:**	chr11:85983957	**Ref. allele:**	T	**Alt. allele:**	C
**Region:**	exonic	**Exonic. func.:**	synonymous	**Gene ID:**	PICALM
**RS number:**	rs149179479	**Clin. sign.:**	-	-	

Results

**Tool:**	**PredictSNP2**	**CADD**	**DANN**	**FATHMM**	**FunSeq2**	**GWAVA**
**Prediction:**	neutral	neutral	neutral	deleterious	neutral	deleterious
**Score:**	-0.5823	7.8000	0.7254	0.7912	0.0000	0.5500
**Exp. accuracy:**	0.93	0.91	0.93	0.57	0.93	0.66

External links

[dbSNP] [GenBank] [HaploReg] [RegulomeDB] [UCSC] [Ensemble]

**Variant:** 11 : 85990278, $A \to C$					
**Position:**	chr11:85990278	**Ref. allele:**	A	**Alt. allele:**	C
**Region:**	exonic	**Exonic. func.:**	synonymous	**Gene ID:**	PICALM
**RS number:**	-	**Clin. sign.:**	-	-	

Results

**Tool:**	**PredictSNP2**	**CADD**	**DANN**	**FATHMM**	**FunSeq2**	**GWAVA**
**Prediction:**	neutral	neutral	neutral	deleterious	neutral	neutral
**Score:**	-0.4171	0.6510	0.7134	0.9916	0.0000	0.4400
**Exp. accuracy:**	0.88	0.95	0.93	0.97	0.93	0.54

External links

[GenBank] [UCSC] [Ensemble]

**Variant:** 11 : 85990287, $A \to G$					
**Position:**	chr11:85990287	**Ref. allele:**	A	**Alt. allele:**	G
**Region:**	exonic	**Exonic. func.:**	synonymous	**Gene ID:**	PICALM
**RS number:**	-	**Clin. sign.:**	-	-	

Results

**Tool:**	**PredictSNP2**	**CADD**	**DANN**	**FATHMM**	**FunSeq2**	**GWAVA**
**Prediction:**	neutral	neutral	neutral	deleterious	neutral	neutral
**Score:**	-0.5284	0.1160	0.7023	0.8933	0.0000	0.4300
**Exp. accuracy:**	0.93	0.95	0.94	0.71	0.93	0.54

External links

[GenBank] [UCSC] [Ensemble]

**Variant:** 11 : 85990320, $A \to G$					
**Position:**	chr11:85990320	**Ref. allele:**	A	**Alt. allele:**	G
**Region:**	exonic	**Exonic. func.:**	synonymous	**Gene ID:**	PICALM
**RS number:**	-	**Clin. sign.:**	-	-	

Results

**Tool:**	**PredictSNP2**	**CADD**	**DANN**	**FATHMM**	**FunSeq2**	**GWAVA**
**Prediction:**	neutral	neutral	neutral	deleterious	neutral	neutral
**Score:**	-0.4707	6.7040	0.5990	0.9514	0.0000	0.4700
**Exp. accuracy:**	0.93	0.92	0.96	0.91	0.93	0.54

External links

[GenBank] [UCSC] [Ensemble]

**Variant:** 11 : 85990338, $G \to$A					
**Position:**	chr11:85990338	**Ref. allele:**	G	**Alt. allele:**	A
**Region:**	exonic	**Exonic. func.:**	synonymous	**Gene ID:**	PICALM
**RS number:**	-	**Clin. sign.:**	-	-	

Results

**Tool:**	**PredictSNP2**	**CADD**	**DANN**	**FATHMM**	**FunSeq2**	**GWAVA**
**Prediction:**	neutral	neutral	neutral	deleterious	neutral	deleterious
**Score:**	-0.5100	9.7780	0.7530	0.9068	0.0000	0.5000
**Exp. accuracy:**	0.93	0.91	0.90	0.75	0.93	0.51

External links

[GenBank] [UCSC] [Ensemble]

**Variant:** 11 : 85990344, $A \to$G					
**Position:**	chr11:85990344	**Ref. allele:**	A	**Alt. allele:**	G
**Region:**	exonic	**Exonic. func.:**	synonymous	**Gene ID:**	PICALM
**RS number:**	-	**Clin. sign.:**	-	-	

Results

**Tool:**	**PredictSNP2**	**CADD**	**DANN**	**FATHMM**	**FunSeq2**	**GWAVA**
**Prediction:**	neutral	neutral	neutral	deleterious	neutral	?
**Score:**	-0.4148	4.3540	0.7340	0.9908	0.0000	0.4900
**Exp. accuracy:**	0.88	0.94	0.91	0.97	0.93	0.43

External links

[GenBank] [UCSC] [Ensemble]

**Variant:** 11 : 85990368, $A \to$G					
**Position:**	chr11:85990368	**Ref. allele:**	A	**Alt. allele:**	G
**Region:**	exonic	**Exonic. func.:**	synonymous	**Gene ID:**	PICALM
**RS number:**	-	**Clin. sign.:**	-	-	

Results

**Tool:**	**PredictSNP2**	**CADD**	**DANN**	**FATHMM**	**FunSeq2**	**GWAVA**
**Prediction:**	neutral	neutral	neutral	deleterious	neutral	?
**Score:**	-0.5362	8.6900	0.5310	0.9008	0.0000	0.4900
**Exp. accuracy:**	0.93	0.92	0.97	0.71	0.93	0.43

External links

[GenBank] [UCSC] [Ensemble]

**Variant:** 11 : 85990371, $A \to C$					
**Position:**	chr11:85990371	**Ref. allele:**	A	**Alt. allele:**	C
**Region:**	exonic	**Exonic. func.:**	synonymous	**Gene ID:**	PICALM
**RS number:**	-	**Clin. sign.:**	-	-	

Results

**Tool:**	**PredictSNP2**	**CADD**	**DANN**	**FATHMM**	**FunSeq2**	**GWAVA**
**Prediction:**	neutral	neutral	neutral	deleterious	neutral	?
**Score:**	-0.5219	0.6420	0.7202	0.9000	0.0000	0.4900
**Exp. accuracy:**	0.93	0.95	0.93	0.71	0.93	0.43

External links

[GenBank] [UCSC] [Ensemble]

**Variant:** 11 : 85990371, $A \to G$					
**Position:**	chr11:85990371	**Ref. allele:**	A	**Alt. allele:**	G
**Region:**	exonic	**Exonic. func.:**	synonymous	**Gene ID:**	PICALM
**RS number:**	-	**Clin. sign.:**	-	-	

Results

**Tool:**	**PredictSNP2**	**CADD**	**DANN**	**FATHMM**	**FunSeq2**	**GWAVA**
**Prediction:**	neutral	neutral	neutral	deleterious	neutral	?
**Score:**	-0.5482	6.9260	0.7300	0.8794	0.0000	0.4900
**Exp. accuracy:**	0.93	0.92	0.91	0.64	0.93	0.43

External links

[GenBank] [UCSC] [Ensemble]

**Variant:** 11 : 85996869, $T \to C$					
**Position:**	chr11:85996869	**Ref. allele:**	T	**Alt. allele:**	C
**Region:**	exonic	**Exonic. func.:**	synonymous	**Gene ID:**	PICALM
**RS number:**	rs151035490	**Clin. sign.:**	-	-	

Results

**Tool:**	**PredictSNP2**	**CADD**	**DANN**	**FATHMM**	**FunSeq2**	**GWAVA**
**Prediction:**	neutral	neutral	neutral	neutral	neutral	deleterious
**Score:**	-1.0000	6.9350	0.7513	0.7689	0.0000	0.5600
**Exp. accuracy:**	0.96	0.92	0.90	0.69	0.93	0.69

External links

[dbSNP] [GenBank] [HaploReg] [RegulomeDB] [UCSC] [Ensemble]

**Variant:** 11 : 85996869, $T \to$G					
**Position:**	chr11:85996869	**Ref. allele:**	T	**Alt. allele:**	G
**Region:**	exonic	**Exonic. func.:**	synonymous	**Gene ID:**	PICALM
**RS number:**	-	**Clin. sign.:**	-	-	

Results

**Tool:**	**PredictSNP2**	**CADD**	**DANN**	**FATHMM**	**FunSeq2**	**GWAVA**
**Prediction:**	neutral	neutral	neutral	deleterious	neutral	deleterious
**Score:**	-0.5621	8.6160	0.7347	0.8684	0.0000	0.5600
**Exp. accuracy:**	0.93	0.92	0.91	0.60	0.93	0.69

External links

[GenBank] [UCSC] [Ensemble]

**Variant:** 11 : 85996890, $C \to$T					
**Position:**	chr11:85996890	**Ref. allele:**	C	**Alt. allele:**	T
**Region:**	exonic	**Exonic. func.:**	synonymous	**Gene ID:**	PICALM
**RS number:**	rs372958249	**Clin. sign.:**	-	-	

Results

**Tool:**	**PredictSNP2**	**CADD**	**DANN**	**FATHMM**	**FunSeq2**	**GWAVA**
**Prediction:**	neutral	neutral	neutral	deleterious	neutral	deleterious
**Score:**	-0.4350	13.7700	0.7898	0.9566	0.0000	0.5300
**Exp. accuracy:**	0.88	0.73	0.89	0.93	0.93	0.64

External links

[dbSNP] [GenBank] [RegulomeDB] [UCSC] [Ensemble]

**Variant:** 11 : 85996893, $C \to$T					
**Position:**	chr11:85996893	**Ref. allele:**	C	**Alt. allele:**	T
**Region:**	exonic	**Exonic. func.:**	synonymous	**Gene ID:**	PICALM
**RS number:**	-	**Clin. sign.:**	-	-	

Results

**Tool:**	**PredictSNP2**	**CADD**	**DANN**	**FATHMM**	**FunSeq2**	**GWAVA**
**Prediction:**	neutral	neutral	neutral	neutral	neutral	deleterious
**Score:**	-1.0000	12.7100	0.5206	0.0505	0.0000	0.5400
**Exp. accuracy:**	0.96	0.79	0.97	0.94	0.93	0.62

External links

[GenBank] [UCSC] [Ensemble]

**Variant:** 11 : 85996908, $A \to G$					
**Position:**	chr11:85996908	**Ref. allele:**	A	**Alt. allele:**	G
**Region:**	exonic	**Exonic. func.:**	synonymous	**Gene ID:**	PICALM
**RS number:**	-	**Clin. sign.:**	-	-	

Results

**Tool:**	**PredictSNP2**	**CADD**	**DANN**	**FATHMM**	**FunSeq2**	**GWAVA**
**Prediction:**	neutral	neutral	neutral	neutral	neutral	deleterious
**Score:**	-1.0000	5.1300	0.5613	0.6872	0.0000	0.5000
**Exp. accuracy:**	0.96	0.93	0.97	0.69	0.93	0.51

External links

[GenBank] [UCSC] [Ensemble]

**Variant:** 11 : 85996917, $C \to A$					
**Position:**	chr11:85996917	**Ref. allele:**	C	**Alt. allele:**	A
**Region:**	exonic	**Exonic. func.:**	synonymous	**Gene ID:**	PICALM
**RS number:**	-	**Clin. sign.:**	-	-	

Results

**Tool:**	**PredictSNP2**	**CADD**	**DANN**	**FATHMM**	**FunSeq2**	**GWAVA**
**Prediction:**	neutral	neutral	neutral	deleterious	neutral	deleterious
**Score:**	-0.4656	15.2900	0.7052	0.9333	0.0000	0.5000
**Exp. accuracy:**	0.93	0.73	0.93	0.86	0.93	0.51

External links

[GenBank] [UCSC] [Ensemble]

**Variant:** 11 : 85996923, $T \to C$					
**Position:**	chr11:85996923	**Ref. allele:**	T	**Alt. allele:**	C
**Region:**	exonic	**Exonic. func.:**	synonymous	**Gene ID:**	PICALM
**RS number:**	-	**Clin. sign.:**	-	-	

Results

**Tool:**	**PredictSNP2**	**CADD**	**DANN**	**FATHMM**	**FunSeq2**	**GWAVA**
**Prediction:**	neutral	neutral	neutral	deleterious	neutral	deleterious
**Score:**	-0.5841	7.9990	0.7160	0.8225	0.0000	0.5400
**Exp. accuracy:**	0.93	0.91	0.93	0.57	0.93	0.62

External links

[GenBank] [UCSC] [Ensemble]

**Variant:** 11 : 86000660, G→A					
**Position:**	chr11:86000660	**Ref. allele:**	G	**Alt. allele:**	A
**Region:**	exonic	**Exonic. func.:**	synonymous	**Gene ID:**	PICALM
**RS number:**	-	**Clin. sign.:**	-	-	

Results

**Tool:**	**PredictSNP2**	**CADD**	**DANN**	**FATHMM**	**FunSeq2**	**GWAVA**
**Prediction:**	neutral	neutral	neutral	deleterious	neutral	deleterious
**Score:**	-0.5888	11.7200	0.6518	0.8446	0.0000	0.5600
**Exp. accuracy:**	0.93	0.83	0.96	0.57	0.93	0.69

External links

[GenBank] [UCSC] [Ensemble]

**Variant:** 11 : 86000660, G→T					
**Position:**	chr11:86000660	**Ref. allele:**	G	**Alt. allele:**	T
**Region:**	exonic	**Exonic. func.:**	synonymous	**Gene ID:**	PICALM
**RS number:**	-	**Clin. sign.:**	-	-	

Results

**Tool:**	**PredictSNP2**	**CADD**	**DANN**	**FATHMM**	**FunSeq2**	**GWAVA**
**Prediction:**	neutral	neutral	neutral	neutral	neutral	deleterious
**Score:**	-1.0000	10.1300	0.6781	0.7739	0.0000	0.5600
**Exp. accuracy:**	0.96	0.88	0.94	0.69	0.93	0.69

External links

[GenBank] [UCSC] [Ensemble]

**Variant:** 11 : 86000678, G→A					
**Position:**	chr11:86000678	**Ref. allele:**	G	**Alt. allele:**	A
**Region:**	exonic	**Exonic. func.:**	synonymous	**Gene ID:**	PICALM
**RS number:**	rs145725396	**Clin. sign.:**	-	-	

Results

**Tool:**	**PredictSNP2**	**CADD**	**DANN**	**FATHMM**	**FunSeq2**	**GWAVA**
**Prediction:**	neutral	neutral	neutral	deleterious	neutral	deleterious
**Score:**	-0.4137	13.9400	0.6360	0.9874	0.0000	0.5700
**Exp. accuracy:**	0.88	0.73	0.97	0.97	0.93	0.68

External links

[dbSNP] [GenBank] [HaploReg] [RegulomeDB] [UCSC] [Ensemble]

**Variant:** 11 : 86000684, $T\rightarrow$G					
**Position:**	chr11:86000684	**Ref. allele:**	T	**Alt. allele:**	G
**Region:**	exonic	**Exonic. func.:**	synonymous	**Gene ID:**	PICALM
**RS number:**	rs200575473	**Clin. sign.:**	-	-	

Results

**Tool:**	**PredictSNP2**	**CADD**	**DANN**	**FATHMM**	**FunSeq2**	**GWAVA**
**Prediction:**	neutral	neutral	neutral	deleterious	neutral	deleterious
**Score:**	-0.4812	11.6500	0.6654	0.9279	0.0000	0.6300
**Exp. accuracy:**	0.93	0.83	0.95	0.82	0.93	0.66

External links

[dbSNP] [GenBank] [HaploReg] [RegulomeDB] [UCSC] [Ensemble]

**Variant:** 11 : 86000687, $A\rightarrow$G					
**Position:**	chr11:86000687	**Ref. allele:**	A	**Alt. allele:**	G
**Region:**	exonic	**Exonic. func.:**	synonymous	**Gene ID:**	PICALM
**RS number:**	-	**Clin. sign.:**	-	-	

Results

**Tool:**	**PredictSNP2**	**CADD**	**DANN**	**FATHMM**	**FunSeq2**	**GWAVA**
**Prediction:**	neutral	neutral	neutral	deleterious	neutral	deleterious
**Score:**	-0.5654	9.2110	0.7452	0.8623	0.0000	0.6100
**Exp. accuracy:**	0.93	0.91	0.91	0.57	0.93	0.64

External links

[GenBank] [UCSC] [Ensemble]

**Variant:** 11 : 86000696, $C\rightarrow$A					
**Position:**	chr11:86000696	**Ref. allele:**	C	**Alt. allele:**	A
**Region:**	exonic	**Exonic. func.:**	synonymous	**Gene ID:**	PICALM
**RS number:**	-	**Clin. sign.:**	-	-	

Results

**Tool:**	**PredictSNP2**	**CADD**	**DANN**	**FATHMM**	**FunSeq2**	**GWAVA**
**Prediction:**	neutral	deleterious	neutral	neutral	neutral	deleterious
**Score:**	-0.6078	16.4500	0.6252	0.4003	0.0000	0.6500
**Exp. accuracy:**	0.95	0.58	0.96	0.72	0.93	0.70

External links

[GenBank] [UCSC] [Ensemble]

Variant:	11 : 86000696, $C \to$T				
**Position:**	chr11:86000696	**Ref. allele:**	C	**Alt. allele:**	T
**Region:**	exonic	**Exonic. func.:**	synonymous	**Gene ID:**	PICALM
**RS number:**	-	**Clin. sign.:**	-	-	

Results

Tool:	PredictSNP2	CADD	DANN	FATHMM	FunSeq2	GWAVA
**Prediction:**	neutral	neutral	neutral	neutral	neutral	deleterious
**Score:**	-1.0000	13.3300	0.6099	0.5595	0.0000	0.6500
**Exp. accuracy:**	0.96	0.74	0.96	0.70	0.93	0.70

External links

[GenBank] [UCSC] [Ensemble]

Variant:	11 : 86000702, $T \to$G				
**Position:**	chr11:86000702	**Ref. allele:**	T	**Alt. allele:**	G
**Region:**	exonic	**Exonic. func.:**	synonymous	**Gene ID:**	PICALM
**RS number:**	-	**Clin. sign.:**	-	-	

Results

Tool:	PredictSNP2	CADD	DANN	FATHMM	FunSeq2	GWAVA
**Prediction:**	neutral	neutral	neutral	deleterious	neutral	deleterious
**Score:**	-0.5370	10.2900	0.5631	0.8959	0.0000	0.5800
**Exp. accuracy:**	0.93	0.88	0.97	0.71	0.93	0.65

External links

[GenBank] [UCSC] [Ensemble]

Variant:	11 : 86000714, $T \to$C				
**Position:**	chr11:86000714	**Ref. allele:**	T	**Alt. allele:**	C
**Region:**	exonic	**Exonic. func.:**	synonymous	**Gene ID:**	PICALM
**RS number:**	rs141849542	**Clin. sign.:**	-	-	

Results

Tool:	PredictSNP2	CADD	DANN	FATHMM	FunSeq2	GWAVA
**Prediction:**	neutral	neutral	neutral	neutral	neutral	deleterious
**Score:**	-1.0000	9.2410	0.7283	0.6247	0.0000	0.6000
**Exp. accuracy:**	0.96	0.91	0.93	0.69	0.93	0.64

External links

[dbSNP] [GenBank] [HaploReg] [RegulomeDB] [UCSC] [Ensemble]

**Variant:** 11 : 86000729, $A \rightarrow$G					
**Position:**	chr11:86000729	**Ref. allele:**	A	**Alt. allele:**	G
**Region:**	exonic	**Exonic. func.:**	synonymous	**Gene ID:**	PICALM
**RS number:**	rs201745810	**Clin. sign.:**	-	-	

Results

**Tool:**	**PredictSNP2**	**CADD**	**DANN**	**FATHMM**	**FunSeq2**	**GWAVA**
**Prediction:**	neutral	neutral	neutral	neutral	neutral	deleterious
**Score:**	-1.0000	9.8280	0.7121	0.2832	0.0000	0.6000
**Exp. accuracy:**	0.96	0.91	0.93	0.73	0.93	0.64

External links

[dbSNP] [GenBank] [HaploReg] [RegulomeDB] [UCSC] [Ensemble]

**Variant:** 11 : 86000762, $T \rightarrow$C					
**Position:**	chr11:86000762	**Ref. allele:**	T	**Alt. allele:**	C
**Region:**	exonic	**Exonic. func.:**	synonymous	**Gene ID:**	PICALM
**RS number:**	rs150582057	**Clin. sign.:**	-	-	

Results

**Tool:**	**PredictSNP2**	**CADD**	**DANN**	**FATHMM**	**FunSeq2**	**GWAVA**
**Prediction:**	neutral	neutral	neutral	deleterious	neutral	deleterious
**Score:**	-0.5452	8.5530	0.5867	0.8825	0.0000	0.6700
**Exp. accuracy:**	0.93	0.92	0.97	0.64	0.93	0.70

External links

[dbSNP] [GenBank] [HaploReg] [RegulomeDB] [UCSC] [Ensemble]

**Variant:** 11 : 86000768, $T \rightarrow$A					
**Position:**	chr11:86000768	**Ref. allele:**	T	**Alt. allele:**	A
**Region:**	exonic	**Exonic. func.:**	synonymous	**Gene ID:**	PICALM
**RS number:**	-	**Clin. sign.:**	-	-	

Results

**Tool:**	**PredictSNP2**	**CADD**	**DANN**	**FATHMM**	**FunSeq2**	**GWAVA**
**Prediction:**	neutral	neutral	neutral	deleterious	neutral	deleterious
**Score:**	-0.5501	11.6200	0.7071	0.8778	0.0000	0.6600
**Exp. accuracy:**	0.93	0.88	0.93	0.64	0.93	0.70

External links

[GenBank] [UCSC] [Ensemble]

**Variant:** 11 : 86000774, $C \rightarrow$T					
**Position:**	chr11:86000774	**Ref. allele:**	C	**Alt. allele:**	T
**Region:**	exonic	**Exonic. func.:**	synonymous	**Gene ID:**	PICALM
**RS number:**	rs372267972	**Clin. sign.:**	-	-	

Results

**Tool:**	**PredictSNP2**	**CADD**	**DANN**	**FATHMM**	**FunSeq2**	**GWAVA**
**Prediction:**	neutral	neutral	neutral	deleterious	neutral	deleterious
**Score:**	-0.5807	13.6800	0.6379	0.8103	0.0000	0.7400
**Exp. accuracy:**	0.93	0.73	0.97	0.57	0.93	0.70

External links

[dbSNP] [GenBank] [RegulomeDB] [UCSC] [Ensemble]

**Variant:** 11 : 86001062, $T \rightarrow$C					
**Position:**	chr11:86001062	**Ref. allele:**	T	**Alt. allele:**	C
**Region:**	exonic	**Exonic. func.:**	synonymous	**Gene ID:**	PICALM
**RS number:**	-	**Clin. sign.:**	-	-	

Results

**Tool:**	**PredictSNP2**	**CADD**	**DANN**	**FATHMM**	**FunSeq2**	**GWAVA**
**Prediction:**	neutral	neutral	neutral	deleterious	neutral	deleterious
**Score:**	-0.5280	4.1600	0.6360	0.8956	0.0000	0.5100
**Exp. accuracy:**	0.93	0.94	0.97	0.71	0.93	0.55

External links

[GenBank] [UCSC] [Ensemble]

**Variant:** 11 : 86001071, $T \rightarrow$A					
**Position:**	chr11:86001071	**Ref. allele:**	T	**Alt. allele:**	A
**Region:**	exonic	**Exonic. func.:**	synonymous	**Gene ID:**	PICALM
**RS number:**	-	**Clin. sign.:**	-	-	

Results

**Tool:**	**PredictSNP2**	**CADD**	**DANN**	**FATHMM**	**FunSeq2**	**GWAVA**
**Prediction:**	neutral	neutral	neutral	neutral	neutral	deleterious
**Score:**	-1.0000	15.3200	0.6531	0.5199	0.0000	0.5800
**Exp. accuracy:**	0.96	0.73	0.96	0.70	0.93	0.65

External links

[GenBank] [UCSC] [Ensemble]

**Variant:** 11 : 86001071, $T \rightarrow$G					
**Position:**	chr11:86001071	**Ref. allele:**	T	**Alt. allele:**	G
**Region:**	exonic	**Exonic. func.:**	synonymous	**Gene ID:**	PICALM
**RS number:**	-	**Clin. sign.:**	-	-	

Results

**Tool:**	**PredictSNP2**	**CADD**	**DANN**	**FATHMM**	**FunSeq2**	**GWAVA**
**Prediction:**	neutral	neutral	neutral	neutral	neutral	deleterious
**Score:**	-1.0000	13.1200	0.6518	0.5751	0.0000	0.5800
**Exp. accuracy:**	0.96	0.74	0.96	0.70	0.93	0.65

External links

[GenBank] [UCSC] [Ensemble]

**Variant:** 11 : 86001080, $C \rightarrow$T					
**Position:**	chr11:86001080	**Ref. allele:**	C	**Alt. allele:**	T
**Region:**	exonic	**Exonic. func.:**	synonymous	**Gene ID:**	PICALM
**RS number:**	-	**Clin. sign.:**	-	-	

Results

**Tool:**	**PredictSNP2**	**CADD**	**DANN**	**FATHMM**	**FunSeq2**	**GWAVA**
**Prediction:**	neutral	neutral	neutral	deleterious	neutral	deleterious
**Score:**	-0.5855	14.6800	0.5928	0.8509	0.0000	0.5600
**Exp. accuracy:**	0.93	0.73	0.96	0.57	0.93	0.69

External links

[GenBank] [UCSC] [Ensemble]

**Variant:** 11 : 86001095, $C \rightarrow$T					
**Position:**	chr11:86001095	**Ref. allele:**	C	**Alt. allele:**	T
**Region:**	exonic	**Exonic. func.:**	synonymous	**Gene ID:**	PICALM
**RS number:**	-	**Clin. sign.:**	-	-	

Results

**Tool:**	**PredictSNP2**	**CADD**	**DANN**	**FATHMM**	**FunSeq2**	**GWAVA**
**Prediction:**	neutral	neutral	neutral	deleterious	neutral	deleterious
**Score:**	-0.5079	13.8400	0.7642	0.9016	0.0000	0.5000
**Exp. accuracy:**	0.93	0.73	0.90	0.71	0.93	0.51

External links

[GenBank] [UCSC] [Ensemble]

**Variant:** 11 : 86001101, $G \rightarrow$C					
**Position:**	chr11:86001101	**Ref. allele:**	G	**Alt. allele:**	C
**Region:**	exonic	**Exonic. func.:**	synonymous	**Gene ID:**	PICALM
**RS number:**	-	**Clin. sign.:**	-	-	

Results

**Tool:**	**PredictSNP2**	**CADD**	**DANN**	**FATHMM**	**FunSeq2**	**GWAVA**
**Prediction:**	neutral	neutral	neutral	deleterious	neutral	deleterious
**Score:**	-0.4781	9.1710	0.6993	0.9416	0.0000	0.5100
**Exp. accuracy:**	0.93	0.92	0.94	0.88	0.93	0.55

External links

[GenBank] [UCSC] [Ensemble]

**Variant:** 11 : 86001106, $G \rightarrow$A					
**Position:**	chr11:86001106	**Ref. allele:**	G	**Alt. allele:**	A
**Region:**	exonic	**Exonic. func.:**	synonymous	**Gene ID:**	PICALM
**RS number:**	-	**Clin. sign.:**	-	-	

Results

**Tool:**	**PredictSNP2**	**CADD**	**DANN**	**FATHMM**	**FunSeq2**	**GWAVA**
**Prediction:**	neutral	neutral	neutral	deleterious	neutral	deleterious
**Score:**	-0.4577	12.2500	0.7478	0.9427	0.0000	0.5400
**Exp. accuracy:**	0.93	0.83	0.91	0.90	0.93	0.62

External links

[GenBank] [UCSC] [Ensemble]

**Variant:** 11 : 86001110, $T \rightarrow$A					
**Position:**	chr11:86001110	**Ref. allele:**	T	**Alt. allele:**	A
**Region:**	exonic	**Exonic. func.:**	synonymous	**Gene ID:**	PICALM
**RS number:**	-	**Clin. sign.:**	-	-	

Results

**Tool:**	**PredictSNP2**	**CADD**	**DANN**	**FATHMM**	**FunSeq2**	**GWAVA**
**Prediction:**	neutral	neutral	neutral	neutral	neutral	deleterious
**Score:**	-1.0000	12.7800	0.7079	0.6762	0.0000	0.5600
**Exp. accuracy:**	0.96	0.77	0.93	0.69	0.93	0.69

External links

[GenBank] [UCSC] [Ensemble]

**Variant:** 11 : 86001110, $T \rightarrow$C					
**Position:**	chr11:86001110	**Ref. allele:**	T	**Alt. allele:**	C
**Region:**	exonic	**Exonic. func.:**	synonymous	**Gene ID:**	PICALM
**RS number:**	rs139625756	**Clin. sign.:**	-	-	

Results

**Tool:**	**PredictSNP2**	**CADD**	**DANN**	**FATHMM**	**FunSeq2**	**GWAVA**
**Prediction:**	neutral	neutral	neutral	neutral	neutral	deleterious
**Score:**	-1.0000	9.0250	0.7043	0.2603	0.0000	0.5600
**Exp. accuracy:**	0.96	0.92	0.94	0.73	0.93	0.69

External links

[dbSNP] [GenBank] [HaploReg] [RegulomeDB] [UCSC] [Ensemble]

**Variant:** 11 : 86001116, $A \rightarrow$G					
**Position:**	chr11:86001116	**Ref. allele:**	A	**Alt. allele:**	G
**Region:**	exonic	**Exonic. func.:**	synonymous	**Gene ID:**	PICALM
**RS number:**	-	**Clin. sign.:**	-	-	

Results

**Tool:**	**PredictSNP2**	**CADD**	**DANN**	**FATHMM**	**FunSeq2**	**GWAVA**
**Prediction:**	neutral	neutral	neutral	deleterious	neutral	deleterious
**Score:**	-0.4813	9.6180	0.7092	0.9293	0.0000	0.5200
**Exp. accuracy:**	0.93	0.91	0.93	0.82	0.93	0.58

External links

[GenBank] [UCSC] [Ensemble]

**Variant:** 11 : 86001122, $T \rightarrow$A					
**Position:**	chr11:86001122	**Ref. allele:**	T	**Alt. allele:**	A
**Region:**	exonic	**Exonic. func.:**	synonymous	**Gene ID:**	PICALM
**RS number:**	rs111469150	**Clin. sign.:**	-	-	

Results

**Tool:**	**PredictSNP2**	**CADD**	**DANN**	**FATHMM**	**FunSeq2**	**GWAVA**
**Prediction:**	neutral	neutral	neutral	deleterious	neutral	deleterious
**Score:**	-0.4103	12.9700	0.7187	0.9720	0.0000	0.5100
**Exp. accuracy:**	0.88	0.77	0.93	0.97	0.93	0.55

External links

[dbSNP] [GenBank] [HaploReg] [RegulomeDB] [UCSC] [Ensemble]

**Variant:** 11 : 86001149, T→C					
**Position:**	chr11:86001149	**Ref. allele:**	T	**Alt. allele:**	C
**Region:**	exonic	**Exonic. func.:**	synonymous	**Gene ID:**	PICALM
**RS number:**	-	**Clin. sign.:**	-	-	

Results

**Tool:**	**PredictSNP2**	**CADD**	**DANN**	**FATHMM**	**FunSeq2**	**GWAVA**
**Prediction:**	neutral	neutral	neutral	deleterious	neutral	deleterious
**Score:**	-0.4747	7.8220	0.7392	0.9288	0.0000	0.5400
**Exp. accuracy:**	0.93	0.91	0.91	0.82	0.93	0.62

External links

[GenBank] [UCSC] [Ensemble]

**Variant:** 11 : 86003413, T→C					
**Position:**	chr11:86003413	**Ref. allele:**	T	**Alt. allele:**	C
**Region:**	exonic	**Exonic. func.:**	synonymous	**Gene ID:**	PICALM
**RS number:**	rs200195910	**Clin. sign.:**	-	-	

Results

**Tool:**	**PredictSNP2**	**CADD**	**DANN**	**FATHMM**	**FunSeq2**	**GWAVA**
**Prediction:**	neutral	neutral	neutral	deleterious	neutral	?
**Score:**	-0.5038	9.6710	0.7788	0.9053	0.0000	0.4900
**Exp. accuracy:**	0.93	0.91	0.90	0.75	0.93	0.43

External links

[dbSNP] [GenBank] [HaploReg] [RegulomeDB] [UCSC] [Ensemble]

**Variant:** 11 : 86003427, A→G					
**Position:**	chr11:86003427	**Ref. allele:**	A	**Alt. allele:**	G
**Region:**	exonic	**Exonic. func.:**	synonymous	**Gene ID:**	PICALM
**RS number:**	-	**Clin. sign.:**	-	-	

Results

**Tool:**	**PredictSNP2**	**CADD**	**DANN**	**FATHMM**	**FunSeq2**	**GWAVA**
**Prediction:**	neutral	neutral	neutral	deleterious	neutral	neutral
**Score:**	-0.4941	8.5750	0.7693	0.9135	0.0000	0.4600
**Exp. accuracy:**	0.93	0.92	0.90	0.77	0.93	0.54

External links

[GenBank] [UCSC] [Ensemble]

**Variant:** 11 : 86011033, $T \rightarrow$C					
**Position:**	chr11:86011033	**Ref. allele:**	T	**Alt. allele:**	C
**Region:**	exonic	**Exonic. func.:**	synonymous	**Gene ID:**	PICALM
**RS number:**	-	**Clin. sign.:**	-	-	

Results

**Tool:**	**PredictSNP2**	**CADD**	**DANN**	**FATHMM**	**FunSeq2**	**GWAVA**
**Prediction:**	neutral	neutral	neutral	deleterious	neutral	deleterious
**Score:**	-0.4795	9.1330	0.7432	0.9265	0.0000	0.7500
**Exp. accuracy:**	0.93	0.92	0.91	0.82	0.93	0.70

External links

[GenBank] [UCSC] [Ensemble]

**Variant:** 11 : 86011036, $A \rightarrow$C					
**Position:**	chr11:86011036	**Ref. allele:**	A	**Alt. allele:**	C
**Region:**	exonic	**Exonic. func.:**	synonymous	**Gene ID:**	PICALM
**RS number:**	-	**Clin. sign.:**	-	-	

Results

**Tool:**	**PredictSNP2**	**CADD**	**DANN**	**FATHMM**	**FunSeq2**	**GWAVA**
**Prediction:**	neutral	neutral	neutral	deleterious	neutral	deleterious
**Score:**	-0.5490	6.1350	0.8235	0.8672	0.0000	0.7200
**Exp. accuracy:**	0.93	0.93	0.90	0.60	0.93	0.70

External links

[GenBank] [UCSC] [Ensemble]

**Variant:** 11 : 86011129, $A \rightarrow$G					
**Position:**	chr11:86011129	**Ref. allele:**	A	**Alt. allele:**	G
**Region:**	exonic	**Exonic. func.:**	synonymous	**Gene ID:**	PICALM
**RS number:**	-	**Clin. sign.:**	-	-	

Results

**Tool:**	**PredictSNP2**	**CADD**	**DANN**	**FATHMM**	**FunSeq2**	**GWAVA**
**Prediction:**	neutral	neutral	neutral	deleterious	neutral	deleterious
**Score:**	-0.5982	2.8440	0.5645	0.8185	0.0000	0.6900
**Exp. accuracy:**	0.93	0.95	0.97	0.57	0.93	0.70

External links

[GenBank] [UCSC] [Ensemble]

**Variant:** 11 : 86012300, $T \rightarrow$C					
**Position:**	chr11:86012300	**Ref. allele:**	T	**Alt. allele:**	C
**Region:**	exonic	**Exonic. func.:**	synonymous	**Gene ID:**	PICALM
**RS number:**	rs375495535	**Clin. sign.:**	-	-	

Results

**Tool:**	**PredictSNP2**	**CADD**	**DANN**	**FATHMM**	**FunSeq2**	**GWAVA**
**Prediction:**	neutral	neutral	neutral	deleterious	neutral	neutral
**Score:**	-0.4585	4.5400	0.6037	0.9616	0.0000	0.3900
**Exp. accuracy:**	0.93	0.93	0.96	0.94	0.93	0.52

External links

[dbSNP] [GenBank] [RegulomeDB] [UCSC] [Ensemble]

**Variant:** 11 : 86012339, $C \rightarrow$A					
**Position:**	chr11:86012339	**Ref. allele:**	C	**Alt. allele:**	A
**Region:**	exonic	**Exonic. func.:**	synonymous	**Gene ID:**	PICALM
**RS number:**	-	**Clin. sign.:**	-	-	

Results

**Tool:**	**PredictSNP2**	**CADD**	**DANN**	**FATHMM**	**FunSeq2**	**GWAVA**
**Prediction:**	neutral	deleterious	neutral	deleterious	neutral	neutral
**Score:**	-0.1308	17.2300	0.7450	0.8174	0.0000	0.4400
**Exp. accuracy:**	0.88	0.58	0.91	0.57	0.93	0.54

External links

[GenBank] [UCSC] [Ensemble]

**Variant:** 11 : 86012351, $G \rightarrow$A					
**Position:**	chr11:86012351	**Ref. allele:**	G	**Alt. allele:**	A
**Region:**	exonic	**Exonic. func.:**	synonymous	**Gene ID:**	PICALM
**RS number:**	-	**Clin. sign.:**	-	-	

Results

**Tool:**	**PredictSNP2**	**CADD**	**DANN**	**FATHMM**	**FunSeq2**	**GWAVA**
**Prediction:**	neutral	neutral	neutral	deleterious	neutral	deleterious
**Score:**	-0.4679	13.8500	0.6356	0.9464	0.0000	0.5100
**Exp. accuracy:**	0.93	0.73	0.97	0.90	0.93	0.55

External links

[GenBank] [UCSC] [Ensemble]

**Variant:** 11 : 86012351, $G \to$C					
**Position:**	chr11:86012351	**Ref. allele:**	G	**Alt. allele:**	C
**Region:**	exonic	**Exonic. func.:**	synonymous	**Gene ID:**	PICALM
**RS number:**	-	**Clin. sign.:**	-	-	

Results

**Tool:**	**PredictSNP2**	**CADD**	**DANN**	**FATHMM**	**FunSeq2**	**GWAVA**
**Prediction:**	neutral	neutral	neutral	deleterious	neutral	deleterious
**Score:**	-0.4561	10.9400	0.6702	0.9575	0.0000	0.5100
**Exp. accuracy:**	0.93	0.88	0.94	0.93	0.93	0.55

External links

[GenBank] [UCSC] [Ensemble]

**Variant:** 11 : 86012354, $A \to$G					
**Position:**	chr11:86012354	**Ref. allele:**	A	**Alt. allele:**	G
**Region:**	exonic	**Exonic. func.:**	synonymous	**Gene ID:**	PICALM
**RS number:**	rs149334225	**Clin. sign.:**	-	-	

Results

**Tool:**	**PredictSNP2**	**CADD**	**DANN**	**FATHMM**	**FunSeq2**	**GWAVA**
**Prediction:**	neutral	neutral	neutral	deleterious	neutral	?
**Score:**	-0.5438	12.2000	0.6703	0.8787	0.0000	0.4800
**Exp. accuracy:**	0.93	0.83	0.94	0.64	0.93	0.43

External links

[dbSNP] [GenBank] [HaploReg] [RegulomeDB] [UCSC] [Ensemble]

**Variant:** 11 : 86012357, $A \to$G					
**Position:**	chr11:86012357	**Ref. allele:**	A	**Alt. allele:**	G
**Region:**	exonic	**Exonic. func.:**	synonymous	**Gene ID:**	PICALM
**RS number:**	rs79317992	**Clin. sign.:**	-	-	

Results

**Tool:**	**PredictSNP2**	**CADD**	**DANN**	**FATHMM**	**FunSeq2**	**GWAVA**
**Prediction:**	neutral	neutral	neutral	deleterious	neutral	neutral
**Score:**	-0.5084	10.0600	0.7306	0.9093	0.0000	0.4700
**Exp. accuracy:**	0.93	0.91	0.91	0.75	0.93	0.54

External links

[dbSNP] [GenBank] [HaploReg] [RegulomeDB] [UCSC] [Ensemble]

**Variant:** 11 : 86012363, $T \rightarrow$C					
**Position:**	chr11:86012363	**Ref. allele:**	T	**Alt. allele:**	C
**Region:**	exonic	**Exonic. func.:**	synonymous	**Gene ID:**	PICALM
**RS number:**	rs199846387	**Clin. sign.:**	-	-	

Results

**Tool:**	**PredictSNP2**	**CADD**	**DANN**	**FATHMM**	**FunSeq2**	**GWAVA**
**Prediction:**	neutral	neutral	neutral	deleterious	neutral	neutral
**Score:**	-0.4828	9.7910	0.6885	0.9264	0.0000	0.4100
**Exp. accuracy:**	0.93	0.91	0.94	0.82	0.93	0.56

External links

[dbSNP] [GenBank] [HaploReg] [RegulomeDB] [UCSC] [Ensemble]

**Variant:** 11 : 86014894, $C \rightarrow$T					
**Position:**	chr11:86014894	**Ref. allele:**	C	**Alt. allele:**	T
**Region:**	exonic	**Exonic. func.:**	synonymous	**Gene ID:**	PICALM
**RS number:**	rs592297	**Clin. sign.:**	-	-	

Results

**Tool:**	**PredictSNP2**	**CADD**	**DANN**	**FATHMM**	**FunSeq2**	**GWAVA**
**Prediction:**	neutral	neutral	neutral	neutral	neutral	neutral
**Score:**	-1.0000	12.9000	0.6510	0.4728	0.0000	0.3600
**Exp. accuracy:**	0.96	0.77	0.96	0.72	0.93	0.55

External links

[dbSNP] [GenBank] [HaploReg] [RegulomeDB] [UCSC] [Ensemble]

**Variant:** 11 : 86014923, $G \rightarrow$A					
**Position:**	chr11:86014923	**Ref. allele:**	G	**Alt. allele:**	A
**Region:**	exonic	**Exonic. func.:**	synonymous	**Gene ID:**	PICALM
**RS number:**	rs186932606	**Clin. sign.:**	-	-	

Results

**Tool:**	**PredictSNP2**	**CADD**	**DANN**	**FATHMM**	**FunSeq2**	**GWAVA**
**Prediction:**	neutral	neutral	neutral	deleterious	neutral	neutral
**Score:**	-0.5206	11.6400	0.6788	0.8999	0.0000	0.3500
**Exp. accuracy:**	0.93	0.83	0.94	0.71	0.93	0.54

External links

[dbSNP] [GenBank] [HaploReg] [RegulomeDB] [UCSC] [Ensemble]

**Variant:**	11 : 86014963, $C \to$G				
**Position:**	chr11:86014963	**Ref. allele:**	C	**Alt. allele:**	G
**Region:**	exonic	**Exonic. func.:**	synonymous	**Gene ID:**	PICALM
**RS number:**	-	**Clin. sign.:**	-	-	

Results

**Tool:**	**PredictSNP2**	**CADD**	**DANN**	**FATHMM**	**FunSeq2**	**GWAVA**
**Prediction:**	deleterious	neutral	deleterious	deleterious	deleterious	neutral
**Score:**	0.5498	15.5400	0.9477	0.9140	4.0000	0.3700
**Exp. accuracy:**	0.93	0.73	0.87	0.77	1.00	0.56

External links

[GenBank] [UCSC] [Ensemble]

**Variant:**	11 : 86022369, $T \to$C				
**Position:**	chr11:86022369	**Ref. allele:**	T	**Alt. allele:**	C
**Region:**	exonic	**Exonic. func.:**	synonymous	**Gene ID:**	PICALM
**RS number:**	-	**Clin. sign.:**	-	-	

Results

**Tool:**	**PredictSNP2**	**CADD**	**DANN**	**FATHMM**	**FunSeq2**	**GWAVA**
**Prediction:**	neutral	deleterious	neutral	deleterious	neutral	deleterious
**Score:**	-0.0534	15.7700	0.8477	0.9024	0.0000	0.5100
**Exp. accuracy:**	0.88	0.58	0.90	0.75	0.93	0.55

External links

[GenBank] [UCSC] [Ensemble]

**Variant:**	11 : 86022375, $C \to$T				
**Position:**	chr11:86022375	**Ref. allele:**	C	**Alt. allele:**	T
**Region:**	exonic	**Exonic. func.:**	synonymous	**Gene ID:**	PICALM
**RS number:**	-	**Clin. sign.:**	-	-	

Results

**Tool:**	**PredictSNP2**	**CADD**	**DANN**	**FATHMM**	**FunSeq2**	**GWAVA**
**Prediction:**	neutral	neutral	neutral	neutral	neutral	deleterious
**Score:**	-1.0000	13.4200	0.7679	0.6904	0.0000	0.5000
**Exp. accuracy:**	0.96	0.74	0.90	0.69	0.93	0.51

External links

[GenBank] [UCSC] [Ensemble]

**Variant:** 11 : 86022396, $A \to$C					
**Position:**	chr11:86022396	**Ref. allele:**	A	**Alt. allele:**	C
**Region:**	exonic	**Exonic. func.:**	synonymous	**Gene ID:**	PICALM
**RS number:**	-	**Clin. sign.:**	-	-	

Results

**Tool:**	**PredictSNP2**	**CADD**	**DANN**	**FATHMM**	**FunSeq2**	**GWAVA**
**Prediction:**	neutral	neutral	neutral	neutral	neutral	neutral
**Score:**	-1.0000	5.5420	0.7228	0.7505	0.0000	0.4200
**Exp. accuracy:**	0.96	0.92	0.93	0.69	0.93	0.54

External links

[GenBank] [UCSC] [Ensemble]

**Variant:** 11 : 86022453, $T \to$C					
**Position:**	chr11:86022453	**Ref. allele:**	T	**Alt. allele:**	C
**Region:**	exonic	**Exonic. func.:**	synonymous	**Gene ID:**	PICALM
**RS number:**	-	**Clin. sign.:**	-	-	

Results

**Tool:**	**PredictSNP2**	**CADD**	**DANN**	**FATHMM**	**FunSeq2**	**GWAVA**
**Prediction:**	neutral	neutral	neutral	neutral	deleterious	neutral
**Score:**	-0.3751	9.9620	0.8690	0.7566	3.0000	0.4200
**Exp. accuracy:**	0.88	0.91	0.90	0.69	1.00	0.54

External links

[GenBank] [UCSC] [Ensemble]

**Variant:** 11 : 86022465, $A \to$G					
**Position:**	chr11:86022465	**Ref. allele:**	A	**Alt. allele:**	G
**Region:**	exonic	**Exonic. func.:**	synonymous	**Gene ID:**	PICALM
**RS number:**	-	**Clin. sign.:**	-	-	

Results

**Tool:**	**PredictSNP2**	**CADD**	**DANN**	**FATHMM**	**FunSeq2**	**GWAVA**
**Prediction:**	deleterious	neutral	neutral	deleterious	deleterious	neutral
**Score:**	0.0917	8.8790	0.8691	0.9342	4.0000	0.4200
**Exp. accuracy:**	0.93	0.92	0.90	0.86	1.00	0.54

External links

[GenBank] [UCSC] [Ensemble]

**Variant:** 11 : 86022468, $T \rightarrow$C					
**Position:**	chr11:86022468	**Ref. allele:**	T	**Alt. allele:**	C
**Region:**	exonic	**Exonic. func.:**	synonymous	**Gene ID:**	PICALM
**RS number:**	-	**Clin. sign.:**	-	-	

Results

**Tool:**	**PredictSNP2**	**CADD**	**DANN**	**FATHMM**	**FunSeq2**	**GWAVA**
**Prediction:**	deleterious	neutral	deleterious	neutral	deleterious	neutral
**Score:**	0.1113	13.3200	0.9639	0.2183	3.0000	0.3200
**Exp. accuracy:**	0.93	0.74	0.87	0.77	1.00	0.53

External links

[GenBank] [UCSC] [Ensemble]

**Variant:** 11 : 86026296, $C \rightarrow$T					
**Position:**	chr11:86026296	**Ref. allele:**	C	**Alt. allele:**	T
**Region:**	exonic	**Exonic. func.:**	synonymous	**Gene ID:**	PICALM
**RS number:**	rs202207283	**Clin. sign.:**	-	-	

Results

**Tool:**	**PredictSNP2**	**CADD**	**DANN**	**FATHMM**	**FunSeq2**	**GWAVA**
**Prediction:**	neutral	neutral	neutral	neutral	neutral	neutral
**Score:**	-1.0000	14.1700	0.7042	0.7677	0.0000	0.3000
**Exp. accuracy:**	0.96	0.73	0.94	0.69	0.93	0.57

External links

[dbSNP] [GenBank] [HaploReg] [RegulomeDB] [UCSC] [Ensemble]

**Variant:** 11 : 86026323, $T \rightarrow$C					
**Position:**	chr11:86026323	**Ref. allele:**	T	**Alt. allele:**	C
**Region:**	exonic	**Exonic. func.:**	synonymous	**Gene ID:**	PICALM
**RS number:**	rs148486517	**Clin. sign.:**	-	-	

Results

**Tool:**	**PredictSNP2**	**CADD**	**DANN**	**FATHMM**	**FunSeq2**	**GWAVA**
**Prediction:**	neutral	neutral	neutral	deleterious	neutral	neutral
**Score:**	-0.5799	9.1020	0.7808	0.8600	0.0000	0.4600
**Exp. accuracy:**	0.93	0.92	0.90	0.57	0.93	0.54

External links

[dbSNP] [GenBank] [HaploReg] [RegulomeDB] [UCSC] [Ensemble]

**Variant:** 11 : 86026335, $C \to$T					
**Position:**	chr11:86026335	**Ref. allele:**	C	**Alt. allele:**	T
**Region:**	exonic	**Exonic. func.:**	synonymous	**Gene ID:**	PICALM
**RS number:**	-	**Clin. sign.:**	-	-	

Results

**Tool:**	**PredictSNP2**	**CADD**	**DANN**	**FATHMM**	**FunSeq2**	**GWAVA**
**Prediction:**	neutral	deleterious	neutral	neutral	neutral	neutral
**Score:**	-0.6391	16.8800	0.5891	0.0861	0.0000	0.3600
**Exp. accuracy:**	0.95	0.58	0.97	0.88	0.93	0.55

External links

[GenBank] [UCSC] [Ensemble]

**Variant:** 11 : 86031481, $C \to$T					
**Position:**	chr11:86031481	**Ref. allele:**	C	**Alt. allele:**	T
**Region:**	exonic	**Exonic. func.:**	synonymous	**Gene ID:**	PICALM
**RS number:**	-	**Clin. sign.:**	-	-	

Results

**Tool:**	**PredictSNP2**	**CADD**	**DANN**	**FATHMM**	**FunSeq2**	**GWAVA**
**Prediction:**	neutral	neutral	neutral	neutral	neutral	?
**Score:**	-1.0000	14.5300	0.7411	0.7343	0.0000	0.4800
**Exp. accuracy:**	0.96	0.73	0.91	0.69	0.93	0.43

External links

[GenBank] [UCSC] [Ensemble]

**Variant:** 11 : 86031523, $C \to$G					
**Position:**	chr11:86031523	**Ref. allele:**	C	**Alt. allele:**	G
**Region:**	exonic	**Exonic. func.:**	synonymous	**Gene ID:**	PICALM
**RS number:**	-	**Clin. sign.:**	-	-	

Results

**Tool:**	**PredictSNP2**	**CADD**	**DANN**	**FATHMM**	**FunSeq2**	**GWAVA**
**Prediction:**	neutral	neutral	neutral	deleterious	neutral	?
**Score:**	-0.5575	12.3300	0.6918	0.8695	0.0000	0.4900
**Exp. accuracy:**	0.93	0.83	0.94	0.60	0.93	0.43

External links

[GenBank] [UCSC] [Ensemble]

**Variant:** 11 : 86031523, $C \rightarrow$T					
**Position:**	chr11:86031523	**Ref. allele:**	C	**Alt. allele:**	T
**Region:**	exonic	**Exonic. func.:**	synonymous	**Gene ID:**	PICALM
**RS number:**	rs140266798	**Clin. sign.:**	-	-	

Results

**Tool:**	**PredictSNP2**	**CADD**	**DANN**	**FATHMM**	**FunSeq2**	**GWAVA**
**Prediction:**	neutral	neutral	neutral	neutral	neutral	?
**Score:**	-1.0000	13.6500	0.6654	0.7859	0.0000	0.4900
**Exp. accuracy:**	0.96	0.73	0.95	0.69	0.93	0.43

External links

[dbSNP] [GenBank] [HaploReg] [RegulomeDB] [UCSC] [Ensemble]

**Variant:** 11 : 86031559, $G \rightarrow$A					
**Position:**	chr11:86031559	**Ref. allele:**	G	**Alt. allele:**	A
**Region:**	exonic	**Exonic. func.:**	synonymous	**Gene ID:**	PICALM
**RS number:**	rs142062212	**Clin. sign.:**	-	-	

Results

**Tool:**	**PredictSNP2**	**CADD**	**DANN**	**FATHMM**	**FunSeq2**	**GWAVA**
**Prediction:**	neutral	neutral	neutral	deleterious	neutral	deleterious
**Score:**	-0.4756	10.9000	0.6150	0.9470	0.0000	0.5500
**Exp. accuracy:**	0.93	0.88	0.96	0.91	0.93	0.66

External links

[dbSNP] [GenBank] [HaploReg] [RegulomeDB] [UCSC] [Ensemble]

**Variant:** 11 : 86031571, $T \rightarrow$C					
**Position:**	chr11:86031571	**Ref. allele:**	T	**Alt. allele:**	C
**Region:**	exonic	**Exonic. func.:**	synonymous	**Gene ID:**	PICALM
**RS number:**	rs151105998	**Clin. sign.:**	-	-	

Results

**Tool:**	**PredictSNP2**	**CADD**	**DANN**	**FATHMM**	**FunSeq2**	**GWAVA**
**Prediction:**	neutral	neutral	neutral	neutral	neutral	deleterious
**Score:**	-1.0000	9.3380	0.6938	0.2327	0.0000	0.5200
**Exp. accuracy:**	0.96	0.91	0.94	0.73	0.93	0.58

External links

[dbSNP] [GenBank] [HaploReg] [RegulomeDB] [UCSC] [Ensemble]

**Variant:** 11 : 86031577, $G \rightarrow$A					
**Position:**	chr11:86031577	**Ref. allele:**	G	**Alt. allele:**	A
**Region:**	exonic	**Exonic. func.:**	synonymous	**Gene ID:**	PICALM
**RS number:**	-	**Clin. sign.:**	-	-	

Results

**Tool:**	**PredictSNP2**	**CADD**	**DANN**	**FATHMM**	**FunSeq2**	**GWAVA**
**Prediction:**	neutral	neutral	neutral	deleterious	neutral	deleterious
**Score:**	-0.4845	11.5400	0.6322	0.9321	0.0000	0.5300
**Exp. accuracy:**	0.93	0.88	0.97	0.86	0.93	0.64

External links

[GenBank] [UCSC] [Ensemble]

**Variant:** 11 : 86068657, $G \rightarrow$A					
**Position:**	chr11:86068657	**Ref. allele:**	G	**Alt. allele:**	A
**Region:**	exonic	**Exonic. func.:**	synonymous	**Gene ID:**	PICALM
**RS number:**	-	**Clin. sign.:**	-	-	

Results

**Tool:**	**PredictSNP2**	**CADD**	**DANN**	**FATHMM**	**FunSeq2**	**GWAVA**
**Prediction:**	deleterious	deleterious	deleterious	deleterious	neutral	neutral
**Score:**	0.4592	17.4700	0.9650	0.7963	0.0000	0.3400
**Exp. accuracy:**	0.93	0.58	0.87	0.57	0.93	0.56

External links

[GenBank] [UCSC] [Ensemble]

**Variant:** 11 : 86068661, $C \rightarrow$T					
**Position:**	chr11:86068661	**Ref. allele:**	C	**Alt. allele:**	T
**Region:**	exonic	**Exonic. func.:**	synonymous	**Gene ID:**	PICALM
**RS number:**	rs150283404	**Clin. sign.:**	-	-	

Results

**Tool:**	**PredictSNP2**	**CADD**	**DANN**	**FATHMM**	**FunSeq2**	**GWAVA**
**Prediction:**	neutral	deleterious	deleterious	neutral	neutral	neutral
**Score:**	0.0072	16.6800	0.9680	0.6263	0.0000	0.3100
**Exp. accuracy:**	0.88	0.58	0.87	0.69	0.93	0.53

External links

[dbSNP] [GenBank] [HaploReg] [RegulomeDB] [UCSC] [Ensemble]

**Variant:** 11 : 86068673, $C \rightarrow$T					
**Position:**	chr11:86068673	**Ref. allele:**	C	**Alt. allele:**	T
**Region:**	exonic	**Exonic. func.:**	synonymous	**Gene ID:**	PICALM
**RS number:**	-	**Clin. sign.:**	-	-	

Results

**Tool:**	**PredictSNP2**	**CADD**	**DANN**	**FATHMM**	**FunSeq2**	**GWAVA**
**Prediction:**	neutral	deleterious	deleterious	neutral	neutral	neutral
**Score:**	-0.0254	17.3200	0.9581	0.6314	0.0000	0.2700
**Exp. accuracy:**	0.88	0.58	0.87	0.69	0.93	0.55

External links

[GenBank] [UCSC] [Ensemble]

**Variant:** 11 : 86068703, $T \rightarrow$C					
**Position:**	chr11:86068703	**Ref. allele:**	T	**Alt. allele:**	C
**Region:**	exonic	**Exonic. func.:**	synonymous	**Gene ID:**	PICALM
**RS number:**	-	**Clin. sign.:**	-	-	

Results

**Tool:**	**PredictSNP2**	**CADD**	**DANN**	**FATHMM**	**FunSeq2**	**GWAVA**
**Prediction:**	neutral	deleterious	neutral	neutral	neutral	neutral
**Score:**	-0.5941	17.4900	0.7616	0.5796	0.0000	0.4700
**Exp. accuracy:**	0.93	0.58	0.90	0.70	0.93	0.54

External links

[GenBank] [UCSC] [Ensemble]

**Variant:** 11 : 86068715, $T \rightarrow$C					
**Position:**	chr11:86068715	**Ref. allele:**	T	**Alt. allele:**	C
**Region:**	exonic	**Exonic. func.:**	synonymous	**Gene ID:**	PICALM
**RS number:**	rs200624220	**Clin. sign.:**	-	-	

Results

**Tool:**	**PredictSNP2**	**CADD**	**DANN**	**FATHMM**	**FunSeq2**	**GWAVA**
**Prediction:**	neutral	neutral	neutral	neutral	neutral	neutral
**Score:**	-1.0000	5.2440	0.4790	0.0365	0.0000	0.3800
**Exp. accuracy:**	0.96	0.92	0.97	0.96	0.93	0.55

External links

[dbSNP] [GenBank] [HaploReg] [RegulomeDB] [UCSC] [Ensemble]

**Variant:** 11 : 86068724, $G{\to}$C					
**Position:**	chr11:86068724	**Ref. allele:**	G	**Alt. allele:**	C
**Region:**	exonic	**Exonic. func.:**	synonymous	**Gene ID:**	PICALM
**RS number:**	-	**Clin. sign.:**	-	-	

Results

**Tool:**	**PredictSNP2**	**CADD**	**DANN**	**FATHMM**	**FunSeq2**	**GWAVA**
**Prediction:**	neutral	neutral	neutral	deleterious	neutral	?
**Score:**	-0.5947	11.1400	0.5060	0.8078	0.0000	0.4900
**Exp. accuracy:**	0.93	0.88	0.97	0.57	0.93	0.43

External links

[GenBank] [UCSC] [Ensemble]

**Variant:** 11 : 86068727, $G{\to}$T					
**Position:**	chr11:86068727	**Ref. allele:**	G	**Alt. allele:**	T
**Region:**	exonic	**Exonic. func.:**	synonymous	**Gene ID:**	PICALM
**RS number:**	-	**Clin. sign.:**	-	-	

Results

**Tool:**	**PredictSNP2**	**CADD**	**DANN**	**FATHMM**	**FunSeq2**	**GWAVA**
**Prediction:**	neutral	neutral	neutral	neutral	neutral	neutral
**Score:**	-1.0000	9.3130	0.4774	0.7482	0.0000	0.4000
**Exp. accuracy:**	0.96	0.91	0.97	0.69	0.93	0.56

External links

[GenBank] [UCSC] [Ensemble]

**Variant:** 11 : 86068754, $T{\to}$C					
**Position:**	chr11:86068754	**Ref. allele:**	T	**Alt. allele:**	C
**Region:**	exonic	**Exonic. func.:**	synonymous	**Gene ID:**	PICALM
**RS number:**	-	**Clin. sign.:**	-	-	

Results

**Tool:**	**PredictSNP2**	**CADD**	**DANN**	**FATHMM**	**FunSeq2**	**GWAVA**
**Prediction:**	neutral	deleterious	neutral	neutral	neutral	neutral
**Score:**	-0.5989	16.3400	0.7207	0.4389	0.0000	0.2600
**Exp. accuracy:**	0.93	0.58	0.93	0.72	0.93	0.54

External links

[GenBank] [UCSC] [Ensemble]

**Variant:** $11 : 86068757, G{\rightarrow}$A					
**Position:**	chr11:86068757	**Ref. allele:**	G	**Alt. allele:**	A
**Region:**	exonic	**Exonic. func.:**	synonymous	**Gene ID:**	PICALM
**RS number:**	rs367839126	**Clin. sign.:**	-	-	

Results

**Tool:**	**PredictSNP2**	**CADD**	**DANN**	**FATHMM**	**FunSeq2**	**GWAVA**
**Prediction:**	deleterious	deleterious	deleterious	deleterious	neutral	neutral
**Score:**	0.4858	19.8100	0.9597	0.8890	0.0000	0.4100
**Exp. accuracy:**	0.93	0.69	0.87	0.68	0.93	0.56

External links

[dbSNP] [GenBank] [RegulomeDB] [UCSC] [Ensemble]
