# Supplementary material for: Computational analysis of functional SNPs in Alzheimer’s disease-associated endocytosis genes
Source: PeerJ. 2019 Sep 30;7:e7667. doi: 10.7717/peerj.7667 (PMC6776068; doi:10.7717/peerj.7667)
Supplement: Table S6 — The result file was generated by PredictSNP2. [file peerj-07-7667-s010.pdf]

PredictSNP overview

Date: 2017/03/06
Genome version: GRCh38/hg38
PredictSNP version: 2.1

If you use PredictSNP and obtain scientific results that you publish, we would ask you to acknowledge the usage of PredictSNP by referencing the article below.

Bendl, J., Musil, M., Stourac, J., Zendulka, J., Damborsky, J., Brezovsky, J., 2016: PredictSNP2: A unified platform for accurately evaluating SNP effects by exploiting the different characteristics of variants in distinct genomic regions. *PLOS Computational Biology* 12: e1004962

	PredictSNP2		CADD		DANN		FATHMM		FunSeq2		GWAVA	
	Neu	Del	Neu	Del	Neu	Del	Neu	Del	Neu	Del	Neu	Del
Regulatory	0	0	0	0	0	0	0	0	0	0	0	0
Splicing	0	0	0	0	0	0	0	0	0	0	0	0
Missense	0	0	0	0	0	0	96	0	93	3	94	2
Synonymous	74	22	96	0	43	49	0	0	0	0	0	0
Nonsense	0	0	0	0	0	0	0	0	0	0	0	0
Others	0	0	0	0	0	0	0	0	0	0	0	0

**Variant:** 21 : 32634876, $T \to$C					
**Position:**	chr21:32634876	**Ref. allele:**	T	**Alt. allele:**	C
**Region:**	exonic	**Exonic. func.:**	synonymous	**Gene ID:**	SYNJ1
**RS number:**	-	**Clin. sign.:**	-	-	

Results

**Tool:**	**PredictSNP2**	**CADD**	**DANN**	**FATHMM**	**FunSeq2**	**GWAVA**
**Prediction:**	neutral	deleterious	neutral	deleterious	neutral	deleterious
**Score:**	-0.0974	19.5900	0.6048	0.8948	0.0000	0.6500
**Exp. accuracy:**	0.88	0.69	0.96	0.71	0.93	0.70

External links

[GenBank] [UCSC] [Ensemble]

**Variant:** 21 : 32638911, $C \to$A					
**Position:**	chr21:32638911	**Ref. allele:**	C	**Alt. allele:**	A
**Region:**	exonic	**Exonic. func.:**	synonymous	**Gene ID:**	SYNJ1
**RS number:**	rs372695378	**Clin. sign.:**	-	-	

Results

**Tool:**	**PredictSNP2**	**CADD**	**DANN**	**FATHMM**	**FunSeq2**	**GWAVA**
**Prediction:**	neutral	neutral	neutral	neutral	neutral	?
**Score:**	-1.0000	11.0800	0.3700	0.6560	0.0000	0.4900
**Exp. accuracy:**	0.96	0.88	0.97	0.69	0.93	0.43

External links

[dbSNP] [GenBank] [RegulomeDB] [UCSC] [Ensemble]

**Variant:** 21 : 32638911, $C \to$T					
**Position:**	chr21:32638911	**Ref. allele:**	C	**Alt. allele:**	T
**Region:**	exonic	**Exonic. func.:**	synonymous	**Gene ID:**	SYNJ1
**RS number:**	-	**Clin. sign.:**	-	-	

Results

**Tool:**	**PredictSNP2**	**CADD**	**DANN**	**FATHMM**	**FunSeq2**	**GWAVA**
**Prediction:**	neutral	neutral	neutral	neutral	neutral	?
**Score:**	-1.0000	10.5800	0.3930	0.3106	0.0000	0.4900
**Exp. accuracy:**	0.96	0.88	0.97	0.73	0.93	0.43

External links

[GenBank] [UCSC] [Ensemble]

**Variant:** 21 : 32638947, $T \rightarrow$A					
**Position:**	chr21:32638947	**Ref. allele:**	T	**Alt. allele:**	A
**Region:**	exonic	**Exonic. func.:**	synonymous	**Gene ID:**	SYNJ1
**RS number:**	-	**Clin. sign.:**	-	-	

Results

**Tool:**	**PredictSNP2**	**CADD**	**DANN**	**FATHMM**	**FunSeq2**	**GWAVA**
**Prediction:**	neutral	neutral	neutral	neutral	neutral	deleterious
**Score:**	-1.0000	9.8270	0.6017	0.0519	0.0000	0.5100
**Exp. accuracy:**	0.96	0.91	0.96	0.94	0.93	0.55

External links

[GenBank] [UCSC] [Ensemble]

**Variant:** 21 : 32638950, $C \rightarrow$T					
**Position:**	chr21:32638950	**Ref. allele:**	C	**Alt. allele:**	T
**Region:**	exonic	**Exonic. func.:**	synonymous	**Gene ID:**	SYNJ1
**RS number:**	-	**Clin. sign.:**	-	-	

Results

**Tool:**	**PredictSNP2**	**CADD**	**DANN**	**FATHMM**	**FunSeq2**	**GWAVA**
**Prediction:**	neutral	deleterious	neutral	neutral	neutral	deleterious
**Score:**	-0.6000	15.6700	0.7138	0.7348	0.0000	0.6800
**Exp. accuracy:**	0.95	0.58	0.93	0.69	0.93	0.70

External links

[GenBank] [UCSC] [Ensemble]

**Variant:** 21 : 32638989, $T \rightarrow$A					
**Position:**	chr21:32638989	**Ref. allele:**	T	**Alt. allele:**	A
**Region:**	exonic	**Exonic. func.:**	synonymous	**Gene ID:**	SYNJ1
**RS number:**	-	**Clin. sign.:**	-	-	

Results

**Tool:**	**PredictSNP2**	**CADD**	**DANN**	**FATHMM**	**FunSeq2**	**GWAVA**
**Prediction:**	neutral	neutral	neutral	neutral	neutral	deleterious
**Score:**	-1.0000	1.0220	0.5861	0.0407	0.0000	0.5200
**Exp. accuracy:**	0.96	0.95	0.97	0.96	0.93	0.58

External links

[GenBank] [UCSC] [Ensemble]

Variant:	21 : 32639016, $T \rightarrow$G				
**Position:**	chr21:32639016	**Ref. allele:**	T	**Alt. allele:**	G
**Region:**	exonic	**Exonic. func.:**	synonymous	**Gene ID:**	SYNJ1
**RS number:**	-	**Clin. sign.:**	-	-	

Results

**Tool:**	**PredictSNP2**	**CADD**	**DANN**	**FATHMM**	**FunSeq2**	**GWAVA**
**Prediction:**	neutral	neutral	neutral	neutral	neutral	deleterious
**Score:**	-1.0000	2.4550	0.5013	0.0443	0.0000	0.5300
**Exp. accuracy:**	0.96	0.95	0.97	0.96	0.93	0.64

External links

[GenBank] [UCSC] [Ensemble]

Variant:	21 : 32639034, $A \rightarrow$C				
**Position:**	chr21:32639034	**Ref. allele:**	A	**Alt. allele:**	C
**Region:**	exonic	**Exonic. func.:**	synonymous	**Gene ID:**	SYNJ1
**RS number:**	-	**Clin. sign.:**	-	-	

Results

**Tool:**	**PredictSNP2**	**CADD**	**DANN**	**FATHMM**	**FunSeq2**	**GWAVA**
**Prediction:**	neutral	neutral	neutral	neutral	neutral	deleterious
**Score:**	-1.0000	0.0030	0.5101	0.2148	0.0000	0.5500
**Exp. accuracy:**	0.96	0.95	0.97	0.77	0.93	0.66

External links

[GenBank] [UCSC] [Ensemble]

Variant:	21 : 32639037, $C \rightarrow$T				
**Position:**	chr21:32639037	**Ref. allele:**	C	**Alt. allele:**	T
**Region:**	exonic	**Exonic. func.:**	synonymous	**Gene ID:**	SYNJ1
**RS number:**	-	**Clin. sign.:**	-	-	

Results

**Tool:**	**PredictSNP2**	**CADD**	**DANN**	**FATHMM**	**FunSeq2**	**GWAVA**
**Prediction:**	neutral	neutral	neutral	neutral	neutral	deleterious
**Score:**	-1.0000	12.1300	0.5678	0.6645	0.0000	0.7300
**Exp. accuracy:**	0.96	0.83	0.97	0.69	0.93	0.70

External links

[GenBank] [UCSC] [Ensemble]

**Variant:** 21 : 32639058, $C \rightarrow$T					
**Position:**	chr21:32639058	**Ref. allele:**	C	**Alt. allele:**	T
**Region:**	exonic	**Exonic. func.:**	synonymous	**Gene ID:**	SYNJ1
**RS number:**	-	**Clin. sign.:**	-	-	

Results

**Tool:**	**PredictSNP2**	**CADD**	**DANN**	**FATHMM**	**FunSeq2**	**GWAVA**
**Prediction:**	neutral	neutral	neutral	neutral	neutral	deleterious
**Score:**	-1.0000	8.5430	0.4633	0.0083	0.0000	0.5700
**Exp. accuracy:**	0.96	0.92	0.97	0.96	0.93	0.68

External links

[GenBank] [UCSC] [Ensemble]

**Variant:** 21 : 32639061, $G \rightarrow$A					
**Position:**	chr21:32639061	**Ref. allele:**	G	**Alt. allele:**	A
**Region:**	exonic	**Exonic. func.:**	synonymous	**Gene ID:**	SYNJ1
**RS number:**	rs201117151	**Clin. sign.:**	-	-	

Results

**Tool:**	**PredictSNP2**	**CADD**	**DANN**	**FATHMM**	**FunSeq2**	**GWAVA**
**Prediction:**	neutral	neutral	neutral	neutral	neutral	deleterious
**Score:**	-1.0000	0.2980	0.4000	0.0613	0.0000	0.5700
**Exp. accuracy:**	0.96	0.95	0.97	0.92	0.93	0.68

External links

[dbSNP] [GenBank] [HaploReg] [RegulomeDB] [UCSC] [Ensemble]

**Variant:** 21 : 32639070, $A \rightarrow$C					
**Position:**	chr21:32639070	**Ref. allele:**	A	**Alt. allele:**	C
**Region:**	exonic	**Exonic. func.:**	synonymous	**Gene ID:**	SYNJ1
**RS number:**	rs150595970	**Clin. sign.:**	-	-	

Results

**Tool:**	**PredictSNP2**	**CADD**	**DANN**	**FATHMM**	**FunSeq2**	**GWAVA**
**Prediction:**	neutral	neutral	neutral	neutral	neutral	deleterious
**Score:**	-1.0000	0.0020	0.5074	0.0572	0.0000	0.5600
**Exp. accuracy:**	0.96	0.95	0.97	0.92	0.93	0.69

External links

[dbSNP] [GenBank] [HaploReg] [RegulomeDB] [UCSC] [Ensemble]

**Variant:**	21 : 32639076, $C \rightarrow$ A				
**Position:**	chr21:32639076	**Ref. allele:**	C	**Alt. allele:**	A
**Region:**	exonic	**Exonic. func.:**	synonymous	**Gene ID:**	SYNJ1
**RS number:**	rs138579227	**Clin. sign.:**	-	-	

Results

**Tool:**	**PredictSNP2**	**CADD**	**DANN**	**FATHMM**	**FunSeq2**	**GWAVA**
**Prediction:**	neutral	neutral	neutral	neutral	neutral	?
**Score:**	-1.0000	8.8030	0.3735	0.0334	0.0000	0.4900
**Exp. accuracy:**	0.96	0.92	0.97	0.96	0.93	0.43

External links

[dbSNP] [GenBank] [HaploReg] [RegulomeDB] [UCSC] [Ensemble]

**Variant:**	21 : 32639076, $C \rightarrow$ T				
**Position:**	chr21:32639076	**Ref. allele:**	C	**Alt. allele:**	T
**Region:**	exonic	**Exonic. func.:**	synonymous	**Gene ID:**	SYNJ1
**RS number:**	-	**Clin. sign.:**	-	-	

Results

**Tool:**	**PredictSNP2**	**CADD**	**DANN**	**FATHMM**	**FunSeq2**	**GWAVA**
**Prediction:**	neutral	neutral	neutral	neutral	neutral	?
**Score:**	-1.0000	7.8260	0.4095	0.0391	0.0000	0.4900
**Exp. accuracy:**	0.96	0.91	0.97	0.96	0.93	0.43

External links

[GenBank] [UCSC] [Ensemble]

**Variant:**	21 : 32639091, $C \rightarrow$ T				
**Position:**	chr21:32639091	**Ref. allele:**	C	**Alt. allele:**	T
**Region:**	exonic	**Exonic. func.:**	synonymous	**Gene ID:**	SYNJ1
**RS number:**	-	**Clin. sign.:**	-	-	

Results

**Tool:**	**PredictSNP2**	**CADD**	**DANN**	**FATHMM**	**FunSeq2**	**GWAVA**
**Prediction:**	neutral	neutral	neutral	deleterious	neutral	deleterious
**Score:**	-0.4698	11.3300	0.6755	0.9441	0.0000	0.7300
**Exp. accuracy:**	0.93	0.88	0.94	0.90	0.93	0.70

External links

[GenBank] [UCSC] [Ensemble]

**Variant:** 21 : 32639102, $G \to$A					
**Position:**	chr21:32639102	**Ref. allele:**	G	**Alt. allele:**	A
**Region:**	exonic	**Exonic. func.:**	synonymous	**Gene ID:**	SYNJ1
**RS number:**	rs2230766	**Clin. sign.:**	-	-	

Results

**Tool:**	**PredictSNP2**	**CADD**	**DANN**	**FATHMM**	**FunSeq2**	**GWAVA**
**Prediction:**	neutral	neutral	neutral	deleterious	neutral	deleterious
**Score:**	-0.5849	7.4630	0.6621	0.8221	0.0000	0.6200
**Exp. accuracy:**	0.93	0.91	0.95	0.57	0.93	0.66

External links

[dbSNP] [GenBank] [HaploReg] [RegulomeDB] [UCSC] [Ensemble]

**Variant:** 21 : 32639675, $C \to$T					
**Position:**	chr21:32639675	**Ref. allele:**	C	**Alt. allele:**	T
**Region:**	exonic	**Exonic. func.:**	synonymous	**Gene ID:**	SYNJ1
**RS number:**	-	**Clin. sign.:**	-	-	

Results

**Tool:**	**PredictSNP2**	**CADD**	**DANN**	**FATHMM**	**FunSeq2**	**GWAVA**
**Prediction:**	neutral	neutral	neutral	neutral	neutral	deleterious
**Score:**	-1.0000	7.2500	0.5045	0.0228	0.0000	0.5700
**Exp. accuracy:**	0.96	0.91	0.97	0.96	0.93	0.68

External links

[GenBank] [UCSC] [Ensemble]

**Variant:** 21 : 32639684, $T \to$C					
**Position:**	chr21:32639684	**Ref. allele:**	T	**Alt. allele:**	C
**Region:**	exonic	**Exonic. func.:**	synonymous	**Gene ID:**	SYNJ1
**RS number:**	-	**Clin. sign.:**	-	-	

Results

**Tool:**	**PredictSNP2**	**CADD**	**DANN**	**FATHMM**	**FunSeq2**	**GWAVA**
**Prediction:**	neutral	neutral	neutral	neutral	neutral	deleterious
**Score:**	-1.0000	0.0280	0.3747	0.0275	0.0000	0.5400
**Exp. accuracy:**	0.96	0.95	0.97	0.96	0.93	0.62

External links

[GenBank] [UCSC] [Ensemble]

**Variant:** 21 : 32639708, $A \rightarrow$G					
**Position:**	chr21:32639708	**Ref. allele:**	A	**Alt. allele:**	G
**Region:**	exonic	**Exonic. func.:**	synonymous	**Gene ID:**	SYNJ1
**RS number:**	-	**Clin. sign.:**	-	-	

Results

**Tool:**	**PredictSNP2**	**CADD**	**DANN**	**FATHMM**	**FunSeq2**	**GWAVA**
**Prediction:**	neutral	neutral	neutral	neutral	neutral	deleterious
**Score:**	-1.0000	6.2540	0.5898	0.1492	0.0000	0.5100
**Exp. accuracy:**	0.96	0.93	0.97	0.83	0.93	0.55

External links

[GenBank] [UCSC] [Ensemble]

**Variant:** 21 : 32639729, $C \rightarrow$T					
**Position:**	chr21:32639729	**Ref. allele:**	C	**Alt. allele:**	T
**Region:**	exonic	**Exonic. func.:**	synonymous	**Gene ID:**	SYNJ1
**RS number:**	-	**Clin. sign.:**	-	-	

Results

**Tool:**	**PredictSNP2**	**CADD**	**DANN**	**FATHMM**	**FunSeq2**	**GWAVA**
**Prediction:**	neutral	neutral	neutral	neutral	neutral	deleterious
**Score:**	-1.0000	12.9300	0.5706	0.6424	0.0000	0.5300
**Exp. accuracy:**	0.96	0.77	0.97	0.69	0.93	0.64

External links

[GenBank] [UCSC] [Ensemble]

**Variant:** 21 : 32639732, $C \rightarrow$T					
**Position:**	chr21:32639732	**Ref. allele:**	C	**Alt. allele:**	T
**Region:**	exonic	**Exonic. func.:**	synonymous	**Gene ID:**	SYNJ1
**RS number:**	-	**Clin. sign.:**	-	-	

Results

**Tool:**	**PredictSNP2**	**CADD**	**DANN**	**FATHMM**	**FunSeq2**	**GWAVA**
**Prediction:**	neutral	neutral	neutral	neutral	neutral	deleterious
**Score:**	-1.0000	12.0900	0.7299	0.5451	0.0000	0.5400
**Exp. accuracy:**	0.96	0.83	0.91	0.70	0.93	0.62

External links

[GenBank] [UCSC] [Ensemble]

**Variant:** 21 : 32639735, $G \to$A					
**Position:**	chr21:32639735	**Ref. allele:**	G	**Alt. allele:**	A
**Region:**	exonic	**Exonic. func.:**	synonymous	**Gene ID:**	SYNJ1
**RS number:**	-	**Clin. sign.:**	-	-	

Results

**Tool:**	**PredictSNP2**	**CADD**	**DANN**	**FATHMM**	**FunSeq2**	**GWAVA**
**Prediction:**	neutral	neutral	neutral	neutral	neutral	deleterious
**Score:**	-1.0000	1.3590	0.4995	0.0180	0.0000	0.5400
**Exp. accuracy:**	0.96	0.95	0.97	0.96	0.93	0.62

External links

[GenBank] [UCSC] [Ensemble]

**Variant:** 21 : 32639777, $C \to$T					
**Position:**	chr21:32639777	**Ref. allele:**	C	**Alt. allele:**	T
**Region:**	exonic	**Exonic. func.:**	synonymous	**Gene ID:**	SYNJ1
**RS number:**	-	**Clin. sign.:**	-	-	

Results

**Tool:**	**PredictSNP2**	**CADD**	**DANN**	**FATHMM**	**FunSeq2**	**GWAVA**
**Prediction:**	neutral	neutral	neutral	neutral	neutral	deleterious
**Score:**	-1.0000	0.0180	0.5145	0.0413	0.0000	0.5500
**Exp. accuracy:**	0.96	0.95	0.97	0.96	0.93	0.66

External links

[GenBank] [UCSC] [Ensemble]

**Variant:** 21 : 32641902, $G \to$A					
**Position:**	chr21:32641902	**Ref. allele:**	G	**Alt. allele:**	A
**Region:**	exonic	**Exonic. func.:**	synonymous	**Gene ID:**	SYNJ1
**RS number:**	-	**Clin. sign.:**	-	-	

Results

**Tool:**	**PredictSNP2**	**CADD**	**DANN**	**FATHMM**	**FunSeq2**	**GWAVA**
**Prediction:**	neutral	neutral	neutral	neutral	neutral	neutral
**Score:**	-1.0000	10.3700	0.7078	0.7077	0.0000	0.3300
**Exp. accuracy:**	0.96	0.88	0.93	0.69	0.93	0.56

External links

[GenBank] [UCSC] [Ensemble]

**Variant:** $21:32641905, T{\to}$C					
**Position:**	chr21:32641905	**Ref. allele:**	T	**Alt. allele:**	C
**Region:**	exonic	**Exonic. func.:**	synonymous	**Gene ID:**	SYNJ1
**RS number:**	-	**Clin. sign.:**	-	-	

Results

**Tool:**	**PredictSNP2**	**CADD**	**DANN**	**FATHMM**	**FunSeq2**	**GWAVA**
**Prediction:**	neutral	neutral	neutral	neutral	neutral	neutral
**Score:**	-1.0000	1.9360	0.5330	0.0543	0.0000	0.2600
**Exp. accuracy:**	0.96	0.95	0.97	0.94	0.93	0.54

External links

[GenBank] [UCSC] [Ensemble]

**Variant:** $21:32641926, T{\to}$C					
**Position:**	chr21:32641926	**Ref. allele:**	T	**Alt. allele:**	C
**Region:**	exonic	**Exonic. func.:**	synonymous	**Gene ID:**	SYNJ1
**RS number:**	-	**Clin. sign.:**	-	-	

Results

**Tool:**	**PredictSNP2**	**CADD**	**DANN**	**FATHMM**	**FunSeq2**	**GWAVA**
**Prediction:**	neutral	neutral	neutral	neutral	neutral	neutral
**Score:**	-1.0000	6.6930	0.6679	0.7505	0.0000	0.2400
**Exp. accuracy:**	0.96	0.92	0.95	0.69	0.93	0.58

External links

[GenBank] [UCSC] [Ensemble]

**Variant:** $21:32641938, T{\to}$A					
**Position:**	chr21:32641938	**Ref. allele:**	T	**Alt. allele:**	A
**Region:**	exonic	**Exonic. func.:**	synonymous	**Gene ID:**	SYNJ1
**RS number:**	-	**Clin. sign.:**	-	-	

Results

**Tool:**	**PredictSNP2**	**CADD**	**DANN**	**FATHMM**	**FunSeq2**	**GWAVA**
**Prediction:**	neutral	neutral	neutral	deleterious	neutral	neutral
**Score:**	-0.4236	13.2000	0.7736	0.9583	0.0000	0.3700
**Exp. accuracy:**	0.88	0.74	0.90	0.93	0.93	0.56

External links

[GenBank] [UCSC] [Ensemble]

**Variant:** 21 : 32643453, $A \rightarrow$G					
**Position:**	chr21:32643453	**Ref. allele:**	A	**Alt. allele:**	G
**Region:**	exonic	**Exonic. func.:**	synonymous	**Gene ID:**	SYNJ1
**RS number:**	-	**Clin. sign.:**	-	-	

Results

**Tool:**	**PredictSNP2**	**CADD**	**DANN**	**FATHMM**	**FunSeq2**	**GWAVA**
**Prediction:**	neutral	neutral	neutral	neutral	neutral	deleterious
**Score:**	-1.0000	0.5670	0.6900	0.3045	0.0000	0.5000
**Exp. accuracy:**	0.96	0.95	0.94	0.73	0.93	0.51

External links

[GenBank] [UCSC] [Ensemble]

**Variant:** 21 : 32645653, $C \rightarrow$T					
**Position:**	chr21:32645653	**Ref. allele:**	C	**Alt. allele:**	T
**Region:**	exonic	**Exonic. func.:**	synonymous	**Gene ID:**	SYNJ1
**RS number:**	-	**Clin. sign.:**	-	-	

Results

**Tool:**	**PredictSNP2**	**CADD**	**DANN**	**FATHMM**	**FunSeq2**	**GWAVA**
**Prediction:**	neutral	neutral	deleterious	neutral	neutral	neutral
**Score:**	-0.5383	13.9600	0.9226	0.0365	0.0000	0.2300
**Exp. accuracy:**	0.93	0.73	0.87	0.96	0.93	0.59

External links

[GenBank] [UCSC] [Ensemble]

**Variant:** 21 : 32645659, $A \rightarrow$T					
**Position:**	chr21:32645659	**Ref. allele:**	A	**Alt. allele:**	T
**Region:**	exonic	**Exonic. func.:**	synonymous	**Gene ID:**	SYNJ1
**RS number:**	rs376132504	**Clin. sign.:**	-	-	

Results

**Tool:**	**PredictSNP2**	**CADD**	**DANN**	**FATHMM**	**FunSeq2**	**GWAVA**
**Prediction:**	neutral	neutral	neutral	neutral	neutral	neutral
**Score:**	-1.0000	14.1000	0.7860	0.1405	0.0000	0.2200
**Exp. accuracy:**	0.96	0.73	0.90	0.85	0.93	0.59

External links

[dbSNP] [GenBank] [RegulomeDB] [UCSC] [Ensemble]

**Variant:** 21 : 32645668, $T \rightarrow$C					
**Position:**	chr21:32645668	**Ref. allele:**	T	**Alt. allele:**	C
**Region:**	exonic	**Exonic. func.:**	synonymous	**Gene ID:**	SYNJ1
**RS number:**	rs368850131	**Clin. sign.:**	-	-	

Results

**Tool:**	**PredictSNP2**	**CADD**	**DANN**	**FATHMM**	**FunSeq2**	**GWAVA**
**Prediction:**	neutral	neutral	neutral	neutral	neutral	neutral
**Score:**	-1.0000	14.9100	0.5617	0.6249	0.0000	0.2000
**Exp. accuracy:**	0.96	0.73	0.97	0.69	0.93	0.60

External links

[dbSNP] [GenBank] [RegulomeDB] [UCSC] [Ensemble]

**Variant:** 21 : 32645704, $G \rightarrow$C					
**Position:**	chr21:32645704	**Ref. allele:**	G	**Alt. allele:**	C
**Region:**	exonic	**Exonic. func.:**	synonymous	**Gene ID:**	SYNJ1
**RS number:**	rs372510070	**Clin. sign.:**	-	-	

Results

**Tool:**	**PredictSNP2**	**CADD**	**DANN**	**FATHMM**	**FunSeq2**	**GWAVA**
**Prediction:**	neutral	neutral	neutral	neutral	neutral	neutral
**Score:**	-1.0000	10.1300	0.6013	0.7575	0.0000	0.1900
**Exp. accuracy:**	0.96	0.88	0.96	0.69	0.93	0.60

External links

[dbSNP] [GenBank] [RegulomeDB] [UCSC] [Ensemble]

**Variant:** 21 : 32645713, $C \rightarrow$T					
**Position:**	chr21:32645713	**Ref. allele:**	C	**Alt. allele:**	T
**Region:**	exonic	**Exonic. func.:**	synonymous	**Gene ID:**	SYNJ1
**RS number:**	-	**Clin. sign.:**	-	-	

Results

**Tool:**	**PredictSNP2**	**CADD**	**DANN**	**FATHMM**	**FunSeq2**	**GWAVA**
**Prediction:**	neutral	neutral	deleterious	neutral	neutral	neutral
**Score:**	-0.5474	6.2110	0.9147	0.0309	0.0000	0.2400
**Exp. accuracy:**	0.93	0.93	0.87	0.96	0.93	0.58

External links

[GenBank] [UCSC] [Ensemble]

Variant: 21 : 32645752, $C \rightarrow$A					
**Position:**	chr21:32645752	**Ref. allele:**	C	**Alt. allele:**	A
**Region:**	exonic	**Exonic. func.:**	synonymous	**Gene ID:**	SYNJ1
**RS number:**	rs376395608	**Clin. sign.:**	-	-	

Results

**Tool:**	**PredictSNP2**	**CADD**	**DANN**	**FATHMM**	**FunSeq2**	**GWAVA**
**Prediction:**	neutral	neutral	neutral	neutral	neutral	neutral
**Score:**	-1.0000	9.5660	0.7063	0.4347	0.0000	0.2800
**Exp. accuracy:**	0.96	0.91	0.93	0.72	0.93	0.55

External links

[dbSNP] [GenBank] [RegulomeDB] [UCSC] [Ensemble]

Variant: 21 : 32645752, $C \rightarrow$T					
**Position:**	chr21:32645752	**Ref. allele:**	C	**Alt. allele:**	T
**Region:**	exonic	**Exonic. func.:**	synonymous	**Gene ID:**	SYNJ1
**RS number:**	-	**Clin. sign.:**	-	-	

Results

**Tool:**	**PredictSNP2**	**CADD**	**DANN**	**FATHMM**	**FunSeq2**	**GWAVA**
**Prediction:**	neutral	neutral	neutral	neutral	neutral	neutral
**Score:**	-1.0000	9.4420	0.8825	0.3224	0.0000	0.2800
**Exp. accuracy:**	0.96	0.91	0.90	0.73	0.93	0.55

External links

[GenBank] [UCSC] [Ensemble]

Variant: 21 : 32646403, $G \rightarrow$A					
**Position:**	chr21:32646403	**Ref. allele:**	G	**Alt. allele:**	A
**Region:**	exonic	**Exonic. func.:**	synonymous	**Gene ID:**	SYNJ1
**RS number:**	-	**Clin. sign.:**	-	-	

Results

**Tool:**	**PredictSNP2**	**CADD**	**DANN**	**FATHMM**	**FunSeq2**	**GWAVA**
**Prediction:**	neutral	neutral	neutral	neutral	neutral	neutral
**Score:**	-1.0000	2.9250	0.5066	0.0144	0.0000	0.2400
**Exp. accuracy:**	0.96	0.95	0.97	0.96	0.93	0.58

External links

[GenBank] [UCSC] [Ensemble]

**Variant:** 21 : 32646412, $A \rightarrow$G					
**Position:**	chr21:32646412	**Ref. allele:**	A	**Alt. allele:**	G
**Region:**	exonic	**Exonic. func.:**	synonymous	**Gene ID:**	SYNJ1
**RS number:**	-	**Clin. sign.:**	-	-	

Results

**Tool:**	**PredictSNP2**	**CADD**	**DANN**	**FATHMM**	**FunSeq2**	**GWAVA**
**Prediction:**	neutral	neutral	neutral	neutral	neutral	neutral
**Score:**	-1.0000	1.2660	0.4711	0.0991	0.0000	0.3100
**Exp. accuracy:**	0.96	0.95	0.97	0.87	0.93	0.53

External links

[GenBank] [UCSC] [Ensemble]

**Variant:** 21 : 32646430, $T \rightarrow$C					
**Position:**	chr21:32646430	**Ref. allele:**	T	**Alt. allele:**	C
**Region:**	exonic	**Exonic. func.:**	synonymous	**Gene ID:**	SYNJ1
**RS number:**	-	**Clin. sign.:**	-	-	

Results

**Tool:**	**PredictSNP2**	**CADD**	**DANN**	**FATHMM**	**FunSeq2**	**GWAVA**
**Prediction:**	neutral	neutral	neutral	neutral	neutral	neutral
**Score:**	-1.0000	4.1360	0.7459	0.3716	0.0000	0.2800
**Exp. accuracy:**	0.96	0.94	0.91	0.72	0.93	0.55

External links

[GenBank] [UCSC] [Ensemble]

**Variant:** 21 : 32646445, $G \rightarrow$A					
**Position:**	chr21:32646445	**Ref. allele:**	G	**Alt. allele:**	A
**Region:**	exonic	**Exonic. func.:**	synonymous	**Gene ID:**	SYNJ1
**RS number:**	-	**Clin. sign.:**	-	-	

Results

**Tool:**	**PredictSNP2**	**CADD**	**DANN**	**FATHMM**	**FunSeq2**	**GWAVA**
**Prediction:**	neutral	neutral	neutral	neutral	neutral	neutral
**Score:**	-1.0000	8.1540	0.4897	0.0569	0.0000	0.2400
**Exp. accuracy:**	0.96	0.91	0.97	0.92	0.93	0.58

External links

[GenBank] [UCSC] [Ensemble]

**Variant:** 21 : 32646460, $A\to$G					
**Position:**	chr21:32646460	**Ref. allele:**	A	**Alt. allele:**	G
**Region:**	exonic	**Exonic. func.:**	synonymous	**Gene ID:**	SYNJ1
**RS number:**	-	**Clin. sign.:**	-	-	

Results

**Tool:**	**PredictSNP2**	**CADD**	**DANN**	**FATHMM**	**FunSeq2**	**GWAVA**
**Prediction:**	neutral	neutral	neutral	neutral	neutral	neutral
**Score:**	-1.0000	0.3150	0.5101	0.0398	0.0000	0.2700
**Exp. accuracy:**	0.96	0.95	0.97	0.96	0.93	0.55

External links

[GenBank] [UCSC] [Ensemble]

**Variant:** 21 : 32646487, $G\to$A					
**Position:**	chr21:32646487	**Ref. allele:**	G	**Alt. allele:**	A
**Region:**	exonic	**Exonic. func.:**	synonymous	**Gene ID:**	SYNJ1
**RS number:**	-	**Clin. sign.:**	-	-	

Results

**Tool:**	**PredictSNP2**	**CADD**	**DANN**	**FATHMM**	**FunSeq2**	**GWAVA**
**Prediction:**	neutral	neutral	neutral	deleterious	neutral	neutral
**Score:**	-0.5837	11.9300	0.6914	0.8450	0.0000	0.3300
**Exp. accuracy:**	0.93	0.83	0.94	0.57	0.93	0.56

External links

[GenBank] [UCSC] [Ensemble]

**Variant:** 21 : 32646490, $G\to$C					
**Position:**	chr21:32646490	**Ref. allele:**	G	**Alt. allele:**	C
**Region:**	exonic	**Exonic. func.:**	synonymous	**Gene ID:**	SYNJ1
**RS number:**	-	**Clin. sign.:**	-	-	

Results

**Tool:**	**PredictSNP2**	**CADD**	**DANN**	**FATHMM**	**FunSeq2**	**GWAVA**
**Prediction:**	neutral	neutral	neutral	deleterious	neutral	neutral
**Score:**	-0.4537	10.0500	0.7592	0.9539	0.0000	0.4100
**Exp. accuracy:**	0.93	0.91	0.90	0.92	0.93	0.56

External links

[GenBank] [UCSC] [Ensemble]

**Variant:** 21 : 32646496, $A \to$C					
**Position:**	chr21:32646496	**Ref. allele:**	A	**Alt. allele:**	C
**Region:**	exonic	**Exonic. func.:**	synonymous	**Gene ID:**	SYNJ1
**RS number:**	-	**Clin. sign.:**	-	-	

Results

**Tool:**	**PredictSNP2**	**CADD**	**DANN**	**FATHMM**	**FunSeq2**	**GWAVA**
**Prediction:**	neutral	neutral	neutral	deleterious	neutral	neutral
**Score:**	-0.5982	0.2960	0.5772	0.7913	0.0000	0.3100
**Exp. accuracy:**	0.93	0.95	0.97	0.57	0.93	0.53

External links

[GenBank] [UCSC] [Ensemble]

**Variant:** 21 : 32646496, $A \to$G					
**Position:**	chr21:32646496	**Ref. allele:**	A	**Alt. allele:**	G
**Region:**	exonic	**Exonic. func.:**	synonymous	**Gene ID:**	SYNJ1
**RS number:**	-	**Clin. sign.:**	-	-	

Results

**Tool:**	**PredictSNP2**	**CADD**	**DANN**	**FATHMM**	**FunSeq2**	**GWAVA**
**Prediction:**	neutral	neutral	neutral	neutral	neutral	neutral
**Score:**	-1.0000	5.3940	0.5963	0.1808	0.0000	0.3100
**Exp. accuracy:**	0.96	0.92	0.96	0.80	0.93	0.53

External links

[GenBank] [UCSC] [Ensemble]

**Variant:** 21 : 32646517, $G \to$A					
**Position:**	chr21:32646517	**Ref. allele:**	G	**Alt. allele:**	A
**Region:**	exonic	**Exonic. func.:**	synonymous	**Gene ID:**	SYNJ1
**RS number:**	-	**Clin. sign.:**	-	-	

Results

**Tool:**	**PredictSNP2**	**CADD**	**DANN**	**FATHMM**	**FunSeq2**	**GWAVA**
**Prediction:**	neutral	neutral	neutral	neutral	neutral	neutral
**Score:**	-1.0000	8.9180	0.6091	0.0929	0.0000	0.3100
**Exp. accuracy:**	0.96	0.92	0.96	0.87	0.93	0.53

External links

[GenBank] [UCSC] [Ensemble]

**Variant:** 21 : 32646580, $A \rightarrow$T					
**Position:**	chr21:32646580	**Ref. allele:**	A	**Alt. allele:**	T
**Region:**	exonic	**Exonic. func.:**	synonymous	**Gene ID:**	SYNJ1
**RS number:**	rs141637663	**Clin. sign.:**	-	-	

Results

**Tool:**	**PredictSNP2**	**CADD**	**DANN**	**FATHMM**	**FunSeq2**	**GWAVA**
**Prediction:**	neutral	neutral	neutral	neutral	neutral	neutral
**Score:**	-1.0000	0.0050	0.5353	0.0390	0.0000	0.3500
**Exp. accuracy:**	0.96	0.95	0.97	0.96	0.93	0.54

External links

[dbSNP] [GenBank] [HaploReg] [RegulomeDB] [UCSC] [Ensemble]

**Variant:** 21 : 32646583, $A \rightarrow$G					
**Position:**	chr21:32646583	**Ref. allele:**	A	**Alt. allele:**	G
**Region:**	exonic	**Exonic. func.:**	synonymous	**Gene ID:**	SYNJ1
**RS number:**	-	**Clin. sign.:**	-	-	

Results

**Tool:**	**PredictSNP2**	**CADD**	**DANN**	**FATHMM**	**FunSeq2**	**GWAVA**
**Prediction:**	neutral	neutral	neutral	deleterious	neutral	neutral
**Score:**	-0.5406	5.7140	0.6696	0.8865	0.0000	0.3500
**Exp. accuracy:**	0.93	0.92	0.94	0.68	0.93	0.54

External links

[GenBank] [UCSC] [Ensemble]

**Variant:** 21 : 32646595, $A \rightarrow$G					
**Position:**	chr21:32646595	**Ref. allele:**	A	**Alt. allele:**	G
**Region:**	exonic	**Exonic. func.:**	synonymous	**Gene ID:**	SYNJ1
**RS number:**	-	**Clin. sign.:**	-	-	

Results

**Tool:**	**PredictSNP2**	**CADD**	**DANN**	**FATHMM**	**FunSeq2**	**GWAVA**
**Prediction:**	neutral	neutral	neutral	neutral	neutral	neutral
**Score:**	-1.0000	5.0290	0.6510	0.0212	0.0000	0.3600
**Exp. accuracy:**	0.96	0.93	0.96	0.96	0.93	0.55

External links

[GenBank] [UCSC] [Ensemble]

**Variant:** 21 : 32650212, $T \to$G					
**Position:**	chr21:32650212	**Ref. allele:**	T	**Alt. allele:**	G
**Region:**	exonic	**Exonic. func.:**	synonymous	**Gene ID:**	SYNJ1
**RS number:**	-	**Clin. sign.:**	-	-	

Results

**Tool:**	**PredictSNP2**	**CADD**	**DANN**	**FATHMM**	**FunSeq2**	**GWAVA**
**Prediction:**	neutral	neutral	neutral	neutral	neutral	deleterious
**Score:**	-1.0000	0.7940	0.4203	0.0117	0.0000	0.5000
**Exp. accuracy:**	0.96	0.95	0.97	0.96	0.93	0.51

External links

[GenBank] [UCSC] [Ensemble]

**Variant:** 21 : 32650224, $A \to$G					
**Position:**	chr21:32650224	**Ref. allele:**	A	**Alt. allele:**	G
**Region:**	exonic	**Exonic. func.:**	synonymous	**Gene ID:**	SYNJ1
**RS number:**	rs111774827	**Clin. sign.:**	-	-	

Results

**Tool:**	**PredictSNP2**	**CADD**	**DANN**	**FATHMM**	**FunSeq2**	**GWAVA**
**Prediction:**	neutral	neutral	neutral	neutral	neutral	deleterious
**Score:**	-1.0000	1.7200	0.7276	0.0524	0.0000	0.5300
**Exp. accuracy:**	0.96	0.95	0.93	0.94	0.93	0.64

External links

[dbSNP] [GenBank] [HaploReg] [RegulomeDB] [UCSC] [Ensemble]

**Variant:** 21 : 32650229, $G \to$A					
**Position:**	chr21:32650229	**Ref. allele:**	G	**Alt. allele:**	A
**Region:**	exonic	**Exonic. func.:**	synonymous	**Gene ID:**	SYNJ1
**RS number:**	-	**Clin. sign.:**	-	-	

Results

**Tool:**	**PredictSNP2**	**CADD**	**DANN**	**FATHMM**	**FunSeq2**	**GWAVA**
**Prediction:**	neutral	neutral	neutral	deleterious	neutral	deleterious
**Score:**	-0.5231	9.2030	0.7341	0.8977	0.0000	0.6100
**Exp. accuracy:**	0.93	0.92	0.91	0.71	0.93	0.64

External links

[GenBank] [UCSC] [Ensemble]

**Variant:**	$21:32650230, G \to$ A				
**Position:**	chr21:32650230	**Ref. allele:**	G	**Alt. allele:**	A
**Region:**	exonic	**Exonic. func.:**	synonymous	**Gene ID:**	SYNJ1
**RS number:**	-	**Clin. sign.:**	-	-	

Results

**Tool:**	**PredictSNP2**	**CADD**	**DANN**	**FATHMM**	**FunSeq2**	**GWAVA**
**Prediction:**	neutral	neutral	neutral	neutral	neutral	deleterious
**Score:**	-1.0000	9.8810	0.6832	0.7438	0.0000	0.5900
**Exp. accuracy:**	0.96	0.91	0.94	0.69	0.93	0.62

External links

[GenBank] [UCSC] [Ensemble]

**Variant:**	$21:32650230, G \to$ T				
**Position:**	chr21:32650230	**Ref. allele:**	G	**Alt. allele:**	T
**Region:**	exonic	**Exonic. func.:**	synonymous	**Gene ID:**	SYNJ1
**RS number:**	rs191969474	**Clin. sign.:**	-	-	

Results

**Tool:**	**PredictSNP2**	**CADD**	**DANN**	**FATHMM**	**FunSeq2**	**GWAVA**
**Prediction:**	neutral	neutral	neutral	deleterious	neutral	deleterious
**Score:**	-0.5834	3.7680	0.6814	0.8286	0.0000	0.5900
**Exp. accuracy:**	0.93	0.94	0.94	0.57	0.93	0.62

External links

[dbSNP] [GenBank] [HaploReg] [RegulomeDB] [UCSC] [Ensemble]

**Variant:**	$21:32650245, T \to$ C				
**Position:**	chr21:32650245	**Ref. allele:**	T	**Alt. allele:**	C
**Region:**	exonic	**Exonic. func.:**	synonymous	**Gene ID:**	SYNJ1
**RS number:**	rs115204185	**Clin. sign.:**	-	-	

Results

**Tool:**	**PredictSNP2**	**CADD**	**DANN**	**FATHMM**	**FunSeq2**	**GWAVA**
**Prediction:**	neutral	neutral	neutral	neutral	neutral	deleterious
**Score:**	-1.0000	0.5940	0.4993	0.0509	0.0000	0.5300
**Exp. accuracy:**	0.96	0.95	0.97	0.94	0.93	0.64

External links

[dbSNP] [GenBank] [HaploReg] [RegulomeDB] [UCSC] [Ensemble]

**Variant:** 21 : 32650266, $T \rightarrow$C					
**Position:**	chr21:32650266	**Ref. allele:**	T	**Alt. allele:**	C
**Region:**	exonic	**Exonic. func.:**	synonymous	**Gene ID:**	SYNJ1
**RS number:**	-	**Clin. sign.:**	-	-	

Results

**Tool:**	**PredictSNP2**	**CADD**	**DANN**	**FATHMM**	**FunSeq2**	**GWAVA**
**Prediction:**	neutral	neutral	neutral	neutral	neutral	deleterious
**Score:**	-1.0000	7.5390	0.6618	0.7087	0.0000	0.6800
**Exp. accuracy:**	0.96	0.91	0.95	0.69	0.93	0.70

External links

[GenBank] [UCSC] [Ensemble]

**Variant:** 21 : 32650284, $T \rightarrow$C					
**Position:**	chr21:32650284	**Ref. allele:**	T	**Alt. allele:**	C
**Region:**	exonic	**Exonic. func.:**	synonymous	**Gene ID:**	SYNJ1
**RS number:**	-	**Clin. sign.:**	-	-	

Results

**Tool:**	**PredictSNP2**	**CADD**	**DANN**	**FATHMM**	**FunSeq2**	**GWAVA**
**Prediction:**	neutral	neutral	neutral	neutral	neutral	deleterious
**Score:**	-1.0000	0.2240	0.6264	0.6602	0.0000	0.7100
**Exp. accuracy:**	0.96	0.95	0.97	0.69	0.93	0.70

External links

[GenBank] [UCSC] [Ensemble]

**Variant:** 21 : 32653302, $G \rightarrow$A					
**Position:**	chr21:32653302	**Ref. allele:**	G	**Alt. allele:**	A
**Region:**	exonic	**Exonic. func.:**	synonymous	**Gene ID:**	SYNJ1
**RS number:**	rs374794895	**Clin. sign.:**	-	-	

Results

**Tool:**	**PredictSNP2**	**CADD**	**DANN**	**FATHMM**	**FunSeq2**	**GWAVA**
**Prediction:**	neutral	deleterious	neutral	deleterious	neutral	neutral
**Score:**	-0.1244	16.8700	0.7756	0.7957	0.0000	0.3900
**Exp. accuracy:**	0.88	0.58	0.90	0.57	0.93	0.52

External links

[dbSNP] [GenBank] [RegulomeDB] [UCSC] [Ensemble]

**Variant:** 21 : 32656743, $G \to$ A					
**Position:**	chr21:32656743	**Ref. allele:**	G	**Alt. allele:**	A
**Region:**	exonic	**Exonic. func.:**	synonymous	**Gene ID:**	SYNJ1
**RS number:**	-	**Clin. sign.:**	-	-	

Results

**Tool:**	**PredictSNP2**	**CADD**	**DANN**	**FATHMM**	**FunSeq2**	**GWAVA**
**Prediction:**	neutral	neutral	neutral	neutral	neutral	deleterious
**Score:**	-1.0000	10.0300	0.6158	0.5623	0.0000	0.6400
**Exp. accuracy:**	0.96	0.91	0.96	0.70	0.93	0.68

External links

[GenBank] [UCSC] [Ensemble]

**Variant:** 21 : 32656767, $T \to$ C					
**Position:**	chr21:32656767	**Ref. allele:**	T	**Alt. allele:**	C
**Region:**	exonic	**Exonic. func.:**	synonymous	**Gene ID:**	SYNJ1
**RS number:**	-	**Clin. sign.:**	-	-	

Results

**Tool:**	**PredictSNP2**	**CADD**	**DANN**	**FATHMM**	**FunSeq2**	**GWAVA**
**Prediction:**	neutral	neutral	neutral	deleterious	neutral	deleterious
**Score:**	-0.5847	5.8230	0.7158	0.8046	0.0000	0.6500
**Exp. accuracy:**	0.93	0.92	0.93	0.57	0.93	0.70

External links

[GenBank] [UCSC] [Ensemble]

**Variant:** 21 : 32656791, $C \to$ T					
**Position:**	chr21:32656791	**Ref. allele:**	C	**Alt. allele:**	T
**Region:**	exonic	**Exonic. func.:**	synonymous	**Gene ID:**	SYNJ1
**RS number:**	rs115263361	**Clin. sign.:**	-	-	

Results

**Tool:**	**PredictSNP2**	**CADD**	**DANN**	**FATHMM**	**FunSeq2**	**GWAVA**
**Prediction:**	neutral	neutral	neutral	deleterious	neutral	deleterious
**Score:**	-0.4960	11.5700	0.7265	0.9126	0.0000	0.6500
**Exp. accuracy:**	0.93	0.88	0.93	0.77	0.93	0.70

External links

[dbSNP] [GenBank] [HaploReg] [RegulomeDB] [UCSC] [Ensemble]

**Variant:** 21 : 32656793, $A \to$G					
**Position:**	chr21:32656793	**Ref. allele:**	A	**Alt. allele:**	G
**Region:**	exonic	**Exonic. func.:**	synonymous	**Gene ID:**	SYNJ1
**RS number:**	-	**Clin. sign.:**	-	-	

Results

**Tool:**	**PredictSNP2**	**CADD**	**DANN**	**FATHMM**	**FunSeq2**	**GWAVA**
**Prediction:**	neutral	neutral	neutral	neutral	neutral	deleterious
**Score:**	-1.0000	4.0080	0.7407	0.1025	0.0000	0.6100
**Exp. accuracy:**	0.96	0.94	0.91	0.87	0.93	0.64

External links

[GenBank] [UCSC] [Ensemble]

**Variant:** 21 : 32656821, $T \to$G					
**Position:**	chr21:32656821	**Ref. allele:**	T	**Alt. allele:**	G
**Region:**	exonic	**Exonic. func.:**	synonymous	**Gene ID:**	SYNJ1
**RS number:**	-	**Clin. sign.:**	-	-	

Results

**Tool:**	**PredictSNP2**	**CADD**	**DANN**	**FATHMM**	**FunSeq2**	**GWAVA**
**Prediction:**	neutral	neutral	neutral	neutral	neutral	deleterious
**Score:**	-1.0000	6.4490	0.5096	0.0558	0.0000	0.5700
**Exp. accuracy:**	0.96	0.93	0.97	0.94	0.93	0.68

External links

[GenBank] [UCSC] [Ensemble]

**Variant:** 21 : 32656851, $C \to$T					
**Position:**	chr21:32656851	**Ref. allele:**	C	**Alt. allele:**	T
**Region:**	exonic	**Exonic. func.:**	synonymous	**Gene ID:**	SYNJ1
**RS number:**	rs375526059	**Clin. sign.:**	-	-	

Results

**Tool:**	**PredictSNP2**	**CADD**	**DANN**	**FATHMM**	**FunSeq2**	**GWAVA**
**Prediction:**	neutral	neutral	neutral	neutral	neutral	deleterious
**Score:**	-1.0000	12.1000	0.4774	0.7066	0.0000	0.6600
**Exp. accuracy:**	0.96	0.83	0.97	0.69	0.93	0.70

External links

[dbSNP] [GenBank] [RegulomeDB] [UCSC] [Ensemble]

**Variant:** $21:32656860, T{\to}$C					
**Position:**	chr21:32656860	**Ref. allele:**	T	**Alt. allele:**	C
**Region:**	exonic	**Exonic. func.:**	synonymous	**Gene ID:**	SYNJ1
**RS number:**	-	**Clin. sign.:**	-	-	

Results

**Tool:**	**PredictSNP2**	**CADD**	**DANN**	**FATHMM**	**FunSeq2**	**GWAVA**
**Prediction:**	neutral	neutral	neutral	deleterious	neutral	deleterious
**Score:**	-0.5929	1.5310	0.6071	0.8319	0.0000	0.6600
**Exp. accuracy:**	0.93	0.95	0.96	0.57	0.93	0.70

External links

[GenBank] [UCSC] [Ensemble]

**Variant:** $21:32656890, G{\to}$A					
**Position:**	chr21:32656890	**Ref. allele:**	G	**Alt. allele:**	A
**Region:**	exonic	**Exonic. func.:**	synonymous	**Gene ID:**	SYNJ1
**RS number:**	rs373366653	**Clin. sign.:**	-	-	

Results

**Tool:**	**PredictSNP2**	**CADD**	**DANN**	**FATHMM**	**FunSeq2**	**GWAVA**
**Prediction:**	neutral	neutral	neutral	neutral	neutral	deleterious
**Score:**	-1.0000	10.5700	0.6107	0.5430	0.0000	0.7000
**Exp. accuracy:**	0.96	0.88	0.96	0.70	0.93	0.70

External links

[dbSNP] [GenBank] [RegulomeDB] [UCSC] [Ensemble]

**Variant:** $21:32656896, G{\to}$A					
**Position:**	chr21:32656896	**Ref. allele:**	G	**Alt. allele:**	A
**Region:**	exonic	**Exonic. func.:**	synonymous	**Gene ID:**	SYNJ1
**RS number:**	-	**Clin. sign.:**	-	-	

Results

**Tool:**	**PredictSNP2**	**CADD**	**DANN**	**FATHMM**	**FunSeq2**	**GWAVA**
**Prediction:**	neutral	neutral	neutral	neutral	neutral	deleterious
**Score:**	-1.0000	10.2000	0.5438	0.0399	0.0000	0.6000
**Exp. accuracy:**	0.96	0.88	0.97	0.96	0.93	0.64

External links

[GenBank] [UCSC] [Ensemble]

**Variant:** 21 : 32657035, $A \rightarrow$G					
**Position:**	chr21:32657035	**Ref. allele:**	A	**Alt. allele:**	G
**Region:**	exonic	**Exonic. func.:**	synonymous	**Gene ID:**	SYNJ1
**RS number:**	rs61756207	**Clin. sign.:**	-	-	

Results

**Tool:**	**PredictSNP2**	**CADD**	**DANN**	**FATHMM**	**FunSeq2**	**GWAVA**
**Prediction:**	neutral	neutral	neutral	neutral	neutral	neutral
**Score:**	-1.0000	4.7310	0.4105	0.0695	0.0000	0.3600
**Exp. accuracy:**	0.96	0.93	0.97	0.90	0.93	0.55

External links

[dbSNP] [GenBank] [HaploReg] [RegulomeDB] [UCSC] [Ensemble]

**Variant:** 21 : 32657038, $G \rightarrow$A					
**Position:**	chr21:32657038	**Ref. allele:**	G	**Alt. allele:**	A
**Region:**	exonic	**Exonic. func.:**	synonymous	**Gene ID:**	SYNJ1
**RS number:**	-	**Clin. sign.:**	-	-	

Results

**Tool:**	**PredictSNP2**	**CADD**	**DANN**	**FATHMM**	**FunSeq2**	**GWAVA**
**Prediction:**	neutral	neutral	neutral	deleterious	neutral	neutral
**Score:**	-0.5975	3.6800	0.3607	0.8619	0.0000	0.3300
**Exp. accuracy:**	0.93	0.94	0.97	0.57	0.93	0.56

External links

[GenBank] [UCSC] [Ensemble]

**Variant:** 21 : 32657041, $C \rightarrow$A					
**Position:**	chr21:32657041	**Ref. allele:**	C	**Alt. allele:**	A
**Region:**	exonic	**Exonic. func.:**	synonymous	**Gene ID:**	SYNJ1
**RS number:**	-	**Clin. sign.:**	-	-	

Results

**Tool:**	**PredictSNP2**	**CADD**	**DANN**	**FATHMM**	**FunSeq2**	**GWAVA**
**Prediction:**	neutral	neutral	neutral	deleterious	neutral	neutral
**Score:**	-0.4412	13.2000	0.7650	0.9481	0.0000	0.4200
**Exp. accuracy:**	0.88	0.74	0.90	0.91	0.93	0.54

External links

[GenBank] [UCSC] [Ensemble]

**Variant:**	21 : 32657062, $C \rightarrow$A				
**Position:**	chr21:32657062	**Ref. allele:**	C	**Alt. allele:**	A
**Region:**	exonic	**Exonic. func.:**	synonymous	**Gene ID:**	SYNJ1
**RS number:**	-	**Clin. sign.:**	-	-	

Results

**Tool:**	**PredictSNP2**	**CADD**	**DANN**	**FATHMM**	**FunSeq2**	**GWAVA**
**Prediction:**	neutral	neutral	neutral	neutral	neutral	neutral
**Score:**	-1.0000	15.5600	0.4748	0.0470	0.0000	0.4400
**Exp. accuracy:**	0.96	0.73	0.97	0.94	0.93	0.54

External links

[GenBank] [UCSC] [Ensemble]

**Variant:**	21 : 32657062, $C \rightarrow$T				
**Position:**	chr21:32657062	**Ref. allele:**	C	**Alt. allele:**	T
**Region:**	exonic	**Exonic. func.:**	synonymous	**Gene ID:**	SYNJ1
**RS number:**	-	**Clin. sign.:**	-	-	

Results

**Tool:**	**PredictSNP2**	**CADD**	**DANN**	**FATHMM**	**FunSeq2**	**GWAVA**
**Prediction:**	neutral	neutral	neutral	neutral	neutral	neutral
**Score:**	-1.0000	14.1700	0.4416	0.0328	0.0000	0.4400
**Exp. accuracy:**	0.96	0.73	0.97	0.96	0.93	0.54

External links

[GenBank] [UCSC] [Ensemble]

**Variant:**	21 : 32657095, $A \rightarrow$G				
**Position:**	chr21:32657095	**Ref. allele:**	A	**Alt. allele:**	G
**Region:**	exonic	**Exonic. func.:**	synonymous	**Gene ID:**	SYNJ1
**RS number:**	-	**Clin. sign.:**	-	-	

Results

**Tool:**	**PredictSNP2**	**CADD**	**DANN**	**FATHMM**	**FunSeq2**	**GWAVA**
**Prediction:**	neutral	neutral	neutral	neutral	neutral	neutral
**Score:**	-1.0000	6.3040	0.4945	0.1296	0.0000	0.4200
**Exp. accuracy:**	0.96	0.93	0.97	0.85	0.93	0.54

External links

[GenBank] [UCSC] [Ensemble]

**Variant:** $21:32657738, C{\rightarrow}$T					
**Position:**	chr21:32657738	**Ref. allele:**	C	**Alt. allele:**	T
**Region:**	exonic	**Exonic. func.:**	synonymous	**Gene ID:**	SYNJ1
**RS number:**	rs143244716	**Clin. sign.:**	-	-	

Results

**Tool:**	**PredictSNP2**	**CADD**	**DANN**	**FATHMM**	**FunSeq2**	**GWAVA**
**Prediction:**	neutral	neutral	neutral	neutral	neutral	neutral
**Score:**	-1.0000	13.4900	0.5785	0.5982	0.0000	0.3700
**Exp. accuracy:**	0.96	0.73	0.97	0.69	0.93	0.56

External links

[dbSNP] [GenBank] [HaploReg] [RegulomeDB] [UCSC] [Ensemble]

**Variant:** $21:32657774, G{\rightarrow}$A					
**Position:**	chr21:32657774	**Ref. allele:**	G	**Alt. allele:**	A
**Region:**	exonic	**Exonic. func.:**	synonymous	**Gene ID:**	SYNJ1
**RS number:**	-	**Clin. sign.:**	-	-	

Results

**Tool:**	**PredictSNP2**	**CADD**	**DANN**	**FATHMM**	**FunSeq2**	**GWAVA**
**Prediction:**	neutral	neutral	neutral	neutral	neutral	neutral
**Score:**	-1.0000	8.5780	0.5745	0.2086	0.0000	0.3500
**Exp. accuracy:**	0.96	0.92	0.97	0.77	0.93	0.54

External links

[GenBank] [UCSC] [Ensemble]

**Variant:** $21:32657780, G{\rightarrow}$A					
**Position:**	chr21:32657780	**Ref. allele:**	G	**Alt. allele:**	A
**Region:**	exonic	**Exonic. func.:**	synonymous	**Gene ID:**	SYNJ1
**RS number:**	-	**Clin. sign.:**	-	-	

Results

**Tool:**	**PredictSNP2**	**CADD**	**DANN**	**FATHMM**	**FunSeq2**	**GWAVA**
**Prediction:**	neutral	neutral	neutral	deleterious	neutral	neutral
**Score:**	-0.5462	9.4670	0.6116	0.8871	0.0000	0.3500
**Exp. accuracy:**	0.93	0.91	0.96	0.68	0.93	0.54

External links

[GenBank] [UCSC] [Ensemble]

**Variant:** 21 : 32657792, $G \rightarrow$C					
**Position:**	chr21:32657792	**Ref. allele:**	G	**Alt. allele:**	C
**Region:**	exonic	**Exonic. func.:**	synonymous	**Gene ID:**	SYNJ1
**RS number:**	-	**Clin. sign.:**	-	-	

Results

**Tool:**	**PredictSNP2**	**CADD**	**DANN**	**FATHMM**	**FunSeq2**	**GWAVA**
**Prediction:**	neutral	neutral	neutral	deleterious	neutral	neutral
**Score:**	-0.4865	6.3800	0.6285	0.9269	0.0000	0.4500
**Exp. accuracy:**	0.93	0.93	0.97	0.82	0.93	0.54

External links

[GenBank] [UCSC] [Ensemble]

**Variant:** 21 : 32657804, $G \rightarrow$A					
**Position:**	chr21:32657804	**Ref. allele:**	G	**Alt. allele:**	A
**Region:**	exonic	**Exonic. func.:**	synonymous	**Gene ID:**	SYNJ1
**RS number:**	rs374390032	**Clin. sign.:**	-	-	

Results

**Tool:**	**PredictSNP2**	**CADD**	**DANN**	**FATHMM**	**FunSeq2**	**GWAVA**
**Prediction:**	neutral	neutral	neutral	neutral	neutral	neutral
**Score:**	-1.0000	6.9650	0.5392	0.1236	0.0000	0.3900
**Exp. accuracy:**	0.96	0.92	0.97	0.85	0.93	0.52

External links

[dbSNP] [GenBank] [RegulomeDB] [UCSC] [Ensemble]

**Variant:** 21 : 32657825, $A \rightarrow$G					
**Position:**	chr21:32657825	**Ref. allele:**	A	**Alt. allele:**	G
**Region:**	exonic	**Exonic. func.:**	synonymous	**Gene ID:**	SYNJ1
**RS number:**	-	**Clin. sign.:**	-	-	

Results

**Tool:**	**PredictSNP2**	**CADD**	**DANN**	**FATHMM**	**FunSeq2**	**GWAVA**
**Prediction:**	neutral	neutral	neutral	neutral	neutral	neutral
**Score:**	-1.0000	6.7350	0.3924	0.5107	0.0000	0.3800
**Exp. accuracy:**	0.96	0.92	0.97	0.70	0.93	0.55

External links

[GenBank] [UCSC] [Ensemble]

**Variant:** 21 : 32657831, $C \rightarrow$T					
**Position:**	chr21:32657831	**Ref. allele:**	C	**Alt. allele:**	T
**Region:**	exonic	**Exonic. func.:**	synonymous	**Gene ID:**	SYNJ1
**RS number:**	rs61753644	**Clin. sign.:**	-	-	

Results

**Tool:**	**PredictSNP2**	**CADD**	**DANN**	**FATHMM**	**FunSeq2**	**GWAVA**
**Prediction:**	neutral	neutral	neutral	neutral	neutral	neutral
**Score:**	-1.0000	14.3300	0.7188	0.0717	0.0000	0.4200
**Exp. accuracy:**	0.96	0.73	0.93	0.90	0.93	0.54

External links

[dbSNP] [GenBank] [HaploReg] [RegulomeDB] [UCSC] [Ensemble]

**Variant:** 21 : 32657840, $G \rightarrow$A					
**Position:**	chr21:32657840	**Ref. allele:**	G	**Alt. allele:**	A
**Region:**	exonic	**Exonic. func.:**	synonymous	**Gene ID:**	SYNJ1
**RS number:**	rs367656251	**Clin. sign.:**	-	-	

Results

**Tool:**	**PredictSNP2**	**CADD**	**DANN**	**FATHMM**	**FunSeq2**	**GWAVA**
**Prediction:**	neutral	neutral	neutral	neutral	neutral	neutral
**Score:**	-1.0000	10.7000	0.6158	0.6999	0.0000	0.4400
**Exp. accuracy:**	0.96	0.88	0.96	0.69	0.93	0.54

External links

[dbSNP] [GenBank] [RegulomeDB] [UCSC] [Ensemble]

**Variant:** 21 : 32657846, $C \rightarrow$T					
**Position:**	chr21:32657846	**Ref. allele:**	C	**Alt. allele:**	T
**Region:**	exonic	**Exonic. func.:**	synonymous	**Gene ID:**	SYNJ1
**RS number:**	-	**Clin. sign.:**	-	-	

Results

**Tool:**	**PredictSNP2**	**CADD**	**DANN**	**FATHMM**	**FunSeq2**	**GWAVA**
**Prediction:**	neutral	neutral	neutral	neutral	neutral	neutral
**Score:**	-1.0000	13.5000	0.5218	0.3516	0.0000	0.4500
**Exp. accuracy:**	0.96	0.73	0.97	0.73	0.93	0.54

External links

[GenBank] [UCSC] [Ensemble]

**Variant:** 21 : 32657852, $T \rightarrow$C					
**Position:**	chr21:32657852	**Ref. allele:**	T	**Alt. allele:**	C
**Region:**	exonic	**Exonic. func.:**	synonymous	**Gene ID:**	SYNJ1
**RS number:**	-	**Clin. sign.:**	-	-	

Results

**Tool:**	**PredictSNP2**	**CADD**	**DANN**	**FATHMM**	**FunSeq2**	**GWAVA**
**Prediction:**	neutral	neutral	neutral	neutral	neutral	neutral
**Score:**	-1.0000	1.1260	0.5781	0.5349	0.0000	0.3600
**Exp. accuracy:**	0.96	0.95	0.97	0.70	0.93	0.55

External links

[GenBank] [UCSC] [Ensemble]

**Variant:** 21 : 32657870, $A \rightarrow$G					
**Position:**	chr21:32657870	**Ref. allele:**	A	**Alt. allele:**	G
**Region:**	exonic	**Exonic. func.:**	synonymous	**Gene ID:**	SYNJ1
**RS number:**	rs145411448	**Clin. sign.:**	-	-	

Results

**Tool:**	**PredictSNP2**	**CADD**	**DANN**	**FATHMM**	**FunSeq2**	**GWAVA**
**Prediction:**	neutral	neutral	neutral	neutral	neutral	neutral
**Score:**	-1.0000	8.3340	0.7317	0.6058	0.0000	0.3700
**Exp. accuracy:**	0.96	0.92	0.91	0.69	0.93	0.56

External links

[dbSNP] [GenBank] [HaploReg] [RegulomeDB] [UCSC] [Ensemble]

**Variant:** 21 : 32664916, $T \rightarrow$C					
**Position:**	chr21:32664916	**Ref. allele:**	T	**Alt. allele:**	C
**Region:**	exonic	**Exonic. func.:**	synonymous	**Gene ID:**	SYNJ1
**RS number:**	rs138073731	**Clin. sign.:**	-	-	

Results

**Tool:**	**PredictSNP2**	**CADD**	**DANN**	**FATHMM**	**FunSeq2**	**GWAVA**
**Prediction:**	neutral	neutral	neutral	neutral	neutral	deleterious
**Score:**	-1.0000	1.8820	0.7093	0.2747	0.0000	0.5800
**Exp. accuracy:**	0.96	0.95	0.93	0.73	0.93	0.65

External links

[dbSNP] [GenBank] [HaploReg] [RegulomeDB] [UCSC] [Ensemble]

**Variant:** 21 : 32664922, $A \to G$					
**Position:**	chr21:32664922	**Ref. allele:**	A	**Alt. allele:**	G
**Region:**	exonic	**Exonic. func.:**	synonymous	**Gene ID:**	SYNJ1
**RS number:**	rs142455194	**Clin. sign.:**	-	-	

Results

**Tool:**	**PredictSNP2**	**CADD**	**DANN**	**FATHMM**	**FunSeq2**	**GWAVA**
**Prediction:**	neutral	neutral	neutral	deleterious	neutral	deleterious
**Score:**	-0.4994	7.0540	0.6901	0.9165	0.0000	0.6800
**Exp. accuracy:**	0.93	0.92	0.94	0.77	0.93	0.70

External links

[dbSNP] [GenBank] [HaploReg] [RegulomeDB] [UCSC] [Ensemble]

**Variant:** 21 : 32664961, $A \to G$					
**Position:**	chr21:32664961	**Ref. allele:**	A	**Alt. allele:**	G
**Region:**	exonic	**Exonic. func.:**	synonymous	**Gene ID:**	SYNJ1
**RS number:**	-	**Clin. sign.:**	-	-	

Results

**Tool:**	**PredictSNP2**	**CADD**	**DANN**	**FATHMM**	**FunSeq2**	**GWAVA**
**Prediction:**	neutral	neutral	neutral	deleterious	neutral	deleterious
**Score:**	-0.5394	8.3270	0.6005	0.8918	0.0000	0.7000
**Exp. accuracy:**	0.93	0.92	0.96	0.68	0.93	0.70

External links

[GenBank] [UCSC] [Ensemble]

**Variant:** 21 : 32664976, $T \to C$					
**Position:**	chr21:32664976	**Ref. allele:**	T	**Alt. allele:**	C
**Region:**	exonic	**Exonic. func.:**	synonymous	**Gene ID:**	SYNJ1
**RS number:**	-	**Clin. sign.:**	-	-	

Results

**Tool:**	**PredictSNP2**	**CADD**	**DANN**	**FATHMM**	**FunSeq2**	**GWAVA**
**Prediction:**	neutral	neutral	neutral	neutral	neutral	deleterious
**Score:**	-1.0000	6.0080	0.6599	0.3859	0.0000	0.6000
**Exp. accuracy:**	0.96	0.93	0.95	0.72	0.93	0.64

External links

[GenBank] [UCSC] [Ensemble]

**Variant:** 21 : 32664991, $A \rightarrow$T					
**Position:**	chr21:32664991	**Ref. allele:**	A	**Alt. allele:**	T
**Region:**	exonic	**Exonic. func.:**	synonymous	**Gene ID:**	SYNJ1
**RS number:**	-	**Clin. sign.:**	-	-	

Results

**Tool:**	**PredictSNP2**	**CADD**	**DANN**	**FATHMM**	**FunSeq2**	**GWAVA**
**Prediction:**	neutral	neutral	neutral	neutral	neutral	deleterious
**Score:**	-1.0000	0.8760	0.7049	0.5747	0.0000	0.6100
**Exp. accuracy:**	0.96	0.95	0.94	0.70	0.93	0.64

External links

[GenBank] [UCSC] [Ensemble]

**Variant:** 21 : 32665000, $G \rightarrow$A					
**Position:**	chr21:32665000	**Ref. allele:**	G	**Alt. allele:**	A
**Region:**	exonic	**Exonic. func.:**	synonymous	**Gene ID:**	SYNJ1
**RS number:**	-	**Clin. sign.:**	-	-	

Results

**Tool:**	**PredictSNP2**	**CADD**	**DANN**	**FATHMM**	**FunSeq2**	**GWAVA**
**Prediction:**	neutral	neutral	neutral	neutral	neutral	deleterious
**Score:**	-1.0000	9.1170	0.6179	0.3291	0.0000	0.6400
**Exp. accuracy:**	0.96	0.92	0.96	0.73	0.93	0.68

External links

[GenBank] [UCSC] [Ensemble]

**Variant:** 21 : 32665003, $A \rightarrow$G					
**Position:**	chr21:32665003	**Ref. allele:**	A	**Alt. allele:**	G
**Region:**	exonic	**Exonic. func.:**	synonymous	**Gene ID:**	SYNJ1
**RS number:**	-	**Clin. sign.:**	-	-	

Results

**Tool:**	**PredictSNP2**	**CADD**	**DANN**	**FATHMM**	**FunSeq2**	**GWAVA**
**Prediction:**	neutral	neutral	neutral	neutral	neutral	deleterious
**Score:**	-1.0000	7.6620	0.7626	0.5857	0.0000	0.6600
**Exp. accuracy:**	0.96	0.91	0.90	0.70	0.93	0.70

External links

[GenBank] [UCSC] [Ensemble]

**Variant:** 21 : 32665012, $G \to$ A					
**Position:**	chr21:32665012	**Ref. allele:**	G	**Alt. allele:**	A
**Region:**	exonic	**Exonic. func.:**	synonymous	**Gene ID:**	SYNJ1
**RS number:**	rs149236383	**Clin. sign.:**	-	-	

Results

**Tool:**	**PredictSNP2**	**CADD**	**DANN**	**FATHMM**	**FunSeq2**	**GWAVA**
**Prediction:**	neutral	neutral	neutral	neutral	neutral	deleterious
**Score:**	-1.0000	9.4040	0.7231	0.0698	0.0000	0.6600
**Exp. accuracy:**	0.96	0.91	0.93	0.90	0.93	0.70

External links

[dbSNP] [GenBank] [HaploReg] [RegulomeDB] [UCSC] [Ensemble]

**Variant:** 21 : 32665045, $A \to$ G					
**Position:**	chr21:32665045	**Ref. allele:**	A	**Alt. allele:**	G
**Region:**	exonic	**Exonic. func.:**	synonymous	**Gene ID:**	SYNJ1
**RS number:**	rs144454116	**Clin. sign.:**	-	-	

Results

**Tool:**	**PredictSNP2**	**CADD**	**DANN**	**FATHMM**	**FunSeq2**	**GWAVA**
**Prediction:**	neutral	neutral	neutral	neutral	neutral	deleterious
**Score:**	-1.0000	0.9100	0.4083	0.7634	0.0000	0.6500
**Exp. accuracy:**	0.96	0.95	0.97	0.69	0.93	0.70

External links

[dbSNP] [GenBank] [HaploReg] [RegulomeDB] [UCSC] [Ensemble]

**Variant:** 21 : 32665048, $G \to$ A					
**Position:**	chr21:32665048	**Ref. allele:**	G	**Alt. allele:**	A
**Region:**	exonic	**Exonic. func.:**	synonymous	**Gene ID:**	SYNJ1
**RS number:**	-	**Clin. sign.:**	-	-	

Results

**Tool:**	**PredictSNP2**	**CADD**	**DANN**	**FATHMM**	**FunSeq2**	**GWAVA**
**Prediction:**	neutral	neutral	neutral	deleterious	neutral	deleterious
**Score:**	-0.5965	8.0820	0.5314	0.8422	0.0000	0.7200
**Exp. accuracy:**	0.93	0.91	0.97	0.57	0.93	0.70

External links

[GenBank] [UCSC] [Ensemble]

**Variant:** 21 : 32665051, $A \rightarrow$G					
**Position:**	chr21:32665051	**Ref. allele:**	A	**Alt. allele:**	G
**Region:**	exonic	**Exonic. func.:**	synonymous	**Gene ID:**	SYNJ1
**RS number:**	rs148784824	**Clin. sign.:**	-	-	

Results

**Tool:**	**PredictSNP2**	**CADD**	**DANN**	**FATHMM**	**FunSeq2**	**GWAVA**
**Prediction:**	neutral	neutral	neutral	deleterious	neutral	deleterious
**Score:**	-0.5675	6.4820	0.5665	0.8723	0.0000	0.6100
**Exp. accuracy:**	0.93	0.93	0.97	0.60	0.93	0.64

External links

[dbSNP] [GenBank] [HaploReg] [RegulomeDB] [UCSC] [Ensemble]

**Variant:** 21 : 32665060, $T \rightarrow$C					
**Position:**	chr21:32665060	**Ref. allele:**	T	**Alt. allele:**	C
**Region:**	exonic	**Exonic. func.:**	synonymous	**Gene ID:**	SYNJ1
**RS number:**	rs112875942	**Clin. sign.:**	-	-	

Results

**Tool:**	**PredictSNP2**	**CADD**	**DANN**	**FATHMM**	**FunSeq2**	**GWAVA**
**Prediction:**	neutral	neutral	neutral	neutral	neutral	deleterious
**Score:**	-1.0000	4.5000	0.5580	0.0283	0.0000	0.5900
**Exp. accuracy:**	0.96	0.93	0.97	0.96	0.93	0.62

External links

[dbSNP] [GenBank] [HaploReg] [RegulomeDB] [UCSC] [Ensemble]

**Variant:** 21 : 32665946, $A \rightarrow$C					
**Position:**	chr21:32665946	**Ref. allele:**	A	**Alt. allele:**	C
**Region:**	exonic	**Exonic. func.:**	synonymous	**Gene ID:**	SYNJ1
**RS number:**	-	**Clin. sign.:**	-	-	

Results

**Tool:**	**PredictSNP2**	**CADD**	**DANN**	**FATHMM**	**FunSeq2**	**GWAVA**
**Prediction:**	neutral	neutral	neutral	neutral	neutral	neutral
**Score:**	-1.0000	0.0800	0.7140	0.2582	0.0000	0.3700
**Exp. accuracy:**	0.96	0.95	0.93	0.73	0.93	0.56

External links

[GenBank] [UCSC] [Ensemble]

PredictSNP overview

Date: 2017/03/06
Genome version: GRCh38/hg38
PredictSNP version: 2.1

If you use PredictSNP and obtain scientific results that you publish, we would ask you to acknowledge the usage of PredictSNP by referencing the article below.

Bendl, J., Musil, M., Stourac, J., Zendulka, J., Damborsky, J., Brezovsky, J., 2016: PredictSNP2: A unified platform for accurately evaluating SNP effects by exploiting the different characteristics of variants in distinct genomic regions. *PLOS Computational Biology* 12: e1004962

	PredictSNP2		CADD		DANN		FATHMM		FunSeq2		GWAVA	
	Neu	Del	Neu	Del	Neu	Del	Neu	Del	Neu	Del	Neu	Del
Regulatory	0	0	0	0	0	0	0	0	0	0	0	0
Splicing	0	0	0	0	0	0	0	0	0	0	0	0
Missense	0	0	0	0	0	0	92	3	80	15	92	3
Synonymous	71	24	95	0	38	56	0	0	0	0	0	0
Nonsense	0	0	0	0	0	0	0	0	0	0	0	0
Others	0	0	0	0	0	0	0	0	0	0	0	0

**Variant:** 21 : 32666033, $G \rightarrow$A					
**Position:**	chr21:32666033	**Ref. allele:**	G	**Alt. allele:**	A
**Region:**	exonic	**Exonic. func.:**	synonymous	**Gene ID:**	SYNJ1
**RS number:**	-	**Clin. sign.:**	-	-	

Results

**Tool:**	**PredictSNP2**	**CADD**	**DANN**	**FATHMM**	**FunSeq2**	**GWAVA**
**Prediction:**	neutral	neutral	neutral	neutral	neutral	neutral
**Score:**	-1.0000	10.3600	0.6301	0.2944	0.0000	0.3600
**Exp. accuracy:**	0.96	0.88	0.97	0.73	0.93	0.55

External links

[GenBank] [UCSC] [Ensemble]

**Variant:** 21 : 32666045, $G \rightarrow$C					
**Position:**	chr21:32666045	**Ref. allele:**	G	**Alt. allele:**	C
**Region:**	exonic	**Exonic. func.:**	synonymous	**Gene ID:**	SYNJ1
**RS number:**	-	**Clin. sign.:**	-	-	

Results

**Tool:**	**PredictSNP2**	**CADD**	**DANN**	**FATHMM**	**FunSeq2**	**GWAVA**
**Prediction:**	neutral	neutral	neutral	deleterious	neutral	neutral
**Score:**	-0.5271	6.6890	0.7187	0.8901	0.0000	0.3900
**Exp. accuracy:**	0.93	0.92	0.93	0.68	0.93	0.52

External links

[GenBank] [UCSC] [Ensemble]

**Variant:** 21 : 32666054, $G \rightarrow$A					
**Position:**	chr21:32666054	**Ref. allele:**	G	**Alt. allele:**	A
**Region:**	exonic	**Exonic. func.:**	synonymous	**Gene ID:**	SYNJ1
**RS number:**	rs116024329	**Clin. sign.:**	-	-	

Results

**Tool:**	**PredictSNP2**	**CADD**	**DANN**	**FATHMM**	**FunSeq2**	**GWAVA**
**Prediction:**	neutral	neutral	neutral	deleterious	neutral	deleterious
**Score:**	-0.5206	10.3900	0.7098	0.8970	0.0000	0.5400
**Exp. accuracy:**	0.93	0.88	0.93	0.71	0.93	0.62

External links

[dbSNP] [GenBank] [HaploReg] [RegulomeDB] [UCSC] [Ensemble]

Variant: 21 : 32666117, $A \to G$					
**Position:**	chr21:32666117	**Ref. allele:**	A	**Alt. allele:**	G
**Region:**	exonic	**Exonic. func.:**	synonymous	**Gene ID:**	SYNJ1
**RS number:**	-	**Clin. sign.:**	-	-	

Results

**Tool:**	**PredictSNP2**	**CADD**	**DANN**	**FATHMM**	**FunSeq2**	**GWAVA**
**Prediction:**	neutral	neutral	neutral	neutral	neutral	neutral
**Score:**	-1.0000	0.0990	0.6204	0.2256	0.0000	0.3900
**Exp. accuracy:**	0.96	0.95	0.96	0.73	0.93	0.52

External links

[GenBank] [UCSC] [Ensemble]

Variant: 21 : 32666120, $A \to G$					
**Position:**	chr21:32666120	**Ref. allele:**	A	**Alt. allele:**	G
**Region:**	exonic	**Exonic. func.:**	synonymous	**Gene ID:**	SYNJ1
**RS number:**	rs140112176	**Clin. sign.:**	-	-	

Results

**Tool:**	**PredictSNP2**	**CADD**	**DANN**	**FATHMM**	**FunSeq2**	**GWAVA**
**Prediction:**	neutral	neutral	neutral	neutral	neutral	neutral
**Score:**	-1.0000	9.1980	0.5913	0.7713	0.0000	0.4000
**Exp. accuracy:**	0.96	0.92	0.96	0.69	0.93	0.56

External links

[dbSNP] [GenBank] [HaploReg] [RegulomeDB] [UCSC] [Ensemble]

Variant: 21 : 32666129, $A \to C$					
**Position:**	chr21:32666129	**Ref. allele:**	A	**Alt. allele:**	C
**Region:**	exonic	**Exonic. func.:**	synonymous	**Gene ID:**	SYNJ1
**RS number:**	-	**Clin. sign.:**	-	-	

Results

**Tool:**	**PredictSNP2**	**CADD**	**DANN**	**FATHMM**	**FunSeq2**	**GWAVA**
**Prediction:**	neutral	neutral	neutral	deleterious	neutral	neutral
**Score:**	-0.5846	1.9540	0.6813	0.8254	0.0000	0.3500
**Exp. accuracy:**	0.93	0.95	0.94	0.57	0.93	0.54

External links

[GenBank] [UCSC] [Ensemble]

**Variant:** 21 : 32666135, $C \rightarrow$T					
**Position:**	chr21:32666135	**Ref. allele:**	C	**Alt. allele:**	T
**Region:**	exonic	**Exonic. func.:**	synonymous	**Gene ID:**	SYNJ1
**RS number:**	-	**Clin. sign.:**	-	-	

Results

**Tool:**	**PredictSNP2**	**CADD**	**DANN**	**FATHMM**	**FunSeq2**	**GWAVA**
**Prediction:**	deleterious	deleterious	deleterious	deleterious	neutral	deleterious
**Score:**	0.4739	17.8800	0.9175	0.9194	0.0000	0.5800
**Exp. accuracy:**	0.93	0.56	0.87	0.78	0.93	0.65

External links

[GenBank] [UCSC] [Ensemble]

**Variant:** 21 : 32666447, $A \rightarrow$G					
**Position:**	chr21:32666447	**Ref. allele:**	A	**Alt. allele:**	G
**Region:**	exonic	**Exonic. func.:**	synonymous	**Gene ID:**	SYNJ1
**RS number:**	rs116801038	**Clin. sign.:**	-	-	

Results

**Tool:**	**PredictSNP2**	**CADD**	**DANN**	**FATHMM**	**FunSeq2**	**GWAVA**
**Prediction:**	neutral	neutral	neutral	neutral	neutral	deleterious
**Score:**	-1.0000	6.4180	0.6744	0.5351	0.0000	0.6000
**Exp. accuracy:**	0.96	0.93	0.94	0.70	0.93	0.64

External links

[dbSNP] [GenBank] [HaploReg] [RegulomeDB] [UCSC] [Ensemble]

**Variant:** 21 : 32666465, $A \rightarrow$G					
**Position:**	chr21:32666465	**Ref. allele:**	A	**Alt. allele:**	G
**Region:**	exonic	**Exonic. func.:**	synonymous	**Gene ID:**	SYNJ1
**RS number:**	-	**Clin. sign.:**	-	-	

Results

**Tool:**	**PredictSNP2**	**CADD**	**DANN**	**FATHMM**	**FunSeq2**	**GWAVA**
**Prediction:**	neutral	neutral	neutral	neutral	neutral	deleterious
**Score:**	-1.0000	0.4020	0.6873	0.6449	0.0000	0.6000
**Exp. accuracy:**	0.96	0.95	0.94	0.69	0.93	0.64

External links

[GenBank] [UCSC] [Ensemble]

**Variant:** 21 : 32666471, $C \rightarrow$T					
**Position:**	chr21:32666471	**Ref. allele:**	C	**Alt. allele:**	T
**Region:**	exonic	**Exonic. func.:**	synonymous	**Gene ID:**	SYNJ1
**RS number:**	-	**Clin. sign.:**	-	-	

Results

**Tool:**	**PredictSNP2**	**CADD**	**DANN**	**FATHMM**	**FunSeq2**	**GWAVA**
**Prediction:**	neutral	neutral	neutral	deleterious	neutral	deleterious
**Score:**	-0.5705	12.7700	0.7538	0.8007	0.0000	0.6500
**Exp. accuracy:**	0.93	0.77	0.90	0.57	0.93	0.70

External links

[GenBank] [UCSC] [Ensemble]

**Variant:** 21 : 32666480, $G \rightarrow$A					
**Position:**	chr21:32666480	**Ref. allele:**	G	**Alt. allele:**	A
**Region:**	exonic	**Exonic. func.:**	synonymous	**Gene ID:**	SYNJ1
**RS number:**	-	**Clin. sign.:**	-	-	

Results

**Tool:**	**PredictSNP2**	**CADD**	**DANN**	**FATHMM**	**FunSeq2**	**GWAVA**
**Prediction:**	neutral	neutral	neutral	neutral	neutral	deleterious
**Score:**	-1.0000	15.0200	0.6909	0.4826	0.0000	0.6800
**Exp. accuracy:**	0.96	0.73	0.94	0.72	0.93	0.70

External links

[GenBank] [UCSC] [Ensemble]

**Variant:** 21 : 32666498, $A \rightarrow$G					
**Position:**	chr21:32666498	**Ref. allele:**	A	**Alt. allele:**	G
**Region:**	exonic	**Exonic. func.:**	synonymous	**Gene ID:**	SYNJ1
**RS number:**	-	**Clin. sign.:**	-	-	

Results

**Tool:**	**PredictSNP2**	**CADD**	**DANN**	**FATHMM**	**FunSeq2**	**GWAVA**
**Prediction:**	neutral	neutral	neutral	neutral	neutral	deleterious
**Score:**	-1.0000	8.6180	0.6635	0.2106	0.0000	0.5900
**Exp. accuracy:**	0.96	0.92	0.95	0.77	0.93	0.62

External links

[GenBank] [UCSC] [Ensemble]

**Variant:** 21 : 32666555, $G \rightarrow$C					
**Position:**	chr21:32666555	**Ref. allele:**	G	**Alt. allele:**	C
**Region:**	exonic	**Exonic. func.:**	synonymous	**Gene ID:**	SYNJ1
**RS number:**	rs115658799	**Clin. sign.:**	-	-	

Results

**Tool:**	**PredictSNP2**	**CADD**	**DANN**	**FATHMM**	**FunSeq2**	**GWAVA**
**Prediction:**	neutral	neutral	neutral	neutral	neutral	deleterious
**Score:**	-1.0000	7.9110	0.7381	0.6668	0.0000	0.6800
**Exp. accuracy:**	0.96	0.91	0.91	0.69	0.93	0.70

External links

[dbSNP] [GenBank] [HaploReg] [RegulomeDB] [UCSC] [Ensemble]

**Variant:** 21 : 32666561, $C \rightarrow$T					
**Position:**	chr21:32666561	**Ref. allele:**	C	**Alt. allele:**	T
**Region:**	exonic	**Exonic. func.:**	synonymous	**Gene ID:**	SYNJ1
**RS number:**	rs371628726	**Clin. sign.:**	-	-	

Results

**Tool:**	**PredictSNP2**	**CADD**	**DANN**	**FATHMM**	**FunSeq2**	**GWAVA**
**Prediction:**	neutral	neutral	neutral	deleterious	neutral	deleterious
**Score:**	-0.5572	12.5900	0.6576	0.8680	0.0000	0.7500
**Exp. accuracy:**	0.93	0.79	0.95	0.60	0.93	0.70

External links

[dbSNP] [GenBank] [RegulomeDB] [UCSC] [Ensemble]

**Variant:** 21 : 32670371, $A \rightarrow$G					
**Position:**	chr21:32670371	**Ref. allele:**	A	**Alt. allele:**	G
**Region:**	exonic	**Exonic. func.:**	synonymous	**Gene ID:**	SYNJ1
**RS number:**	-	**Clin. sign.:**	-	-	

Results

**Tool:**	**PredictSNP2**	**CADD**	**DANN**	**FATHMM**	**FunSeq2**	**GWAVA**
**Prediction:**	neutral	neutral	neutral	deleterious	neutral	deleterious
**Score:**	-0.5011	12.8100	0.8291	0.8824	0.0000	0.6000
**Exp. accuracy:**	0.93	0.77	0.90	0.64	0.93	0.64

External links

[GenBank] [UCSC] [Ensemble]

**Variant:** 21 : 32673398, $C \to$T					
**Position:**	chr21:32673398	**Ref. allele:**	C	**Alt. allele:**	T
**Region:**	exonic	**Exonic. func.:**	synonymous	**Gene ID:**	SYNJ1
**RS number:**	-	**Clin. sign.:**	-	-	

Results

**Tool:**	**PredictSNP2**	**CADD**	**DANN**	**FATHMM**	**FunSeq2**	**GWAVA**
**Prediction:**	neutral	neutral	neutral	deleterious	neutral	deleterious
**Score:**	-0.5778	12.4200	0.6952	0.8289	0.0000	0.7400
**Exp. accuracy:**	0.93	0.79	0.94	0.57	0.93	0.70

External links

[GenBank] [UCSC] [Ensemble]

**Variant:** 21 : 32673425, $T \to$C					
**Position:**	chr21:32673425	**Ref. allele:**	T	**Alt. allele:**	C
**Region:**	exonic	**Exonic. func.:**	synonymous	**Gene ID:**	SYNJ1
**RS number:**	-	**Clin. sign.:**	-	-	

Results

**Tool:**	**PredictSNP2**	**CADD**	**DANN**	**FATHMM**	**FunSeq2**	**GWAVA**
**Prediction:**	neutral	neutral	neutral	neutral	neutral	deleterious
**Score:**	-1.0000	1.2620	0.3748	0.3714	0.0000	0.6500
**Exp. accuracy:**	0.96	0.95	0.97	0.72	0.93	0.70

External links

[GenBank] [UCSC] [Ensemble]

**Variant:** 21 : 32673437, $A \to$G					
**Position:**	chr21:32673437	**Ref. allele:**	A	**Alt. allele:**	G
**Region:**	exonic	**Exonic. func.:**	synonymous	**Gene ID:**	SYNJ1
**RS number:**	-	**Clin. sign.:**	-	-	

Results

**Tool:**	**PredictSNP2**	**CADD**	**DANN**	**FATHMM**	**FunSeq2**	**GWAVA**
**Prediction:**	neutral	neutral	neutral	neutral	neutral	deleterious
**Score:**	-1.0000	7.0930	0.5946	0.1078	0.0000	0.6200
**Exp. accuracy:**	0.96	0.92	0.96	0.87	0.93	0.66

External links

[GenBank] [UCSC] [Ensemble]

**Variant:** 21 : 32673455, $G \to$ A					
**Position:**	chr21:32673455	**Ref. allele:**	G	**Alt. allele:**	A
**Region:**	exonic	**Exonic. func.:**	synonymous	**Gene ID:**	SYNJ1
**RS number:**	-	**Clin. sign.:**	-	-	

Results

**Tool:**	**PredictSNP2**	**CADD**	**DANN**	**FATHMM**	**FunSeq2**	**GWAVA**
**Prediction:**	neutral	neutral	neutral	neutral	neutral	deleterious
**Score:**	-1.0000	10.7800	0.6253	0.0638	0.0000	0.6000
**Exp. accuracy:**	0.96	0.88	0.96	0.92	0.93	0.64

External links

[GenBank] [UCSC] [Ensemble]

**Variant:** 21 : 32673455, $G \to$ C					
**Position:**	chr21:32673455	**Ref. allele:**	G	**Alt. allele:**	C
**Region:**	exonic	**Exonic. func.:**	synonymous	**Gene ID:**	SYNJ1
**RS number:**	rs150180190	**Clin. sign.:**	-	-	

Results

**Tool:**	**PredictSNP2**	**CADD**	**DANN**	**FATHMM**	**FunSeq2**	**GWAVA**
**Prediction:**	neutral	neutral	neutral	neutral	neutral	deleterious
**Score:**	-1.0000	9.3760	0.6443	0.1476	0.0000	0.6000
**Exp. accuracy:**	0.96	0.91	0.96	0.83	0.93	0.64

External links

[dbSNP] [GenBank] [HaploReg] [RegulomeDB] [UCSC] [Ensemble]

**Variant:** 21 : 32673470, $T \to$ C					
**Position:**	chr21:32673470	**Ref. allele:**	T	**Alt. allele:**	C
**Region:**	exonic	**Exonic. func.:**	synonymous	**Gene ID:**	SYNJ1
**RS number:**	rs111686118	**Clin. sign.:**	-	-	

Results

**Tool:**	**PredictSNP2**	**CADD**	**DANN**	**FATHMM**	**FunSeq2**	**GWAVA**
**Prediction:**	neutral	neutral	neutral	neutral	neutral	deleterious
**Score:**	-1.0000	7.8290	0.6601	0.2352	0.0000	0.5800
**Exp. accuracy:**	0.96	0.91	0.95	0.73	0.93	0.65

External links

[dbSNP] [GenBank] [HaploReg] [RegulomeDB] [UCSC] [Ensemble]

**Variant:** 21 : 32673485, $A \rightarrow$G					
**Position:**	chr21:32673485	**Ref. allele:**	A	**Alt. allele:**	G
**Region:**	exonic	**Exonic. func.:**	synonymous	**Gene ID:**	SYNJ1
**RS number:**	-	**Clin. sign.:**	-	-	

Results

**Tool:**	**PredictSNP2**	**CADD**	**DANN**	**FATHMM**	**FunSeq2**	**GWAVA**
**Prediction:**	neutral	neutral	neutral	neutral	neutral	deleterious
**Score:**	-1.0000	4.0460	0.4159	0.5720	0.0000	0.6600
**Exp. accuracy:**	0.96	0.94	0.97	0.70	0.93	0.70

External links

[GenBank] [UCSC] [Ensemble]

**Variant:** 21 : 32673503, $A \rightarrow$G					
**Position:**	chr21:32673503	**Ref. allele:**	A	**Alt. allele:**	G
**Region:**	exonic	**Exonic. func.:**	synonymous	**Gene ID:**	SYNJ1
**RS number:**	-	**Clin. sign.:**	-	-	

Results

**Tool:**	**PredictSNP2**	**CADD**	**DANN**	**FATHMM**	**FunSeq2**	**GWAVA**
**Prediction:**	neutral	neutral	neutral	deleterious	neutral	deleterious
**Score:**	-0.4825	7.9160	0.6606	0.9304	0.0000	0.7500
**Exp. accuracy:**	0.93	0.91	0.95	0.82	0.93	0.70

External links

[GenBank] [UCSC] [Ensemble]

**Variant:** 21 : 32678652, $A \rightarrow$G					
**Position:**	chr21:32678652	**Ref. allele:**	A	**Alt. allele:**	G
**Region:**	exonic	**Exonic. func.:**	synonymous	**Gene ID:**	SYNJ1
**RS number:**	-	**Clin. sign.:**	-	-	

Results

**Tool:**	**PredictSNP2**	**CADD**	**DANN**	**FATHMM**	**FunSeq2**	**GWAVA**
**Prediction:**	neutral	neutral	neutral	deleterious	neutral	deleterious
**Score:**	-0.5021	5.4020	0.7042	0.9121	0.0000	0.6000
**Exp. accuracy:**	0.93	0.92	0.94	0.77	0.93	0.64

External links

[GenBank] [UCSC] [Ensemble]

**Variant:**	21 : 32678697, $A \to$G				
**Position:**	chr21:32678697	**Ref. allele:**	A	**Alt. allele:**	G
**Region:**	exonic	**Exonic. func.:**	synonymous	**Gene ID:**	SYNJ1
**RS number:**	-	**Clin. sign.:**	-	-	

Results

**Tool:**	**PredictSNP2**	**CADD**	**DANN**	**FATHMM**	**FunSeq2**	**GWAVA**
**Prediction:**	neutral	neutral	neutral	neutral	neutral	deleterious
**Score:**	-1.0000	7.6050	0.6239	0.7269	0.0000	0.6300
**Exp. accuracy:**	0.96	0.91	0.96	0.69	0.93	0.66

External links

[GenBank] [UCSC] [Ensemble]

**Variant:**	21 : 32678714, $G \to$A				
**Position:**	chr21:32678714	**Ref. allele:**	G	**Alt. allele:**	A
**Region:**	exonic	**Exonic. func.:**	synonymous	**Gene ID:**	SYNJ1
**RS number:**	-	**Clin. sign.:**	-	-	

Results

**Tool:**	**PredictSNP2**	**CADD**	**DANN**	**FATHMM**	**FunSeq2**	**GWAVA**
**Prediction:**	neutral	neutral	neutral	deleterious	neutral	deleterious
**Score:**	-0.5351	9.6110	0.7255	0.8823	0.0000	0.7300
**Exp. accuracy:**	0.93	0.91	0.93	0.64	0.93	0.70

External links

[GenBank] [UCSC] [Ensemble]

**Variant:**	21 : 32678763, $C \to$T				
**Position:**	chr21:32678763	**Ref. allele:**	C	**Alt. allele:**	T
**Region:**	exonic	**Exonic. func.:**	synonymous	**Gene ID:**	SYNJ1
**RS number:**	-	**Clin. sign.:**	-	-	

Results

**Tool:**	**PredictSNP2**	**CADD**	**DANN**	**FATHMM**	**FunSeq2**	**GWAVA**
**Prediction:**	neutral	neutral	neutral	deleterious	neutral	deleterious
**Score:**	-0.5805	12.7200	0.6315	0.8547	0.0000	0.7800
**Exp. accuracy:**	0.93	0.79	0.97	0.57	0.93	0.70

External links

[GenBank] [UCSC] [Ensemble]

**Variant:**	21 : 32681571, $C \rightarrow$T				
**Position:**	chr21:32681571	**Ref. allele:**	C	**Alt. allele:**	T
**Region:**	exonic	**Exonic. func.:**	synonymous	**Gene ID:**	SYNJ1
**RS number:**	-	**Clin. sign.:**	-	-	

Results

**Tool:**	**PredictSNP2**	**CADD**	**DANN**	**FATHMM**	**FunSeq2**	**GWAVA**
**Prediction:**	neutral	deleterious	neutral	neutral	neutral	neutral
**Score:**	-0.6228	16.0700	0.5418	0.2211	0.0000	0.4400
**Exp. accuracy:**	0.95	0.58	0.97	0.77	0.93	0.54

External links

[GenBank] [UCSC] [Ensemble]

**Variant:**	21 : 32681592, $A \rightarrow$C				
**Position:**	chr21:32681592	**Ref. allele:**	A	**Alt. allele:**	C
**Region:**	exonic	**Exonic. func.:**	synonymous	**Gene ID:**	SYNJ1
**RS number:**	rs76972835	**Clin. sign.:**	-	-	

Results

**Tool:**	**PredictSNP2**	**CADD**	**DANN**	**FATHMM**	**FunSeq2**	**GWAVA**
**Prediction:**	neutral	neutral	neutral	deleterious	neutral	deleterious
**Score:**	-0.5586	2.8150	0.6379	0.8776	0.0000	0.5300
**Exp. accuracy:**	0.93	0.95	0.97	0.64	0.93	0.64

External links

[dbSNP] [GenBank] [HaploReg] [RegulomeDB] [UCSC] [Ensemble]

**Variant:**	21 : 32681595, $C \rightarrow$G				
**Position:**	chr21:32681595	**Ref. allele:**	C	**Alt. allele:**	G
**Region:**	exonic	**Exonic. func.:**	synonymous	**Gene ID:**	SYNJ1
**RS number:**	-	**Clin. sign.:**	-	-	

Results

**Tool:**	**PredictSNP2**	**CADD**	**DANN**	**FATHMM**	**FunSeq2**	**GWAVA**
**Prediction:**	neutral	neutral	neutral	deleterious	neutral	deleterious
**Score:**	-0.5766	10.9400	0.7581	0.8501	0.0000	0.6500
**Exp. accuracy:**	0.93	0.88	0.90	0.57	0.93	0.70

External links

[GenBank] [UCSC] [Ensemble]

**Variant:** 21 : 32681600, $A \rightarrow$G					
**Position:**	chr21:32681600	**Ref. allele:**	A	**Alt. allele:**	G
**Region:**	exonic	**Exonic. func.:**	synonymous	**Gene ID:**	SYNJ1
**RS number:**	-	**Clin. sign.:**	-	-	

Results

**Tool:**	**PredictSNP2**	**CADD**	**DANN**	**FATHMM**	**FunSeq2**	**GWAVA**
**Prediction:**	neutral	neutral	neutral	neutral	neutral	?
**Score:**	-1.0000	9.8710	0.7252	0.0956	0.0000	0.4800
**Exp. accuracy:**	0.96	0.91	0.93	0.87	0.93	0.43

External links

[GenBank] [UCSC] [Ensemble]

**Variant:** 21 : 32681604, $A \rightarrow$G					
**Position:**	chr21:32681604	**Ref. allele:**	A	**Alt. allele:**	G
**Region:**	exonic	**Exonic. func.:**	synonymous	**Gene ID:**	SYNJ1
**RS number:**	rs111655347	**Clin. sign.:**	-	-	

Results

**Tool:**	**PredictSNP2**	**CADD**	**DANN**	**FATHMM**	**FunSeq2**	**GWAVA**
**Prediction:**	neutral	neutral	neutral	neutral	neutral	neutral
**Score:**	-1.0000	9.6770	0.7661	0.7521	0.0000	0.4700
**Exp. accuracy:**	0.96	0.91	0.90	0.69	0.93	0.54

External links

[dbSNP] [GenBank] [HaploReg] [RegulomeDB] [UCSC] [Ensemble]

**Variant:** 21 : 32681607, $C \rightarrow$T					
**Position:**	chr21:32681607	**Ref. allele:**	C	**Alt. allele:**	T
**Region:**	exonic	**Exonic. func.:**	synonymous	**Gene ID:**	SYNJ1
**RS number:**	-	**Clin. sign.:**	-	-	

Results

**Tool:**	**PredictSNP2**	**CADD**	**DANN**	**FATHMM**	**FunSeq2**	**GWAVA**
**Prediction:**	neutral	neutral	neutral	neutral	neutral	deleterious
**Score:**	-1.0000	14.2500	0.6322	0.6506	0.0000	0.5200
**Exp. accuracy:**	0.96	0.73	0.97	0.69	0.93	0.58

External links

[GenBank] [UCSC] [Ensemble]

**Variant:** 21 : 32684044, $G \rightarrow$A					
**Position:**	chr21:32684044	**Ref. allele:**	G	**Alt. allele:**	A
**Region:**	exonic	**Exonic. func.:**	synonymous	**Gene ID:**	SYNJ1
**RS number:**	-	**Clin. sign.:**	-	-	

Results

**Tool:**	**PredictSNP2**	**CADD**	**DANN**	**FATHMM**	**FunSeq2**	**GWAVA**
**Prediction:**	neutral	neutral	neutral	deleterious	neutral	deleterious
**Score:**	-0.5729	13.2800	0.6937	0.8234	0.0000	0.5900
**Exp. accuracy:**	0.93	0.74	0.94	0.57	0.93	0.62

External links

[GenBank] [UCSC] [Ensemble]

**Variant:** 21 : 32684056, $C \rightarrow$T					
**Position:**	chr21:32684056	**Ref. allele:**	C	**Alt. allele:**	T
**Region:**	exonic	**Exonic. func.:**	synonymous	**Gene ID:**	SYNJ1
**RS number:**	-	**Clin. sign.:**	-	-	

Results

**Tool:**	**PredictSNP2**	**CADD**	**DANN**	**FATHMM**	**FunSeq2**	**GWAVA**
**Prediction:**	neutral	neutral	neutral	neutral	neutral	deleterious
**Score:**	-1.0000	13.7100	0.6221	0.7637	0.0000	0.7300
**Exp. accuracy:**	0.96	0.73	0.96	0.69	0.93	0.70

External links

[GenBank] [UCSC] [Ensemble]

**Variant:** 21 : 32684062, $A \rightarrow$G					
**Position:**	chr21:32684062	**Ref. allele:**	A	**Alt. allele:**	G
**Region:**	exonic	**Exonic. func.:**	synonymous	**Gene ID:**	SYNJ1
**RS number:**	-	**Clin. sign.:**	-	-	

Results

**Tool:**	**PredictSNP2**	**CADD**	**DANN**	**FATHMM**	**FunSeq2**	**GWAVA**
**Prediction:**	neutral	neutral	neutral	neutral	neutral	deleterious
**Score:**	-1.0000	2.9090	0.7608	0.6969	0.0000	0.5600
**Exp. accuracy:**	0.96	0.95	0.90	0.69	0.93	0.69

External links

[GenBank] [UCSC] [Ensemble]

**Variant:** 21 : 32684071, $T \to$C					
**Position:**	chr21:32684071	**Ref. allele:**	T	**Alt. allele:**	C
**Region:**	exonic	**Exonic. func.:**	synonymous	**Gene ID:**	SYNJ1
**RS number:**	-	**Clin. sign.:**	-	-	

Results

**Tool:**	**PredictSNP2**	**CADD**	**DANN**	**FATHMM**	**FunSeq2**	**GWAVA**
**Prediction:**	neutral	neutral	neutral	neutral	neutral	deleterious
**Score:**	-1.0000	5.5050	0.6601	0.7866	0.0000	0.6100
**Exp. accuracy:**	0.96	0.92	0.95	0.69	0.93	0.64

External links

[GenBank] [UCSC] [Ensemble]

**Variant:** 21 : 32685759, $A \to$G					
**Position:**	chr21:32685759	**Ref. allele:**	A	**Alt. allele:**	G
**Region:**	exonic	**Exonic. func.:**	synonymous	**Gene ID:**	SYNJ1
**RS number:**	-	**Clin. sign.:**	-	-	

Results

**Tool:**	**PredictSNP2**	**CADD**	**DANN**	**FATHMM**	**FunSeq2**	**GWAVA**
**Prediction:**	neutral	neutral	neutral	neutral	neutral	deleterious
**Score:**	-1.0000	4.9380	0.7161	0.6459	0.0000	0.7900
**Exp. accuracy:**	0.96	0.93	0.93	0.69	0.93	0.70

External links

[GenBank] [UCSC] [Ensemble]

**Variant:** 21 : 32685765, $A \to$G					
**Position:**	chr21:32685765	**Ref. allele:**	A	**Alt. allele:**	G
**Region:**	exonic	**Exonic. func.:**	synonymous	**Gene ID:**	SYNJ1
**RS number:**	-	**Clin. sign.:**	-	-	

Results

**Tool:**	**PredictSNP2**	**CADD**	**DANN**	**FATHMM**	**FunSeq2**	**GWAVA**
**Prediction:**	neutral	neutral	neutral	deleterious	neutral	deleterious
**Score:**	-0.5822	6.6240	0.7146	0.8215	0.0000	0.7700
**Exp. accuracy:**	0.93	0.92	0.93	0.57	0.93	0.70

External links

[GenBank] [UCSC] [Ensemble]

**Variant:** 21 : 32685789, $T \rightarrow$G					
**Position:**	chr21:32685789	**Ref. allele:**	T	**Alt. allele:**	G
**Region:**	exonic	**Exonic. func.:**	synonymous	**Gene ID:**	SYNJ1
**RS number:**	-	**Clin. sign.:**	-	-	

Results

**Tool:**	**PredictSNP2**	**CADD**	**DANN**	**FATHMM**	**FunSeq2**	**GWAVA**
**Prediction:**	neutral	neutral	neutral	neutral	neutral	deleterious
**Score:**	-1.0000	10.1700	0.7884	0.7722	0.0000	0.6400
**Exp. accuracy:**	0.96	0.88	0.89	0.69	0.93	0.68

External links

[GenBank] [UCSC] [Ensemble]

**Variant:** 21 : 32685864, $G \rightarrow$A					
**Position:**	chr21:32685864	**Ref. allele:**	G	**Alt. allele:**	A
**Region:**	exonic	**Exonic. func.:**	synonymous	**Gene ID:**	SYNJ1
**RS number:**	-	**Clin. sign.:**	-	-	

Results

**Tool:**	**PredictSNP2**	**CADD**	**DANN**	**FATHMM**	**FunSeq2**	**GWAVA**
**Prediction:**	neutral	neutral	neutral	deleterious	neutral	deleterious
**Score:**	-0.4762	8.3670	0.5476	0.9518	0.0000	0.7800
**Exp. accuracy:**	0.93	0.92	0.97	0.92	0.93	0.70

External links

[GenBank] [UCSC] [Ensemble]

**Variant:** 21 : 32685885, $A \rightarrow$G					
**Position:**	chr21:32685885	**Ref. allele:**	A	**Alt. allele:**	G
**Region:**	exonic	**Exonic. func.:**	synonymous	**Gene ID:**	SYNJ1
**RS number:**	-	**Clin. sign.:**	-	-	

Results

**Tool:**	**PredictSNP2**	**CADD**	**DANN**	**FATHMM**	**FunSeq2**	**GWAVA**
**Prediction:**	neutral	neutral	neutral	neutral	neutral	deleterious
**Score:**	-1.0000	4.8840	0.5681	0.7710	0.0000	0.6500
**Exp. accuracy:**	0.96	0.93	0.97	0.69	0.93	0.70

External links

[GenBank] [UCSC] [Ensemble]

**Variant:**	21 : 32687017, $T \to$C				
**Position:**	chr21:32687017	**Ref. allele:**	T	**Alt. allele:**	C
**Region:**	exonic	**Exonic. func.:**	synonymous	**Gene ID:**	SYNJ1
**RS number:**	rs145522240	**Clin. sign.:**	-	-	

Results

**Tool:**	**PredictSNP2**	**CADD**	**DANN**	**FATHMM**	**FunSeq2**	**GWAVA**
**Prediction:**	neutral	neutral	neutral	neutral	neutral	neutral
**Score:**	-1.0000	7.4870	0.6963	0.3840	0.0000	0.3300
**Exp. accuracy:**	0.96	0.91	0.94	0.72	0.93	0.56

External links

[dbSNP] [GenBank] [HaploReg] [RegulomeDB] [UCSC] [Ensemble]

**Variant:**	21 : 32687044, $A \to$G				
**Position:**	chr21:32687044	**Ref. allele:**	A	**Alt. allele:**	G
**Region:**	exonic	**Exonic. func.:**	synonymous	**Gene ID:**	SYNJ1
**RS number:**	-	**Clin. sign.:**	-	-	

Results

**Tool:**	**PredictSNP2**	**CADD**	**DANN**	**FATHMM**	**FunSeq2**	**GWAVA**
**Prediction:**	neutral	neutral	neutral	neutral	neutral	neutral
**Score:**	-1.0000	8.9340	0.6402	0.4990	0.0000	0.3700
**Exp. accuracy:**	0.96	0.92	0.97	0.70	0.93	0.56

External links

[GenBank] [UCSC] [Ensemble]

**Variant:**	21 : 32687062, $T \to$C				
**Position:**	chr21:32687062	**Ref. allele:**	T	**Alt. allele:**	C
**Region:**	exonic	**Exonic. func.:**	synonymous	**Gene ID:**	SYNJ1
**RS number:**	-	**Clin. sign.:**	-	-	

Results

**Tool:**	**PredictSNP2**	**CADD**	**DANN**	**FATHMM**	**FunSeq2**	**GWAVA**
**Prediction:**	neutral	neutral	neutral	neutral	neutral	neutral
**Score:**	-1.0000	5.6370	0.7936	0.6741	0.0000	0.3300
**Exp. accuracy:**	0.96	0.92	0.89	0.69	0.93	0.56

External links

[GenBank] [UCSC] [Ensemble]

**Variant:** 21 : 32688314, $A \rightarrow$G					
**Position:**	chr21:32688314	**Ref. allele:**	A	**Alt. allele:**	G
**Region:**	exonic	**Exonic. func.:**	synonymous	**Gene ID:**	SYNJ1
**RS number:**	rs372016074	**Clin. sign.:**	-	-	

Results

**Tool:**	**PredictSNP2**	**CADD**	**DANN**	**FATHMM**	**FunSeq2**	**GWAVA**
**Prediction:**	neutral	deleterious	neutral	neutral	neutral	deleterious
**Score:**	-0.5972	15.7400	0.7650	0.6625	0.0000	0.6700
**Exp. accuracy:**	0.93	0.58	0.90	0.69	0.93	0.70

External links

[dbSNP] [GenBank] [RegulomeDB] [UCSC] [Ensemble]

**Variant:** 21 : 32688320, $T \rightarrow$G					
**Position:**	chr21:32688320	**Ref. allele:**	T	**Alt. allele:**	G
**Region:**	exonic	**Exonic. func.:**	synonymous	**Gene ID:**	SYNJ1
**RS number:**	-	**Clin. sign.:**	-	-	

Results

**Tool:**	**PredictSNP2**	**CADD**	**DANN**	**FATHMM**	**FunSeq2**	**GWAVA**
**Prediction:**	neutral	deleterious	neutral	neutral	neutral	deleterious
**Score:**	-0.5913	17.1000	0.7985	0.6816	0.0000	0.7100
**Exp. accuracy:**	0.93	0.58	0.89	0.69	0.93	0.70

External links

[GenBank] [UCSC] [Ensemble]

**Variant:** 21 : 32688323, $A \rightarrow$G					
**Position:**	chr21:32688323	**Ref. allele:**	A	**Alt. allele:**	G
**Region:**	exonic	**Exonic. func.:**	synonymous	**Gene ID:**	SYNJ1
**RS number:**	-	**Clin. sign.:**	-	-	

Results

**Tool:**	**PredictSNP2**	**CADD**	**DANN**	**FATHMM**	**FunSeq2**	**GWAVA**
**Prediction:**	neutral	deleterious	neutral	neutral	neutral	deleterious
**Score:**	-0.5953	17.3400	0.7087	0.7305	0.0000	0.7000
**Exp. accuracy:**	0.93	0.58	0.93	0.69	0.93	0.70

External links

[GenBank] [UCSC] [Ensemble]

**Variant:** 21 : 32688326, $G \to$A					
**Position:**	chr21:32688326	**Ref. allele:**	G	**Alt. allele:**	A
**Region:**	exonic	**Exonic. func.:**	synonymous	**Gene ID:**	SYNJ1
**RS number:**	rs148732160	**Clin. sign.:**	-	-	

Results

**Tool:**	**PredictSNP2**	**CADD**	**DANN**	**FATHMM**	**FunSeq2**	**GWAVA**
**Prediction:**	neutral	neutral	neutral	neutral	neutral	deleterious
**Score:**	-1.0000	15.2800	0.8163	0.2542	0.0000	0.6100
**Exp. accuracy:**	0.96	0.73	0.90	0.73	0.93	0.64

External links

[dbSNP] [GenBank] [HaploReg] [RegulomeDB] [UCSC] [Ensemble]

**Variant:** 21 : 32694234, $C \to$T					
**Position:**	chr21:32694234	**Ref. allele:**	C	**Alt. allele:**	T
**Region:**	exonic	**Exonic. func.:**	synonymous	**Gene ID:**	SYNJ1
**RS number:**	rs188892266	**Clin. sign.:**	-	-	

Results

**Tool:**	**PredictSNP2**	**CADD**	**DANN**	**FATHMM**	**FunSeq2**	**GWAVA**
**Prediction:**	neutral	neutral	neutral	neutral	neutral	deleterious
**Score:**	-1.0000	13.6300	0.5668	0.0296	0.0000	0.5700
**Exp. accuracy:**	0.96	0.73	0.97	0.96	0.93	0.68

External links

[dbSNP] [GenBank] [HaploReg] [RegulomeDB] [UCSC] [Ensemble]

**Variant:** 21 : 32694258, $A \to$C					
**Position:**	chr21:32694258	**Ref. allele:**	A	**Alt. allele:**	C
**Region:**	exonic	**Exonic. func.:**	synonymous	**Gene ID:**	SYNJ1
**RS number:**	rs111290161	**Clin. sign.:**	-	-	

Results

**Tool:**	**PredictSNP2**	**CADD**	**DANN**	**FATHMM**	**FunSeq2**	**GWAVA**
**Prediction:**	neutral	neutral	neutral	neutral	neutral	deleterious
**Score:**	-1.0000	1.3760	0.6920	0.5849	0.0000	0.5700
**Exp. accuracy:**	0.96	0.95	0.94	0.70	0.93	0.68

External links

[dbSNP] [GenBank] [HaploReg] [RegulomeDB] [UCSC] [Ensemble]

Variant:	21 : 32694270, $G \rightarrow$A				
**Position:**	chr21:32694270	**Ref. allele:**	G	**Alt. allele:**	A
**Region:**	exonic	**Exonic. func.:**	synonymous	**Gene ID:**	SYNJ1
**RS number:**	-	**Clin. sign.:**	-	-	

Results

Tool:	PredictSNP2	CADD	DANN	FATHMM	FunSeq2	GWAVA
**Prediction:**	neutral	neutral	neutral	neutral	neutral	deleterious
**Score:**	-1.0000	9.0600	0.6930	0.5216	0.0000	0.6000
**Exp. accuracy:**	0.96	0.92	0.94	0.70	0.93	0.64

External links

[GenBank] [UCSC] [Ensemble]

Variant:	21 : 32694282, $G \rightarrow$T				
**Position:**	chr21:32694282	**Ref. allele:**	G	**Alt. allele:**	T
**Region:**	exonic	**Exonic. func.:**	synonymous	**Gene ID:**	SYNJ1
**RS number:**	-	**Clin. sign.:**	-	-	

Results

Tool:	PredictSNP2	CADD	DANN	FATHMM	FunSeq2	GWAVA
**Prediction:**	neutral	neutral	neutral	neutral	neutral	deleterious
**Score:**	-1.0000	7.3420	0.7499	0.6291	0.0000	0.5800
**Exp. accuracy:**	0.96	0.91	0.91	0.69	0.93	0.65

External links

[GenBank] [UCSC] [Ensemble]

Variant:	21 : 32694306, $C \rightarrow$T				
**Position:**	chr21:32694306	**Ref. allele:**	C	**Alt. allele:**	T
**Region:**	exonic	**Exonic. func.:**	synonymous	**Gene ID:**	SYNJ1
**RS number:**	-	**Clin. sign.:**	-	-	

Results

Tool:	PredictSNP2	CADD	DANN	FATHMM	FunSeq2	GWAVA
**Prediction:**	neutral	neutral	neutral	neutral	neutral	deleterious
**Score:**	-1.0000	10.0700	0.5729	0.0737	0.0000	0.5300
**Exp. accuracy:**	0.96	0.91	0.97	0.90	0.93	0.64

External links

[GenBank] [UCSC] [Ensemble]

**Variant:** 21 : 32695060, $T \rightarrow$C					
**Position:**	chr21:32695060	**Ref. allele:**	T	**Alt. allele:**	C
**Region:**	exonic	**Exonic. func.:**	synonymous	**Gene ID:**	SYNJ1
**RS number:**	rs372354458	**Clin. sign.:**	-	-	

Results

**Tool:**	**PredictSNP2**	**CADD**	**DANN**	**FATHMM**	**FunSeq2**	**GWAVA**
**Prediction:**	neutral	neutral	neutral	neutral	neutral	neutral
**Score:**	-1.0000	1.3350	0.6394	0.5486	0.0000	0.4600
**Exp. accuracy:**	0.96	0.95	0.97	0.70	0.93	0.54

External links

[dbSNP] [GenBank] [RegulomeDB] [UCSC] [Ensemble]

**Variant:** 21 : 32695078, $G \rightarrow$A					
**Position:**	chr21:32695078	**Ref. allele:**	G	**Alt. allele:**	A
**Region:**	exonic	**Exonic. func.:**	synonymous	**Gene ID:**	SYNJ1
**RS number:**	rs376985894	**Clin. sign.:**	-	-	

Results

**Tool:**	**PredictSNP2**	**CADD**	**DANN**	**FATHMM**	**FunSeq2**	**GWAVA**
**Prediction:**	neutral	neutral	neutral	neutral	neutral	deleterious
**Score:**	-1.0000	10.7200	0.6535	0.7199	0.0000	0.5900
**Exp. accuracy:**	0.96	0.88	0.96	0.69	0.93	0.62

External links

[dbSNP] [GenBank] [RegulomeDB] [UCSC] [Ensemble]

**Variant:** 21 : 32695153, $G \rightarrow$A					
**Position:**	chr21:32695153	**Ref. allele:**	G	**Alt. allele:**	A
**Region:**	exonic	**Exonic. func.:**	synonymous	**Gene ID:**	SYNJ1
**RS number:**	-	**Clin. sign.:**	-	-	

Results

**Tool:**	**PredictSNP2**	**CADD**	**DANN**	**FATHMM**	**FunSeq2**	**GWAVA**
**Prediction:**	neutral	neutral	neutral	deleterious	neutral	deleterious
**Score:**	-0.5542	10.4200	0.6845	0.8774	0.0000	0.6300
**Exp. accuracy:**	0.93	0.88	0.94	0.64	0.93	0.66

External links

[GenBank] [UCSC] [Ensemble]

**Variant:**	$21:32695165, C{\rightarrow}$T				
**Position:**	chr21:32695165	**Ref. allele:**	C	**Alt. allele:**	T
**Region:**	exonic	**Exonic. func.:**	synonymous	**Gene ID:**	SYNJ1
**RS number:**	-	**Clin. sign.:**	-	-	

Results

**Tool:**	**PredictSNP2**	**CADD**	**DANN**	**FATHMM**	**FunSeq2**	**GWAVA**
**Prediction:**	neutral	neutral	neutral	neutral	neutral	deleterious
**Score:**	-1.0000	15.2200	0.6383	0.3533	0.0000	0.5500
**Exp. accuracy:**	0.96	0.73	0.97	0.73	0.93	0.66

External links

[GenBank] [UCSC] [Ensemble]

**Variant:**	$21:32695189, T{\rightarrow}$C				
**Position:**	chr21:32695189	**Ref. allele:**	T	**Alt. allele:**	C
**Region:**	exonic	**Exonic. func.:**	synonymous	**Gene ID:**	SYNJ1
**RS number:**	rs61756694	**Clin. sign.:**	-	-	

Results

**Tool:**	**PredictSNP2**	**CADD**	**DANN**	**FATHMM**	**FunSeq2**	**GWAVA**
**Prediction:**	neutral	neutral	neutral	neutral	neutral	deleterious
**Score:**	-1.0000	0.3310	0.3817	0.7043	0.0000	0.6100
**Exp. accuracy:**	0.96	0.95	0.97	0.69	0.93	0.64

External links

[dbSNP] [GenBank] [HaploReg] [RegulomeDB] [UCSC] [Ensemble]

**Variant:**	$21:32695207, T{\rightarrow}$A				
**Position:**	chr21:32695207	**Ref. allele:**	T	**Alt. allele:**	A
**Region:**	exonic	**Exonic. func.:**	synonymous	**Gene ID:**	SYNJ1
**RS number:**	-	**Clin. sign.:**	-	-	

Results

**Tool:**	**PredictSNP2**	**CADD**	**DANN**	**FATHMM**	**FunSeq2**	**GWAVA**
**Prediction:**	neutral	neutral	neutral	neutral	neutral	deleterious
**Score:**	-1.0000	6.8870	0.6255	0.1308	0.0000	0.6000
**Exp. accuracy:**	0.96	0.92	0.96	0.85	0.93	0.64

External links

[GenBank] [UCSC] [Ensemble]

Variant: 21 : 32695273, $A \rightarrow$C					
**Position:**	chr21:32695273	**Ref. allele:**	A	**Alt. allele:**	C
**Region:**	exonic	**Exonic. func.:**	synonymous	**Gene ID:**	SYNJ1
**RS number:**	-	**Clin. sign.:**	-	-	

Results

**Tool:**	**PredictSNP2**	**CADD**	**DANN**	**FATHMM**	**FunSeq2**	**GWAVA**
**Prediction:**	neutral	neutral	neutral	neutral	neutral	deleterious
**Score:**	-1.0000	0.2470	0.7887	0.6499	0.0000	0.6000
**Exp. accuracy:**	0.96	0.95	0.89	0.69	0.93	0.64

External links

[GenBank] [UCSC] [Ensemble]

Variant: 21 : 32699849, $A \rightarrow$G					
**Position:**	chr21:32699849	**Ref. allele:**	A	**Alt. allele:**	G
**Region:**	exonic	**Exonic. func.:**	synonymous	**Gene ID:**	SYNJ1
**RS number:**	-	**Clin. sign.:**	-	-	

Results

**Tool:**	**PredictSNP2**	**CADD**	**DANN**	**FATHMM**	**FunSeq2**	**GWAVA**
**Prediction:**	neutral	neutral	neutral	neutral	neutral	neutral
**Score:**	-1.0000	7.3580	0.6219	0.5457	0.0000	0.2900
**Exp. accuracy:**	0.96	0.91	0.96	0.70	0.93	0.56

External links

[GenBank] [UCSC] [Ensemble]

Variant: 21 : 32699855, $A \rightarrow$T					
**Position:**	chr21:32699855	**Ref. allele:**	A	**Alt. allele:**	T
**Region:**	exonic	**Exonic. func.:**	synonymous	**Gene ID:**	SYNJ1
**RS number:**	rs369978981	**Clin. sign.:**	-	-	

Results

**Tool:**	**PredictSNP2**	**CADD**	**DANN**	**FATHMM**	**FunSeq2**	**GWAVA**
**Prediction:**	neutral	neutral	neutral	neutral	neutral	neutral
**Score:**	-1.0000	0.0030	0.5222	0.0331	0.0000	0.3100
**Exp. accuracy:**	0.96	0.95	0.97	0.96	0.93	0.53

External links

[dbSNP] [GenBank] [RegulomeDB] [UCSC] [Ensemble]

**Variant:** 21 : 32699864, $T \rightarrow$C					
**Position:**	chr21:32699864	**Ref. allele:**	T	**Alt. allele:**	C
**Region:**	exonic	**Exonic. func.:**	synonymous	**Gene ID:**	SYNJ1
**RS number:**	rs376163066	**Clin. sign.:**	-	-	

Results

**Tool:**	**PredictSNP2**	**CADD**	**DANN**	**FATHMM**	**FunSeq2**	**GWAVA**
**Prediction:**	neutral	neutral	neutral	neutral	neutral	neutral
**Score:**	-1.0000	0.7560	0.6044	0.2289	0.0000	0.3300
**Exp. accuracy:**	0.96	0.95	0.96	0.73	0.93	0.56

External links

[dbSNP] [GenBank] [RegulomeDB] [UCSC] [Ensemble]

**Variant:** 21 : 32699867, $T \rightarrow$C					
**Position:**	chr21:32699867	**Ref. allele:**	T	**Alt. allele:**	C
**Region:**	exonic	**Exonic. func.:**	synonymous	**Gene ID:**	SYNJ1
**RS number:**	-	**Clin. sign.:**	-	-	

Results

**Tool:**	**PredictSNP2**	**CADD**	**DANN**	**FATHMM**	**FunSeq2**	**GWAVA**
**Prediction:**	neutral	neutral	neutral	neutral	neutral	neutral
**Score:**	-1.0000	0.5510	0.4695	0.1922	0.0000	0.3300
**Exp. accuracy:**	0.96	0.95	0.97	0.80	0.93	0.56

External links

[GenBank] [UCSC] [Ensemble]

**Variant:** 21 : 32699882, $C \rightarrow$A					
**Position:**	chr21:32699882	**Ref. allele:**	C	**Alt. allele:**	A
**Region:**	exonic	**Exonic. func.:**	synonymous	**Gene ID:**	SYNJ1
**RS number:**	-	**Clin. sign.:**	-	-	

Results

**Tool:**	**PredictSNP2**	**CADD**	**DANN**	**FATHMM**	**FunSeq2**	**GWAVA**
**Prediction:**	neutral	deleterious	neutral	neutral	neutral	neutral
**Score:**	-0.6367	15.9500	0.6268	0.0692	0.0000	0.2900
**Exp. accuracy:**	0.95	0.58	0.97	0.90	0.93	0.56

External links

[GenBank] [UCSC] [Ensemble]

**Variant:** 21 : 32699882, $C \rightarrow$T					
**Position:**	chr21:32699882	**Ref. allele:**	C	**Alt. allele:**	T
**Region:**	exonic	**Exonic. func.:**	synonymous	**Gene ID:**	SYNJ1
**RS number:**	rs151248057	**Clin. sign.:**	-	-	

Results

**Tool:**	**PredictSNP2**	**CADD**	**DANN**	**FATHMM**	**FunSeq2**	**GWAVA**
**Prediction:**	neutral	neutral	neutral	neutral	neutral	neutral
**Score:**	-1.0000	14.9300	0.6617	0.0459	0.0000	0.2900
**Exp. accuracy:**	0.96	0.73	0.95	0.96	0.93	0.56

External links

[dbSNP] [GenBank] [HaploReg] [RegulomeDB] [UCSC] [Ensemble]

**Variant:** 21 : 32699896, $A \rightarrow$G					
**Position:**	chr21:32699896	**Ref. allele:**	A	**Alt. allele:**	G
**Region:**	exonic	**Exonic. func.:**	synonymous	**Gene ID:**	SYNJ1
**RS number:**	rs140461566	**Clin. sign.:**	-	-	

Results

**Tool:**	**PredictSNP2**	**CADD**	**DANN**	**FATHMM**	**FunSeq2**	**GWAVA**
**Prediction:**	neutral	neutral	neutral	neutral	neutral	neutral
**Score:**	-1.0000	2.8860	0.7027	0.3456	0.0000	0.3500
**Exp. accuracy:**	0.96	0.95	0.94	0.73	0.93	0.54

External links

[dbSNP] [GenBank] [HaploReg] [RegulomeDB] [UCSC] [Ensemble]

**Variant:** 21 : 32699912, $A \rightarrow$G					
**Position:**	chr21:32699912	**Ref. allele:**	A	**Alt. allele:**	G
**Region:**	exonic	**Exonic. func.:**	synonymous	**Gene ID:**	SYNJ1
**RS number:**	rs113672883	**Clin. sign.:**	-	-	

Results

**Tool:**	**PredictSNP2**	**CADD**	**DANN**	**FATHMM**	**FunSeq2**	**GWAVA**
**Prediction:**	neutral	neutral	neutral	neutral	neutral	neutral
**Score:**	-1.0000	8.7950	0.7482	0.7776	0.0000	0.3300
**Exp. accuracy:**	0.96	0.92	0.91	0.69	0.93	0.56

External links

[dbSNP] [GenBank] [HaploReg] [RegulomeDB] [UCSC] [Ensemble]

**Variant:**	21 : 32699924, $T \rightarrow$C				
**Position:**	chr21:32699924	**Ref. allele:**	T	**Alt. allele:**	C
**Region:**	exonic	**Exonic. func.:**	synonymous	**Gene ID:**	SYNJ1
**RS number:**	rs377218872	**Clin. sign.:**	-	-	

Results

**Tool:**	**PredictSNP2**	**CADD**	**DANN**	**FATHMM**	**FunSeq2**	**GWAVA**
**Prediction:**	neutral	neutral	neutral	neutral	neutral	neutral
**Score:**	-1.0000	8.8810	0.6325	0.5020	0.0000	0.3200
**Exp. accuracy:**	0.96	0.92	0.97	0.70	0.93	0.53

External links

[dbSNP] [GenBank] [RegulomeDB] [UCSC] [Ensemble]

**Variant:**	21 : 32699933, $A \rightarrow$G				
**Position:**	chr21:32699933	**Ref. allele:**	A	**Alt. allele:**	G
**Region:**	exonic	**Exonic. func.:**	synonymous	**Gene ID:**	SYNJ1
**RS number:**	-	**Clin. sign.:**	-	-	

Results

**Tool:**	**PredictSNP2**	**CADD**	**DANN**	**FATHMM**	**FunSeq2**	**GWAVA**
**Prediction:**	neutral	neutral	neutral	neutral	neutral	neutral
**Score:**	-1.0000	8.1060	0.6190	0.5789	0.0000	0.3100
**Exp. accuracy:**	0.96	0.91	0.96	0.70	0.93	0.53

External links

[GenBank] [UCSC] [Ensemble]

**Variant:**	21 : 32699966, $T \rightarrow$C				
**Position:**	chr21:32699966	**Ref. allele:**	T	**Alt. allele:**	C
**Region:**	exonic	**Exonic. func.:**	synonymous	**Gene ID:**	SYNJ1
**RS number:**	rs144142216	**Clin. sign.:**	-	-	

Results

**Tool:**	**PredictSNP2**	**CADD**	**DANN**	**FATHMM**	**FunSeq2**	**GWAVA**
**Prediction:**	neutral	neutral	neutral	neutral	neutral	neutral
**Score:**	-1.0000	7.5850	0.7021	0.1915	0.0000	0.3400
**Exp. accuracy:**	0.96	0.91	0.94	0.80	0.93	0.56

External links

[dbSNP] [GenBank] [HaploReg] [RegulomeDB] [UCSC] [Ensemble]

Variant: 21 : 32699981, $A \rightarrow$G					
**Position:**	chr21:32699981	**Ref. allele:**	A	**Alt. allele:**	G
**Region:**	exonic	**Exonic. func.:**	synonymous	**Gene ID:**	SYNJ1
**RS number:**	-	**Clin. sign.:**	-	-	

Results

Tool:	PredictSNP2	CADD	DANN	FATHMM	FunSeq2	GWAVA
**Prediction:**	neutral	neutral	neutral	neutral	neutral	neutral
**Score:**	-1.0000	9.8800	0.6187	0.6953	0.0000	0.3800
**Exp. accuracy:**	0.96	0.91	0.96	0.69	0.93	0.55

External links

[GenBank] [UCSC] [Ensemble]

Variant: 21 : 32699993, $G \rightarrow$A					
**Position:**	chr21:32699993	**Ref. allele:**	G	**Alt. allele:**	A
**Region:**	exonic	**Exonic. func.:**	synonymous	**Gene ID:**	SYNJ1
**RS number:**	-	**Clin. sign.:**	-	-	

Results

Tool:	PredictSNP2	CADD	DANN	FATHMM	FunSeq2	GWAVA
**Prediction:**	neutral	neutral	neutral	neutral	neutral	neutral
**Score:**	-1.0000	9.9700	0.7216	0.0238	0.0000	0.2900
**Exp. accuracy:**	0.96	0.91	0.93	0.96	0.93	0.56

External links

[GenBank] [UCSC] [Ensemble]

Variant: 21 : 32700011, $C \rightarrow$T					
**Position:**	chr21:32700011	**Ref. allele:**	C	**Alt. allele:**	T
**Region:**	exonic	**Exonic. func.:**	synonymous	**Gene ID:**	SYNJ1
**RS number:**	-	**Clin. sign.:**	-	-	

Results

Tool:	PredictSNP2	CADD	DANN	FATHMM	FunSeq2	GWAVA
**Prediction:**	neutral	neutral	neutral	neutral	neutral	neutral
**Score:**	-1.0000	13.2600	0.5508	0.5797	0.0000	0.3400
**Exp. accuracy:**	0.96	0.74	0.97	0.70	0.93	0.56

External links

[GenBank] [UCSC] [Ensemble]

Variant: 21 : 32700017, $G \to$C					
**Position:**	chr21:32700017	**Ref. allele:**	G	**Alt. allele:**	C
**Region:**	exonic	**Exonic. func.:**	synonymous	**Gene ID:**	SYNJ1
**RS number:**	rs192548936	**Clin. sign.:**	-	-	

Results

**Tool:**	**PredictSNP2**	**CADD**	**DANN**	**FATHMM**	**FunSeq2**	**GWAVA**
**Prediction:**	neutral	neutral	neutral	deleterious	neutral	neutral
**Score:**	-0.5537	8.9800	0.7463	0.8768	0.0000	0.4300
**Exp. accuracy:**	0.93	0.92	0.91	0.64	0.93	0.54

External links

[dbSNP] [GenBank] [HaploReg] [RegulomeDB] [UCSC] [Ensemble]

Variant: 21 : 32700029, $G \to$A					
**Position:**	chr21:32700029	**Ref. allele:**	G	**Alt. allele:**	A
**Region:**	exonic	**Exonic. func.:**	synonymous	**Gene ID:**	SYNJ1
**RS number:**	-	**Clin. sign.:**	-	-	

Results

**Tool:**	**PredictSNP2**	**CADD**	**DANN**	**FATHMM**	**FunSeq2**	**GWAVA**
**Prediction:**	neutral	neutral	neutral	deleterious	neutral	neutral
**Score:**	-0.5757	10.0800	0.8005	0.8170	0.0000	0.3800
**Exp. accuracy:**	0.93	0.91	0.90	0.57	0.93	0.55

External links

[GenBank] [UCSC] [Ensemble]

Variant: 21 : 32700074, $G \to$A					
**Position:**	chr21:32700074	**Ref. allele:**	G	**Alt. allele:**	A
**Region:**	exonic	**Exonic. func.:**	synonymous	**Gene ID:**	SYNJ1
**RS number:**	-	**Clin. sign.:**	-	-	

Results

**Tool:**	**PredictSNP2**	**CADD**	**DANN**	**FATHMM**	**FunSeq2**	**GWAVA**
**Prediction:**	neutral	neutral	neutral	deleterious	neutral	neutral
**Score:**	-0.5867	9.9660	0.6728	0.8105	0.0000	0.4400
**Exp. accuracy:**	0.93	0.91	0.94	0.57	0.93	0.54

External links

[GenBank] [UCSC] [Ensemble]

**Variant:** 21 : 32700079, $G \rightarrow$A					
**Position:**	chr21:32700079	**Ref. allele:**	G	**Alt. allele:**	A
**Region:**	exonic	**Exonic. func.:**	synonymous	**Gene ID:**	SYNJ1
**RS number:**	-	**Clin. sign.:**	-	-	

Results

**Tool:**	**PredictSNP2**	**CADD**	**DANN**	**FATHMM**	**FunSeq2**	**GWAVA**
**Prediction:**	neutral	neutral	neutral	deleterious	neutral	neutral
**Score:**	-0.5196	10.2000	0.7328	0.8968	0.0000	0.4500
**Exp. accuracy:**	0.93	0.88	0.91	0.71	0.93	0.54

External links

[GenBank] [UCSC] [Ensemble]

**Variant:** 21 : 32700098, $A \rightarrow$G					
**Position:**	chr21:32700098	**Ref. allele:**	A	**Alt. allele:**	G
**Region:**	exonic	**Exonic. func.:**	synonymous	**Gene ID:**	SYNJ1
**RS number:**	-	**Clin. sign.:**	-	-	

Results

**Tool:**	**PredictSNP2**	**CADD**	**DANN**	**FATHMM**	**FunSeq2**	**GWAVA**
**Prediction:**	neutral	neutral	neutral	neutral	neutral	neutral
**Score:**	-1.0000	0.0720	0.6492	0.0301	0.0000	0.4700
**Exp. accuracy:**	0.96	0.95	0.96	0.96	0.93	0.54

External links

[GenBank] [UCSC] [Ensemble]

**Variant:** 21 : 32701986, $G \rightarrow$C					
**Position:**	chr21:32701986	**Ref. allele:**	G	**Alt. allele:**	C
**Region:**	exonic	**Exonic. func.:**	synonymous	**Gene ID:**	SYNJ1
**RS number:**	-	**Clin. sign.:**	-	-	

Results

**Tool:**	**PredictSNP2**	**CADD**	**DANN**	**FATHMM**	**FunSeq2**	**GWAVA**
**Prediction:**	neutral	neutral	neutral	neutral	neutral	neutral
**Score:**	-1.0000	9.1410	0.7876	0.2648	0.0000	0.4600
**Exp. accuracy:**	0.96	0.92	0.89	0.73	0.93	0.54

External links

[GenBank] [UCSC] [Ensemble]

**Variant:** 21 : 32701995, $T \rightarrow$C					
**Position:**	chr21:32701995	**Ref. allele:**	T	**Alt. allele:**	C
**Region:**	exonic	**Exonic. func.:**	synonymous	**Gene ID:**	SYNJ1
**RS number:**	-	**Clin. sign.:**	-	-	

Results

**Tool:**	**PredictSNP2**	**CADD**	**DANN**	**FATHMM**	**FunSeq2**	**GWAVA**
**Prediction:**	neutral	neutral	neutral	neutral	neutral	neutral
**Score:**	-1.0000	7.5070	0.7195	0.3393	0.0000	0.4200
**Exp. accuracy:**	0.96	0.91	0.93	0.73	0.93	0.54

External links

[GenBank] [UCSC] [Ensemble]

**Variant:** 21 : 32701995, $T \rightarrow$G					
**Position:**	chr21:32701995	**Ref. allele:**	T	**Alt. allele:**	G
**Region:**	exonic	**Exonic. func.:**	synonymous	**Gene ID:**	SYNJ1
**RS number:**	-	**Clin. sign.:**	-	-	

Results

**Tool:**	**PredictSNP2**	**CADD**	**DANN**	**FATHMM**	**FunSeq2**	**GWAVA**
**Prediction:**	neutral	neutral	neutral	neutral	neutral	neutral
**Score:**	-1.0000	9.8140	0.7124	0.2516	0.0000	0.4200
**Exp. accuracy:**	0.96	0.91	0.93	0.73	0.93	0.54

External links

[GenBank] [UCSC] [Ensemble]

**Variant:** 21 : 32702007, $T \rightarrow$C					
**Position:**	chr21:32702007	**Ref. allele:**	T	**Alt. allele:**	C
**Region:**	exonic	**Exonic. func.:**	synonymous	**Gene ID:**	SYNJ1
**RS number:**	rs141138675	**Clin. sign.:**	-	-	

Results

**Tool:**	**PredictSNP2**	**CADD**	**DANN**	**FATHMM**	**FunSeq2**	**GWAVA**
**Prediction:**	neutral	neutral	neutral	deleterious	neutral	neutral
**Score:**	-0.5722	8.7970	0.6698	0.8692	0.0000	0.4500
**Exp. accuracy:**	0.93	0.92	0.94	0.60	0.93	0.54

External links

[dbSNP] [GenBank] [HaploReg] [RegulomeDB] [UCSC] [Ensemble]

Variant:	21 : 32702010, $G{\rightarrow}$C				
**Position:**	chr21:32702010	**Ref. allele:**	G	**Alt. allele:**	C
**Region:**	exonic	**Exonic. func.:**	synonymous	**Gene ID:**	SYNJ1
**RS number:**	-	**Clin. sign.:**	-	-	

Results

Tool:	PredictSNP2	CADD	DANN	FATHMM	FunSeq2	GWAVA
**Prediction:**	neutral	neutral	neutral	deleterious	neutral	neutral
**Score:**	-0.4941	8.7750	0.7693	0.9146	0.0000	0.4700
**Exp. accuracy:**	0.93	0.92	0.90	0.77	0.93	0.54

External links

[GenBank] [UCSC] [Ensemble]

Variant:	21 : 32702016, $T{\rightarrow}$C				
**Position:**	chr21:32702016	**Ref. allele:**	T	**Alt. allele:**	C
**Region:**	exonic	**Exonic. func.:**	synonymous	**Gene ID:**	SYNJ1
**RS number:**	rs144728528	**Clin. sign.:**	-	-	

Results

Tool:	PredictSNP2	CADD	DANN	FATHMM	FunSeq2	GWAVA
**Prediction:**	neutral	neutral	neutral	neutral	neutral	neutral
**Score:**	-1.0000	5.3320	0.6940	0.1508	0.0000	0.4400
**Exp. accuracy:**	0.96	0.92	0.94	0.83	0.93	0.54

External links

[dbSNP] [GenBank] [HaploReg] [RegulomeDB] [UCSC] [Ensemble]

Variant:	21 : 32726776, $C{\rightarrow}$A				
**Position:**	chr21:32726776	**Ref. allele:**	C	**Alt. allele:**	A
**Region:**	exonic	**Exonic. func.:**	synonymous	**Gene ID:**	SYNJ1
**RS number:**	-	**Clin. sign.:**	-	-	

Results

Tool:	PredictSNP2	CADD	DANN	FATHMM	FunSeq2	GWAVA
**Prediction:**	neutral	deleterious	neutral	neutral	neutral	neutral
**Score:**	-0.4885	21.6000	0.8257	0.4212	0.0000	0.4700
**Exp. accuracy:**	0.93	0.82	0.90	0.72	0.93	0.54

External links

[GenBank] [UCSC] [Ensemble]

**Variant:** 21 : 32726797, $G \rightarrow$A					
**Position:**	chr21:32726797	**Ref. allele:**	G	**Alt. allele:**	A
**Region:**	exonic	**Exonic. func.:**	synonymous	**Gene ID:**	SYNJ1
**RS number:**	-	**Clin. sign.:**	-	-	

Results

**Tool:**	**PredictSNP2**	**CADD**	**DANN**	**FATHMM**	**FunSeq2**	**GWAVA**
**Prediction:**	neutral	deleterious	neutral	neutral	neutral	deleterious
**Score:**	-0.4604	22.2000	0.8817	0.4664	0.0000	0.5300
**Exp. accuracy:**	0.93	0.87	0.90	0.72	0.93	0.64

External links

[GenBank] [UCSC] [Ensemble]

**Variant:** 21 : 32726803, $G \rightarrow$C					
**Position:**	chr21:32726803	**Ref. allele:**	G	**Alt. allele:**	C
**Region:**	exonic	**Exonic. func.:**	synonymous	**Gene ID:**	SYNJ1
**RS number:**	rs201322530	**Clin. sign.:**	-	-	

Results

**Tool:**	**PredictSNP2**	**CADD**	**DANN**	**FATHMM**	**FunSeq2**	**GWAVA**
**Prediction:**	neutral	deleterious	neutral	neutral	neutral	deleterious
**Score:**	-0.4899	21.5000	0.8541	0.6480	0.0000	0.5400
**Exp. accuracy:**	0.93	0.79	0.90	0.69	0.93	0.62

External links

[dbSNP] [GenBank] [HaploReg] [RegulomeDB] [UCSC] [Ensemble]

**Variant:** 21 : 32726833, $T \rightarrow$G					
**Position:**	chr21:32726833	**Ref. allele:**	T	**Alt. allele:**	G
**Region:**	exonic	**Exonic. func.:**	synonymous	**Gene ID:**	SYNJ1
**RS number:**	-	**Clin. sign.:**	-	-	

Results

**Tool:**	**PredictSNP2**	**CADD**	**DANN**	**FATHMM**	**FunSeq2**	**GWAVA**
**Prediction:**	neutral	deleterious	neutral	neutral	neutral	deleterious
**Score:**	-0.5276	21.5000	0.7677	0.1266	0.0000	0.5000
**Exp. accuracy:**	0.93	0.79	0.90	0.85	0.93	0.51

External links

[GenBank] [UCSC] [Ensemble]

**Variant:** 21 : 32726890, $C \to$T					
**Position:**	chr21:32726890	**Ref. allele:**	C	**Alt. allele:**	T
**Region:**	exonic	**Exonic. func.:**	synonymous	**Gene ID:**	SYNJ1
**RS number:**	rs61750221	**Clin. sign.:**	-	-	

Results

**Tool:**	**PredictSNP2**	**CADD**	**DANN**	**FATHMM**	**FunSeq2**	**GWAVA**
**Prediction:**	neutral	deleterious	neutral	neutral	neutral	deleterious
**Score:**	-0.4596	22.3000	0.8804	0.3575	0.0000	0.5600
**Exp. accuracy:**	0.93	0.87	0.90	0.73	0.93	0.69

External links

[dbSNP] [GenBank] [HaploReg] [RegulomeDB] [UCSC] [Ensemble]

**Variant:** 21 : 32727954, $C \to$G					
**Position:**	chr21:32727954	**Ref. allele:**	C	**Alt. allele:**	G
**Region:**	exonic	**Exonic. func.:**	synonymous	**Gene ID:**	SYNJ1
**RS number:**	-	**Clin. sign.:**	-	-	

Results

**Tool:**	**PredictSNP2**	**CADD**	**DANN**	**FATHMM**	**FunSeq2**	**GWAVA**
**Prediction:**	neutral	deleterious	neutral	neutral	neutral	deleterious
**Score:**	-0.4597	22.2000	0.8636	0.6965	0.0000	0.6000
**Exp. accuracy:**	0.93	0.87	0.90	0.69	0.93	0.64

External links

[GenBank] [UCSC] [Ensemble]

**Variant:** 21 : 32727972, $T \to$C					
**Position:**	chr21:32727972	**Ref. allele:**	T	**Alt. allele:**	C
**Region:**	exonic	**Exonic. func.:**	synonymous	**Gene ID:**	SYNJ1
**RS number:**	-	**Clin. sign.:**	-	-	

Results

**Tool:**	**PredictSNP2**	**CADD**	**DANN**	**FATHMM**	**FunSeq2**	**GWAVA**
**Prediction:**	neutral	deleterious	neutral	neutral	neutral	deleterious
**Score:**	-0.6080	19.5900	0.6997	0.0173	0.0000	0.6000
**Exp. accuracy:**	0.95	0.69	0.94	0.96	0.93	0.64

External links

[GenBank] [UCSC] [Ensemble]

**Variant:** 21 : 32727987, $G \to$A					
**Position:**	chr21:32727987	**Ref. allele:**	G	**Alt. allele:**	A
**Region:**	exonic	**Exonic. func.:**	synonymous	**Gene ID:**	SYNJ1
**RS number:**	-	**Clin. sign.:**	-	-	

Results

**Tool:**	**PredictSNP2**	**CADD**	**DANN**	**FATHMM**	**FunSeq2**	**GWAVA**
**Prediction:**	deleterious	deleterious	deleterious	neutral	neutral	deleterious
**Score:**	0.0282	22.4000	0.9348	0.7578	0.0000	0.5100
**Exp. accuracy:**	0.93	0.87	0.87	0.69	0.93	0.55

External links

[GenBank] [UCSC] [Ensemble]

**Variant:** 21 : 32727996, $G \to$A					
**Position:**	chr21:32727996	**Ref. allele:**	G	**Alt. allele:**	A
**Region:**	exonic	**Exonic. func.:**	synonymous	**Gene ID:**	SYNJ1
**RS number:**	-	**Clin. sign.:**	-	-	

Results

**Tool:**	**PredictSNP2**	**CADD**	**DANN**	**FATHMM**	**FunSeq2**	**GWAVA**
**Prediction:**	deleterious	deleterious	deleterious	neutral	neutral	neutral
**Score:**	0.0199	21.4000	0.9425	0.3648	0.0000	0.4100
**Exp. accuracy:**	0.93	0.79	0.87	0.72	0.93	0.56

External links

[GenBank] [UCSC] [Ensemble]
