# Supplementary material for: Computational analysis of functional SNPs in Alzheimer’s disease-associated endocytosis genes
Source: PeerJ. 2019 Sep 30;7:e7667. doi: 10.7717/peerj.7667 (PMC6776068; doi:10.7717/peerj.7667)
Supplement: Table S7 — The result file was generated by PredictSNP2. [file peerj-07-7667-s011.pdf]

PredictSNP overview

Date: 2017/03/06
Genome version: GRCh38/hg38
PredictSNP version: 2.1

If you use PredictSNP and obtain scientific results that you publish, we would ask you to acknowledge the usage of PredictSNP by referencing the article below.

Bendl, J., Musil, M., Stourac, J., Zendulka, J., Damborsky, J., Brezovsky, J., 2016: PredictSNP2: A unified platform for accurately evaluating SNP effects by exploiting the different characteristics of variants in distinct genomic regions. *PLOS Computational Biology* 12: e1004962

	PredictSNP2		CADD		DANN		FATHMM		FunSeq2		GWAVA	
	Neu	Del	Neu	Del	Neu	Del	Neu	Del	Neu	Del	Neu	Del
Regulatory	0	0	0	0	0	0	0	0	0	0	0	0
Splicing	0	0	0	0	0	0	0	0	0	0	0	0
Missense	0	0	0	0	0	0	61	0	60	1	61	0
Synonymous	55	6	61	0	1	56	0	0	0	0	0	0
Nonsense	0	0	0	0	0	0	0	0	0	0	0	0
Others	0	0	0	0	0	0	0	0	0	0	0	0

**Variant:** $X : 19536447, G \to$A					
**Position:**	chrX:19536447	**Ref. allele:**	G	**Alt. allele:**	A
**Region:**	exonic	**Exonic. func.:**	synonymous	**Gene ID:**	SH3KBP1
**RS number:**	-	**Clin. sign.:**	-	-	

Results

**Tool:**	**PredictSNP2**	**CADD**	**DANN**	**FATHMM**	**FunSeq2**	**GWAVA**
**Prediction:**	neutral	neutral	neutral	neutral	neutral	neutral
**Score:**	-1.0000	7.1800	0.7355	0.2855	0.0000	0.3700
**Exp. accuracy:**	0.96	0.91	0.91	0.73	0.93	0.56

External links

[GenBank] [UCSC] [Ensemble]

**Variant:** $X : 19537720, C \to$T					
**Position:**	chrX:19537720	**Ref. allele:**	C	**Alt. allele:**	T
**Region:**	exonic	**Exonic. func.:**	synonymous	**Gene ID:**	SH3KBP1
**RS number:**	-	**Clin. sign.:**	-	-	

Results

**Tool:**	**PredictSNP2**	**CADD**	**DANN**	**FATHMM**	**FunSeq2**	**GWAVA**
**Prediction:**	neutral	neutral	neutral	neutral	neutral	deleterious
**Score:**	-1.0000	11.9700	0.8057	0.6143	0.0000	0.6600
**Exp. accuracy:**	0.96	0.83	0.90	0.69	0.93	0.70

External links

[GenBank] [UCSC] [Ensemble]

**Variant:** $X : 19537729, C \to$T					
**Position:**	chrX:19537729	**Ref. allele:**	C	**Alt. allele:**	T
**Region:**	exonic	**Exonic. func.:**	synonymous	**Gene ID:**	SH3KBP1
**RS number:**	-	**Clin. sign.:**	-	-	

Results

**Tool:**	**PredictSNP2**	**CADD**	**DANN**	**FATHMM**	**FunSeq2**	**GWAVA**
**Prediction:**	neutral	neutral	neutral	neutral	neutral	deleterious
**Score:**	-1.0000	14.3600	0.7041	0.3061	0.0000	0.5800
**Exp. accuracy:**	0.96	0.73	0.94	0.73	0.93	0.65

External links

[GenBank] [UCSC] [Ensemble]

**Variant:** $X:19537752, A \to$G					
**Position:**	chrX:19537752	**Ref. allele:**	A	**Alt. allele:**	G
**Region:**	exonic	**Exonic. func.:**	synonymous	**Gene ID:**	SH3KBP1
**RS number:**	-	**Clin. sign.:**	-	-	

Results

**Tool:**	**PredictSNP2**	**CADD**	**DANN**	**FATHMM**	**FunSeq2**	**GWAVA**
**Prediction:**	neutral	neutral	neutral	neutral	neutral	deleterious
**Score:**	-1.0000	9.1330	0.6802	0.3487	0.0000	0.6200
**Exp. accuracy:**	0.96	0.92	0.94	0.73	0.93	0.66

External links

[GenBank] [UCSC] [Ensemble]

**Variant:** $X:19541936, C \to$T					
**Position:**	chrX:19541936	**Ref. allele:**	C	**Alt. allele:**	T
**Region:**	exonic	**Exonic. func.:**	synonymous	**Gene ID:**	SH3KBP1
**RS number:**	-	**Clin. sign.:**	-	-	

Results

**Tool:**	**PredictSNP2**	**CADD**	**DANN**	**FATHMM**	**FunSeq2**	**GWAVA**
**Prediction:**	neutral	neutral	neutral	neutral	neutral	deleterious
**Score:**	-1.0000	13.5700	0.6133	0.5550	0.0000	0.6000
**Exp. accuracy:**	0.96	0.73	0.96	0.70	0.93	0.64

External links

[GenBank] [UCSC] [Ensemble]

**Variant:** $X:19541975, T \to$G					
**Position:**	chrX:19541975	**Ref. allele:**	T	**Alt. allele:**	G
**Region:**	exonic	**Exonic. func.:**	synonymous	**Gene ID:**	SH3KBP1
**RS number:**	-	**Clin. sign.:**	-	-	

Results

**Tool:**	**PredictSNP2**	**CADD**	**DANN**	**FATHMM**	**FunSeq2**	**GWAVA**
**Prediction:**	neutral	neutral	neutral	neutral	neutral	?
**Score:**	-1.0000	2.6240	0.5131	0.0507	0.0000	0.4800
**Exp. accuracy:**	0.96	0.95	0.97	0.94	0.93	0.43

External links

[GenBank] [UCSC] [Ensemble]

Variant: $X: 19541987, C \rightarrow$T					
**Position:**	chrX:19541987	**Ref. allele:**	C	**Alt. allele:**	T
**Region:**	exonic	**Exonic. func.:**	synonymous	**Gene ID:**	SH3KBP1
**RS number:**	rs61740276	**Clin. sign.:**	-	-	

Results

**Tool:**	**PredictSNP2**	**CADD**	**DANN**	**FATHMM**	**FunSeq2**	**GWAVA**
**Prediction:**	neutral	neutral	neutral	neutral	neutral	deleterious
**Score:**	-1.0000	13.6100	0.6618	0.5557	0.0000	0.5900
**Exp. accuracy:**	0.96	0.73	0.95	0.70	0.93	0.62

External links

[dbSNP] [GenBank] [HaploReg] [RegulomeDB] [UCSC] [Ensemble]

Variant: $X: 19541993, G \rightarrow$A					
**Position:**	chrX:19541993	**Ref. allele:**	G	**Alt. allele:**	A
**Region:**	exonic	**Exonic. func.:**	synonymous	**Gene ID:**	SH3KBP1
**RS number:**	rs11552353	**Clin. sign.:**	-	-	

Results

**Tool:**	**PredictSNP2**	**CADD**	**DANN**	**FATHMM**	**FunSeq2**	**GWAVA**
**Prediction:**	neutral	neutral	neutral	neutral	neutral	deleterious
**Score:**	-1.0000	13.7100	0.7168	0.2459	0.0000	0.5000
**Exp. accuracy:**	0.96	0.73	0.93	0.73	0.93	0.51

External links

[dbSNP] [GenBank] [HaploReg] [RegulomeDB] [UCSC] [Ensemble]

Variant: $X: 19541993, G \rightarrow$C					
**Position:**	chrX:19541993	**Ref. allele:**	G	**Alt. allele:**	C
**Region:**	exonic	**Exonic. func.:**	synonymous	**Gene ID:**	SH3KBP1
**RS number:**	-	**Clin. sign.:**	-	-	

Results

**Tool:**	**PredictSNP2**	**CADD**	**DANN**	**FATHMM**	**FunSeq2**	**GWAVA**
**Prediction:**	neutral	neutral	neutral	neutral	neutral	deleterious
**Score:**	-1.0000	10.7100	0.5989	0.3103	0.0000	0.5000
**Exp. accuracy:**	0.96	0.88	0.96	0.73	0.93	0.51

External links

[GenBank] [UCSC] [Ensemble]

Variant: $X : 19542005, G \rightarrow$A					
**Position:**	chrX:19542005	**Ref. allele:**	G	**Alt. allele:**	A
**Region:**	exonic	**Exonic. func.:**	synonymous	**Gene ID:**	SH3KBP1
**RS number:**	-	**Clin. sign.:**	-	-	

Results

**Tool:**	**PredictSNP2**	**CADD**	**DANN**	**FATHMM**	**FunSeq2**	**GWAVA**
**Prediction:**	neutral	neutral	neutral	neutral	neutral	deleterious
**Score:**	-1.0000	10.6400	0.7234	0.5747	0.0000	0.5400
**Exp. accuracy:**	0.96	0.88	0.93	0.70	0.93	0.62

External links

[GenBank] [UCSC] [Ensemble]

Variant: $X : 19542011, C \rightarrow$T					
**Position:**	chrX:19542011	**Ref. allele:**	C	**Alt. allele:**	T
**Region:**	exonic	**Exonic. func.:**	synonymous	**Gene ID:**	SH3KBP1
**RS number:**	rs140545590	**Clin. sign.:**	-	-	

Results

**Tool:**	**PredictSNP2**	**CADD**	**DANN**	**FATHMM**	**FunSeq2**	**GWAVA**
**Prediction:**	neutral	neutral	neutral	neutral	neutral	deleterious
**Score:**	-1.0000	15.6200	0.7293	0.0958	0.0000	0.5700
**Exp. accuracy:**	0.96	0.73	0.91	0.87	0.93	0.68

External links

[dbSNP] [GenBank] [HaploReg] [RegulomeDB] [UCSC] [Ensemble]

Variant: $X : 19542038, C \rightarrow$T					
**Position:**	chrX:19542038	**Ref. allele:**	C	**Alt. allele:**	T
**Region:**	exonic	**Exonic. func.:**	synonymous	**Gene ID:**	SH3KBP1
**RS number:**	rs150468291	**Clin. sign.:**	-	-	

Results

**Tool:**	**PredictSNP2**	**CADD**	**DANN**	**FATHMM**	**FunSeq2**	**GWAVA**
**Prediction:**	neutral	neutral	neutral	neutral	neutral	deleterious
**Score:**	-1.0000	11.8800	0.5898	0.0442	0.0000	0.5200
**Exp. accuracy:**	0.96	0.83	0.97	0.96	0.93	0.58

External links

[dbSNP] [GenBank] [HaploReg] [RegulomeDB] [UCSC] [Ensemble]

**Variant:** $X : 19542044, G \rightarrow$A					
**Position:**	chrX:19542044	**Ref. allele:**	G	**Alt. allele:**	A
**Region:**	exonic	**Exonic. func.:**	synonymous	**Gene ID:**	SH3KBP1
**RS number:**	rs199710100	**Clin. sign.:**	-	-	

Results

**Tool:**	**PredictSNP2**	**CADD**	**DANN**	**FATHMM**	**FunSeq2**	**GWAVA**
**Prediction:**	neutral	neutral	neutral	neutral	neutral	deleterious
**Score:**	-1.0000	5.5370	0.5913	0.3367	0.0000	0.5100
**Exp. accuracy:**	0.96	0.92	0.96	0.73	0.93	0.55

External links

[dbSNP] [GenBank] [HaploReg] [RegulomeDB] [UCSC] [Ensemble]

**Variant:** $X : 19542053, C \rightarrow$T					
**Position:**	chrX:19542053	**Ref. allele:**	C	**Alt. allele:**	T
**Region:**	exonic	**Exonic. func.:**	synonymous	**Gene ID:**	SH3KBP1
**RS number:**	rs200949699	**Clin. sign.:**	-	-	

Results

**Tool:**	**PredictSNP2**	**CADD**	**DANN**	**FATHMM**	**FunSeq2**	**GWAVA**
**Prediction:**	neutral	neutral	neutral	neutral	neutral	?
**Score:**	-1.0000	10.8700	0.7399	0.0192	0.0000	0.4800
**Exp. accuracy:**	0.96	0.88	0.91	0.96	0.93	0.43

External links

[dbSNP] [GenBank] [HaploReg] [RegulomeDB] [UCSC] [Ensemble]

**Variant:** $X : 19542071, T \rightarrow$C					
**Position:**	chrX:19542071	**Ref. allele:**	T	**Alt. allele:**	C
**Region:**	exonic	**Exonic. func.:**	synonymous	**Gene ID:**	SH3KBP1
**RS number:**	-	**Clin. sign.:**	-	-	

Results

**Tool:**	**PredictSNP2**	**CADD**	**DANN**	**FATHMM**	**FunSeq2**	**GWAVA**
**Prediction:**	neutral	neutral	neutral	neutral	neutral	deleterious
**Score:**	-1.0000	4.1700	0.6976	0.6541	0.0000	0.6600
**Exp. accuracy:**	0.96	0.94	0.94	0.69	0.93	0.70

External links

[GenBank] [UCSC] [Ensemble]

**Variant:** $X:19542097, G{\to}$A					
**Position:**	chrX:19542097	**Ref. allele:**	G	**Alt. allele:**	A
**Region:**	exonic	**Exonic. func.:**	synonymous	**Gene ID:**	SH3KBP1
**RS number:**	-	**Clin. sign.:**	-	-	

Results

**Tool:**	**PredictSNP2**	**CADD**	**DANN**	**FATHMM**	**FunSeq2**	**GWAVA**
**Prediction:**	neutral	neutral	neutral	neutral	neutral	deleterious
**Score:**	-1.0000	0.6800	0.6000	0.3557	0.0000	0.5500
**Exp. accuracy:**	0.96	0.95	0.96	0.73	0.93	0.66

External links

[GenBank] [UCSC] [Ensemble]

**Variant:** $X:19542098, G{\to}$A					
**Position:**	chrX:19542098	**Ref. allele:**	G	**Alt. allele:**	A
**Region:**	exonic	**Exonic. func.:**	synonymous	**Gene ID:**	SH3KBP1
**RS number:**	-	**Clin. sign.:**	-	-	

Results

**Tool:**	**PredictSNP2**	**CADD**	**DANN**	**FATHMM**	**FunSeq2**	**GWAVA**
**Prediction:**	neutral	neutral	neutral	neutral	neutral	deleterious
**Score:**	-1.0000	0.1980	0.5993	0.0417	0.0000	0.5000
**Exp. accuracy:**	0.96	0.95	0.96	0.96	0.93	0.51

External links

[GenBank] [UCSC] [Ensemble]

**Variant:** $X:19542098, G{\to}$C					
**Position:**	chrX:19542098	**Ref. allele:**	G	**Alt. allele:**	C
**Region:**	exonic	**Exonic. func.:**	synonymous	**Gene ID:**	SH3KBP1
**RS number:**	-	**Clin. sign.:**	-	-	

Results

**Tool:**	**PredictSNP2**	**CADD**	**DANN**	**FATHMM**	**FunSeq2**	**GWAVA**
**Prediction:**	neutral	neutral	neutral	neutral	neutral	deleterious
**Score:**	-1.0000	0.0110	0.3655	0.0732	0.0000	0.5000
**Exp. accuracy:**	0.96	0.95	0.97	0.90	0.93	0.51

External links

[GenBank] [UCSC] [Ensemble]

**Variant:** $X : 19542098, G{\to}$T					
**Position:**	chrX:19542098	**Ref. allele:**	G	**Alt. allele:**	T
**Region:**	exonic	**Exonic. func.:**	synonymous	**Gene ID:**	SH3KBP1
**RS number:**	rs376960635	**Clin. sign.:**	-	-	

Results

**Tool:**	**PredictSNP2**	**CADD**	**DANN**	**FATHMM**	**FunSeq2**	**GWAVA**
**Prediction:**	neutral	neutral	neutral	neutral	neutral	deleterious
**Score:**	-1.0000	0.0090	0.4426	0.0587	0.0000	0.5000
**Exp. accuracy:**	0.96	0.95	0.97	0.92	0.93	0.51

External links

[dbSNP] [GenBank] [RegulomeDB] [UCSC] [Ensemble]

**Variant:** $X : 19542104, G{\to}$C					
**Position:**	chrX:19542104	**Ref. allele:**	G	**Alt. allele:**	C
**Region:**	exonic	**Exonic. func.:**	synonymous	**Gene ID:**	SH3KBP1
**RS number:**	-	**Clin. sign.:**	-	-	

Results

**Tool:**	**PredictSNP2**	**CADD**	**DANN**	**FATHMM**	**FunSeq2**	**GWAVA**
**Prediction:**	neutral	neutral	neutral	neutral	neutral	deleterious
**Score:**	-1.0000	0.0100	0.3369	0.3712	0.0000	0.5000
**Exp. accuracy:**	0.96	0.95	0.97	0.72	0.93	0.51

External links

[GenBank] [UCSC] [Ensemble]

**Variant:** $X : 19542107, C{\to}$T					
**Position:**	chrX:19542107	**Ref. allele:**	C	**Alt. allele:**	T
**Region:**	exonic	**Exonic. func.:**	synonymous	**Gene ID:**	SH3KBP1
**RS number:**	rs143070322	**Clin. sign.:**	-	-	

Results

**Tool:**	**PredictSNP2**	**CADD**	**DANN**	**FATHMM**	**FunSeq2**	**GWAVA**
**Prediction:**	neutral	neutral	neutral	neutral	neutral	deleterious
**Score:**	-1.0000	9.9830	0.3822	0.0139	0.0000	0.5200
**Exp. accuracy:**	0.96	0.91	0.97	0.96	0.93	0.58

External links

[dbSNP] [GenBank] [HaploReg] [RegulomeDB] [UCSC] [Ensemble]

**Variant:** $X : 19542110, C \to$ G					
**Position:**	chrX:19542110	**Ref. allele:**	C	**Alt. allele:**	G
**Region:**	exonic	**Exonic. func.:**	synonymous	**Gene ID:**	SH3KBP1
**RS number:**	-	**Clin. sign.:**	-	-	

Results

**Tool:**	**PredictSNP2**	**CADD**	**DANN**	**FATHMM**	**FunSeq2**	**GWAVA**
**Prediction:**	neutral	neutral	neutral	neutral	neutral	deleterious
**Score:**	-1.0000	5.6680	0.3192	0.0487	0.0000	0.5100
**Exp. accuracy:**	0.96	0.92	0.97	0.94	0.93	0.55

External links

[GenBank] [UCSC] [Ensemble]

**Variant:** $X : 19542110, C \to$ T					
**Position:**	chrX:19542110	**Ref. allele:**	C	**Alt. allele:**	T
**Region:**	exonic	**Exonic. func.:**	synonymous	**Gene ID:**	SH3KBP1
**RS number:**	-	**Clin. sign.:**	-	-	

Results

**Tool:**	**PredictSNP2**	**CADD**	**DANN**	**FATHMM**	**FunSeq2**	**GWAVA**
**Prediction:**	neutral	neutral	neutral	neutral	neutral	deleterious
**Score:**	-1.0000	9.0050	0.4109	0.0212	0.0000	0.5100
**Exp. accuracy:**	0.96	0.92	0.97	0.96	0.93	0.55

External links

[GenBank] [UCSC] [Ensemble]

**Variant:** $X : 19542131, C \to$ G					
**Position:**	chrX:19542131	**Ref. allele:**	C	**Alt. allele:**	G
**Region:**	exonic	**Exonic. func.:**	synonymous	**Gene ID:**	SH3KBP1
**RS number:**	-	**Clin. sign.:**	-	-	

Results

**Tool:**	**PredictSNP2**	**CADD**	**DANN**	**FATHMM**	**FunSeq2**	**GWAVA**
**Prediction:**	neutral	neutral	neutral	neutral	neutral	deleterious
**Score:**	-1.0000	5.0970	0.4500	0.0955	0.0000	0.5400
**Exp. accuracy:**	0.96	0.93	0.97	0.87	0.93	0.62

External links

[GenBank] [UCSC] [Ensemble]

**Variant:** $X: 19542137, G{\to}$A					
**Position:**	chrX:19542137	**Ref. allele:**	G	**Alt. allele:**	A
**Region:**	exonic	**Exonic. func.:**	synonymous	**Gene ID:**	SH3KBP1
**RS number:**	-	**Clin. sign.:**	-	-	

Results

**Tool:**	**PredictSNP2**	**CADD**	**DANN**	**FATHMM**	**FunSeq2**	**GWAVA**
**Prediction:**	neutral	neutral	neutral	neutral	neutral	?
**Score:**	-1.0000	1.0280	0.4294	0.0381	0.0000	0.4800
**Exp. accuracy:**	0.96	0.95	0.97	0.96	0.93	0.43

External links

[GenBank] [UCSC] [Ensemble]

**Variant:** $X: 19542146, T{\to}$C					
**Position:**	chrX:19542146	**Ref. allele:**	T	**Alt. allele:**	C
**Region:**	exonic	**Exonic. func.:**	synonymous	**Gene ID:**	SH3KBP1
**RS number:**	-	**Clin. sign.:**	-	-	

Results

**Tool:**	**PredictSNP2**	**CADD**	**DANN**	**FATHMM**	**FunSeq2**	**GWAVA**
**Prediction:**	neutral	neutral	neutral	neutral	neutral	deleterious
**Score:**	-1.0000	1.1540	0.4126	0.0915	0.0000	0.5200
**Exp. accuracy:**	0.96	0.95	0.97	0.87	0.93	0.58

External links

[GenBank] [UCSC] [Ensemble]

**Variant:** $X: 19542158, C{\to}$T					
**Position:**	chrX:19542158	**Ref. allele:**	C	**Alt. allele:**	T
**Region:**	exonic	**Exonic. func.:**	synonymous	**Gene ID:**	SH3KBP1
**RS number:**	rs373830746	**Clin. sign.:**	-	-	

Results

**Tool:**	**PredictSNP2**	**CADD**	**DANN**	**FATHMM**	**FunSeq2**	**GWAVA**
**Prediction:**	neutral	neutral	neutral	neutral	neutral	deleterious
**Score:**	-1.0000	10.8500	0.6118	0.0358	0.0000	0.5000
**Exp. accuracy:**	0.96	0.88	0.96	0.96	0.93	0.51

External links

[dbSNP] [GenBank] [RegulomeDB] [UCSC] [Ensemble]

**Variant:** $X : 19542167, C \to$ T					
**Position:**	chrX:19542167	**Ref. allele:**	C	**Alt. allele:**	T
**Region:**	exonic	**Exonic. func.:**	synonymous	**Gene ID:**	SH3KBP1
**RS number:**	rs199899970	**Clin. sign.:**	-	-	

Results

**Tool:**	**PredictSNP2**	**CADD**	**DANN**	**FATHMM**	**FunSeq2**	**GWAVA**
**Prediction:**	neutral	neutral	neutral	neutral	neutral	deleterious
**Score:**	-1.0000	9.5680	0.5552	0.0288	0.0000	0.5100
**Exp. accuracy:**	0.96	0.91	0.97	0.96	0.93	0.55

External links

[dbSNP] [GenBank] [HaploReg] [RegulomeDB] [UCSC] [Ensemble]

**Variant:** $X : 19542176, T \to$ G					
**Position:**	chrX:19542176	**Ref. allele:**	T	**Alt. allele:**	G
**Region:**	exonic	**Exonic. func.:**	synonymous	**Gene ID:**	SH3KBP1
**RS number:**	-	**Clin. sign.:**	-	-	

Results

**Tool:**	**PredictSNP2**	**CADD**	**DANN**	**FATHMM**	**FunSeq2**	**GWAVA**
**Prediction:**	neutral	neutral	neutral	neutral	neutral	?
**Score:**	-1.0000	6.9050	0.4208	0.0510	0.0000	0.4800
**Exp. accuracy:**	0.96	0.92	0.97	0.94	0.93	0.43

External links

[GenBank] [UCSC] [Ensemble]

**Variant:** $X : 19545946, A \to$ G					
**Position:**	chrX:19545946	**Ref. allele:**	A	**Alt. allele:**	G
**Region:**	exonic	**Exonic. func.:**	synonymous	**Gene ID:**	SH3KBP1
**RS number:**	-	**Clin. sign.:**	-	-	

Results

**Tool:**	**PredictSNP2**	**CADD**	**DANN**	**FATHMM**	**FunSeq2**	**GWAVA**
**Prediction:**	neutral	neutral	neutral	neutral	neutral	deleterious
**Score:**	-1.0000	4.9020	0.5696	0.3319	0.0000	0.5900
**Exp. accuracy:**	0.96	0.93	0.97	0.73	0.93	0.62

External links

[GenBank] [UCSC] [Ensemble]

**Variant:** $X : 19545958, C \to$T					
**Position:**	chrX:19545958	**Ref. allele:**	C	**Alt. allele:**	T
**Region:**	exonic	**Exonic. func.:**	synonymous	**Gene ID:**	SH3KBP1
**RS number:**	-	**Clin. sign.:**	-	-	

Results

**Tool:**	**PredictSNP2**	**CADD**	**DANN**	**FATHMM**	**FunSeq2**	**GWAVA**
**Prediction:**	neutral	neutral	neutral	neutral	neutral	deleterious
**Score:**	-1.0000	14.1600	0.5692	0.0482	0.0000	0.5700
**Exp. accuracy:**	0.96	0.73	0.97	0.94	0.93	0.68

External links

[GenBank] [UCSC] [Ensemble]

**Variant:** $X : 19545961, G \to$A					
**Position:**	chrX:19545961	**Ref. allele:**	G	**Alt. allele:**	A
**Region:**	exonic	**Exonic. func.:**	synonymous	**Gene ID:**	SH3KBP1
**RS number:**	-	**Clin. sign.:**	-	-	

Results

**Tool:**	**PredictSNP2**	**CADD**	**DANN**	**FATHMM**	**FunSeq2**	**GWAVA**
**Prediction:**	neutral	neutral	neutral	neutral	neutral	deleterious
**Score:**	-1.0000	6.2010	0.6533	0.0883	0.0000	0.5800
**Exp. accuracy:**	0.96	0.93	0.96	0.88	0.93	0.65

External links

[GenBank] [UCSC] [Ensemble]

**Variant:** $X : 19545976, C \to$T					
**Position:**	chrX:19545976	**Ref. allele:**	C	**Alt. allele:**	T
**Region:**	exonic	**Exonic. func.:**	synonymous	**Gene ID:**	SH3KBP1
**RS number:**	-	**Clin. sign.:**	-	-	

Results

**Tool:**	**PredictSNP2**	**CADD**	**DANN**	**FATHMM**	**FunSeq2**	**GWAVA**
**Prediction:**	neutral	neutral	neutral	neutral	neutral	deleterious
**Score:**	-1.0000	15.5400	0.7234	0.2793	0.0000	0.5900
**Exp. accuracy:**	0.96	0.73	0.93	0.73	0.93	0.62

External links

[GenBank] [UCSC] [Ensemble]

**Variant:** $X : 19545979, A{\to}$G					
**Position:**	chrX:19545979	**Ref. allele:**	A	**Alt. allele:**	G
**Region:**	exonic	**Exonic. func.:**	synonymous	**Gene ID:**	SH3KBP1
**RS number:**	-	**Clin. sign.:**	-	-	

Results

**Tool:**	**PredictSNP2**	**CADD**	**DANN**	**FATHMM**	**FunSeq2**	**GWAVA**
**Prediction:**	neutral	neutral	neutral	neutral	neutral	deleterious
**Score:**	-1.0000	8.4100	0.6846	0.6995	0.0000	0.7400
**Exp. accuracy:**	0.96	0.92	0.94	0.69	0.93	0.70

External links

[GenBank] [UCSC] [Ensemble]

**Variant:** $X : 19546009, G{\to}$C					
**Position:**	chrX:19546009	**Ref. allele:**	G	**Alt. allele:**	C
**Region:**	exonic	**Exonic. func.:**	synonymous	**Gene ID:**	SH3KBP1
**RS number:**	rs370412822	**Clin. sign.:**	-	-	

Results

**Tool:**	**PredictSNP2**	**CADD**	**DANN**	**FATHMM**	**FunSeq2**	**GWAVA**
**Prediction:**	neutral	neutral	neutral	neutral	neutral	deleterious
**Score:**	-1.0000	11.8300	0.6293	0.6028	0.0000	0.5900
**Exp. accuracy:**	0.96	0.83	0.97	0.69	0.93	0.62

External links

[dbSNP] [GenBank] [RegulomeDB] [UCSC] [Ensemble]

**Variant:** $X : 19546018, G{\to}$A					
**Position:**	chrX:19546018	**Ref. allele:**	G	**Alt. allele:**	A
**Region:**	exonic	**Exonic. func.:**	synonymous	**Gene ID:**	SH3KBP1
**RS number:**	-	**Clin. sign.:**	-	-	

Results

**Tool:**	**PredictSNP2**	**CADD**	**DANN**	**FATHMM**	**FunSeq2**	**GWAVA**
**Prediction:**	neutral	neutral	neutral	neutral	neutral	deleterious
**Score:**	-1.0000	10.9400	0.6479	0.6411	0.0000	0.7300
**Exp. accuracy:**	0.96	0.88	0.96	0.69	0.93	0.70

External links

[GenBank] [UCSC] [Ensemble]

**Variant:** $X:19546021, G{\rightarrow}$A					
**Position:**	chrX:19546021	**Ref. allele:**	G	**Alt. allele:**	A
**Region:**	exonic	**Exonic. func.:**	synonymous	**Gene ID:**	SH3KBP1
**RS number:**	rs148651015	**Clin. sign.:**	-	-	

Results

**Tool:**	**PredictSNP2**	**CADD**	**DANN**	**FATHMM**	**FunSeq2**	**GWAVA**
**Prediction:**	neutral	neutral	neutral	deleterious	neutral	deleterious
**Score:**	-0.5771	9.7600	0.5955	0.8623	0.0000	0.7800
**Exp. accuracy:**	0.93	0.91	0.96	0.57	0.93	0.70

External links

[dbSNP] [GenBank] [HaploReg] [RegulomeDB] [UCSC] [Ensemble]

**Variant:** $X:19546024, G{\rightarrow}$A					
**Position:**	chrX:19546024	**Ref. allele:**	G	**Alt. allele:**	A
**Region:**	exonic	**Exonic. func.:**	synonymous	**Gene ID:**	SH3KBP1
**RS number:**	rs201790502	**Clin. sign.:**	-	-	

Results

**Tool:**	**PredictSNP2**	**CADD**	**DANN**	**FATHMM**	**FunSeq2**	**GWAVA**
**Prediction:**	neutral	neutral	neutral	neutral	neutral	deleterious
**Score:**	-1.0000	13.8800	0.7217	0.1968	0.0000	0.6900
**Exp. accuracy:**	0.96	0.73	0.93	0.77	0.93	0.70

External links

[dbSNP] [GenBank] [HaploReg] [RegulomeDB] [UCSC] [Ensemble]

**Variant:** $X:19546045, G{\rightarrow}$T					
**Position:**	chrX:19546045	**Ref. allele:**	G	**Alt. allele:**	T
**Region:**	exonic	**Exonic. func.:**	synonymous	**Gene ID:**	SH3KBP1
**RS number:**	-	**Clin. sign.:**	-	-	

Results

**Tool:**	**PredictSNP2**	**CADD**	**DANN**	**FATHMM**	**FunSeq2**	**GWAVA**
**Prediction:**	neutral	neutral	neutral	deleterious	neutral	deleterious
**Score:**	-0.5886	6.3620	0.6651	0.8389	0.0000	0.7200
**Exp. accuracy:**	0.93	0.93	0.95	0.57	0.93	0.70

External links

[GenBank] [UCSC] [Ensemble]

**Variant:** $X : 19549980, G \rightarrow$A					
**Position:**	chrX:19549980	**Ref. allele:**	G	**Alt. allele:**	A
**Region:**	exonic	**Exonic. func.:**	synonymous	**Gene ID:**	SH3KBP1
**RS number:**	rs141233913	**Clin. sign.:**	-	-	

Results

**Tool:**	**PredictSNP2**	**CADD**	**DANN**	**FATHMM**	**FunSeq2**	**GWAVA**
**Prediction:**	neutral	neutral	neutral	deleterious	neutral	deleterious
**Score:**	-0.4714	10.3500	0.7728	0.9261	0.0000	0.6900
**Exp. accuracy:**	0.93	0.88	0.90	0.82	0.93	0.70

External links

[dbSNP] [GenBank] [HaploReg] [RegulomeDB] [UCSC] [Ensemble]

**Variant:** $X : 19549992, C \rightarrow$T					
**Position:**	chrX:19549992	**Ref. allele:**	C	**Alt. allele:**	T
**Region:**	exonic	**Exonic. func.:**	synonymous	**Gene ID:**	SH3KBP1
**RS number:**	-	**Clin. sign.:**	-	-	

Results

**Tool:**	**PredictSNP2**	**CADD**	**DANN**	**FATHMM**	**FunSeq2**	**GWAVA**
**Prediction:**	neutral	neutral	neutral	neutral	neutral	deleterious
**Score:**	-1.0000	14.7300	0.6358	0.0095	0.0000	0.5000
**Exp. accuracy:**	0.96	0.73	0.97	0.96	0.93	0.51

External links

[GenBank] [UCSC] [Ensemble]

**Variant:** $X : 19550007, T \rightarrow$C					
**Position:**	chrX:19550007	**Ref. allele:**	T	**Alt. allele:**	C
**Region:**	exonic	**Exonic. func.:**	synonymous	**Gene ID:**	SH3KBP1
**RS number:**	-	**Clin. sign.:**	-	-	

Results

**Tool:**	**PredictSNP2**	**CADD**	**DANN**	**FATHMM**	**FunSeq2**	**GWAVA**
**Prediction:**	neutral	neutral	neutral	neutral	neutral	deleterious
**Score:**	-1.0000	0.5080	0.6383	0.0454	0.0000	0.5500
**Exp. accuracy:**	0.96	0.95	0.97	0.96	0.93	0.66

External links

[GenBank] [UCSC] [Ensemble]

**Variant:** $X : 19550031, C \rightarrow$T					
**Position:**	chrX:19550031	**Ref. allele:**	C	**Alt. allele:**	T
**Region:**	exonic	**Exonic. func.:**	synonymous	**Gene ID:**	SH3KBP1
**RS number:**	rs372371711	**Clin. sign.:**	-	-	

Results

**Tool:**	**PredictSNP2**	**CADD**	**DANN**	**FATHMM**	**FunSeq2**	**GWAVA**
**Prediction:**	neutral	neutral	neutral	neutral	neutral	deleterious
**Score:**	-1.0000	14.8200	0.6260	0.1259	0.0000	0.5700
**Exp. accuracy:**	0.96	0.73	0.96	0.85	0.93	0.68

External links

[dbSNP] [GenBank] [RegulomeDB] [UCSC] [Ensemble]

**Variant:** $X : 19550034, A \rightarrow$G					
**Position:**	chrX:19550034	**Ref. allele:**	A	**Alt. allele:**	G
**Region:**	exonic	**Exonic. func.:**	synonymous	**Gene ID:**	SH3KBP1
**RS number:**	-	**Clin. sign.:**	-	-	

Results

**Tool:**	**PredictSNP2**	**CADD**	**DANN**	**FATHMM**	**FunSeq2**	**GWAVA**
**Prediction:**	neutral	neutral	neutral	neutral	neutral	deleterious
**Score:**	-1.0000	4.3410	0.5299	0.1750	0.0000	0.6700
**Exp. accuracy:**	0.96	0.94	0.97	0.80	0.93	0.70

External links

[GenBank] [UCSC] [Ensemble]

**Variant:** $X : 19550058, T \rightarrow$C					
**Position:**	chrX:19550058	**Ref. allele:**	T	**Alt. allele:**	C
**Region:**	exonic	**Exonic. func.:**	synonymous	**Gene ID:**	SH3KBP1
**RS number:**	rs35665839	**Clin. sign.:**	-	-	

Results

**Tool:**	**PredictSNP2**	**CADD**	**DANN**	**FATHMM**	**FunSeq2**	**GWAVA**
**Prediction:**	neutral	neutral	neutral	neutral	neutral	deleterious
**Score:**	-1.0000	3.6880	0.6719	0.0902	0.0000	0.5600
**Exp. accuracy:**	0.96	0.94	0.94	0.88	0.93	0.69

External links

[dbSNP] [GenBank] [HaploReg] [RegulomeDB] [UCSC] [Ensemble]

**Variant:** $X : 19550064, G \rightarrow$A					
**Position:**	chrX:19550064	**Ref. allele:**	G	**Alt. allele:**	A
**Region:**	exonic	**Exonic. func.:**	synonymous	**Gene ID:**	SH3KBP1
**RS number:**	rs139733939	**Clin. sign.:**	-	-	

Results

**Tool:**	**PredictSNP2**	**CADD**	**DANN**	**FATHMM**	**FunSeq2**	**GWAVA**
**Prediction:**	neutral	neutral	neutral	neutral	neutral	deleterious
**Score:**	-1.0000	14.6800	0.7157	0.4265	0.0000	0.5200
**Exp. accuracy:**	0.96	0.73	0.93	0.72	0.93	0.58

External links

[dbSNP] [GenBank] [HaploReg] [RegulomeDB] [UCSC] [Ensemble]

**Variant:** $X : 19550079, T \rightarrow$C					
**Position:**	chrX:19550079	**Ref. allele:**	T	**Alt. allele:**	C
**Region:**	exonic	**Exonic. func.:**	synonymous	**Gene ID:**	SH3KBP1
**RS number:**	-	**Clin. sign.:**	-	-	

Results

**Tool:**	**PredictSNP2**	**CADD**	**DANN**	**FATHMM**	**FunSeq2**	**GWAVA**
**Prediction:**	neutral	neutral	neutral	neutral	neutral	deleterious
**Score:**	-1.0000	5.3900	0.7964	0.7601	0.0000	0.7000
**Exp. accuracy:**	0.96	0.92	0.89	0.69	0.93	0.70

External links

[GenBank] [UCSC] [Ensemble]

**Variant:** $X : 19550081, A \rightarrow$G					
**Position:**	chrX:19550081	**Ref. allele:**	A	**Alt. allele:**	G
**Region:**	exonic	**Exonic. func.:**	synonymous	**Gene ID:**	SH3KBP1
**RS number:**	-	**Clin. sign.:**	-	-	

Results

**Tool:**	**PredictSNP2**	**CADD**	**DANN**	**FATHMM**	**FunSeq2**	**GWAVA**
**Prediction:**	neutral	neutral	neutral	neutral	neutral	deleterious
**Score:**	-1.0000	10.7000	0.7750	0.1567	0.0000	0.6000
**Exp. accuracy:**	0.96	0.88	0.90	0.83	0.93	0.64

External links

[GenBank] [UCSC] [Ensemble]

**Variant:** $X:19569161, G{\rightarrow}$T					
**Position:**	chrX:19569161	**Ref. allele:**	G	**Alt. allele:**	T
**Region:**	exonic	**Exonic. func.:**	synonymous	**Gene ID:**	SH3KBP1
**RS number:**	-	**Clin. sign.:**	-	-	

Results

**Tool:**	**PredictSNP2**	**CADD**	**DANN**	**FATHMM**	**FunSeq2**	**GWAVA**
**Prediction:**	neutral	neutral	neutral	neutral	neutral	deleterious
**Score:**	-1.0000	10.8600	0.6618	0.3728	0.0000	0.5100
**Exp. accuracy:**	0.96	0.88	0.95	0.72	0.93	0.55

External links

[GenBank] [UCSC] [Ensemble]

**Variant:** $X:19569185, A{\rightarrow}$G					
**Position:**	chrX:19569185	**Ref. allele:**	A	**Alt. allele:**	G
**Region:**	exonic	**Exonic. func.:**	synonymous	**Gene ID:**	SH3KBP1
**RS number:**	-	**Clin. sign.:**	-	-	

Results

**Tool:**	**PredictSNP2**	**CADD**	**DANN**	**FATHMM**	**FunSeq2**	**GWAVA**
**Prediction:**	neutral	neutral	neutral	deleterious	neutral	deleterious
**Score:**	-0.5809	7.3860	0.7111	0.8345	0.0000	0.6100
**Exp. accuracy:**	0.93	0.91	0.93	0.57	0.93	0.64

External links

[GenBank] [UCSC] [Ensemble]

**Variant:** $X:19588648, G{\rightarrow}$A					
**Position:**	chrX:19588648	**Ref. allele:**	G	**Alt. allele:**	A
**Region:**	exonic	**Exonic. func.:**	synonymous	**Gene ID:**	SH3KBP1
**RS number:**	-	**Clin. sign.:**	-	-	

Results

**Tool:**	**PredictSNP2**	**CADD**	**DANN**	**FATHMM**	**FunSeq2**	**GWAVA**
**Prediction:**	neutral	neutral	neutral	neutral	neutral	deleterious
**Score:**	-1.0000	0.2680	0.4824	0.0728	0.0000	0.5000
**Exp. accuracy:**	0.96	0.95	0.97	0.90	0.93	0.51

External links

[GenBank] [UCSC] [Ensemble]

**Variant:** $X : 19588657, C \to$T					
**Position:**	chrX:19588657	**Ref. allele:**	C	**Alt. allele:**	T
**Region:**	exonic	**Exonic. func.:**	synonymous	**Gene ID:**	SH3KBP1
**RS number:**	rs145308311	**Clin. sign.:**	-	-	

Results

**Tool:**	**PredictSNP2**	**CADD**	**DANN**	**FATHMM**	**FunSeq2**	**GWAVA**
**Prediction:**	neutral	neutral	neutral	neutral	neutral	deleterious
**Score:**	-1.0000	12.0400	0.7135	0.4606	0.0000	0.5400
**Exp. accuracy:**	0.96	0.83	0.93	0.72	0.93	0.62

External links

[dbSNP] [GenBank] [HaploReg] [RegulomeDB] [UCSC] [Ensemble]

**Variant:** $X : 19588666, C \to$A					
**Position:**	chrX:19588666	**Ref. allele:**	C	**Alt. allele:**	A
**Region:**	exonic	**Exonic. func.:**	synonymous	**Gene ID:**	SH3KBP1
**RS number:**	-	**Clin. sign.:**	-	-	

Results

**Tool:**	**PredictSNP2**	**CADD**	**DANN**	**FATHMM**	**FunSeq2**	**GWAVA**
**Prediction:**	neutral	deleterious	neutral	neutral	neutral	deleterious
**Score:**	-0.6107	15.9900	0.5847	0.6794	0.0000	0.5700
**Exp. accuracy:**	0.95	0.58	0.97	0.69	0.93	0.68

External links

[GenBank] [UCSC] [Ensemble]

**Variant:** $X : 19588666, C \to$T					
**Position:**	chrX:19588666	**Ref. allele:**	C	**Alt. allele:**	T
**Region:**	exonic	**Exonic. func.:**	synonymous	**Gene ID:**	SH3KBP1
**RS number:**	rs147648876	**Clin. sign.:**	-	-	

Results

**Tool:**	**PredictSNP2**	**CADD**	**DANN**	**FATHMM**	**FunSeq2**	**GWAVA**
**Prediction:**	neutral	neutral	neutral	neutral	neutral	deleterious
**Score:**	-1.0000	13.0000	0.6798	0.1920	0.0000	0.5700
**Exp. accuracy:**	0.96	0.77	0.94	0.80	0.93	0.68

External links

[dbSNP] [GenBank] [HaploReg] [RegulomeDB] [UCSC] [Ensemble]

**Variant:** $X : 19588672, C{\rightarrow}$T					
**Position:**	chrX:19588672	**Ref. allele:**	C	**Alt. allele:**	T
**Region:**	exonic	**Exonic. func.:**	synonymous	**Gene ID:**	SH3KBP1
**RS number:**	-	**Clin. sign.:**	-	-	

Results

**Tool:**	**PredictSNP2**	**CADD**	**DANN**	**FATHMM**	**FunSeq2**	**GWAVA**
**Prediction:**	neutral	neutral	neutral	neutral	neutral	deleterious
**Score:**	-1.0000	9.9380	0.3696	0.3341	0.0000	0.5700
**Exp. accuracy:**	0.96	0.91	0.97	0.73	0.93	0.68

External links

[GenBank] [UCSC] [Ensemble]

**Variant:** $X : 19588675, C{\rightarrow}$T					
**Position:**	chrX:19588675	**Ref. allele:**	C	**Alt. allele:**	T
**Region:**	exonic	**Exonic. func.:**	synonymous	**Gene ID:**	SH3KBP1
**RS number:**	rs2290805	**Clin. sign.:**	-	-	

Results

**Tool:**	**PredictSNP2**	**CADD**	**DANN**	**FATHMM**	**FunSeq2**	**GWAVA**
**Prediction:**	neutral	neutral	neutral	neutral	neutral	deleterious
**Score:**	-1.0000	8.3870	0.7240	0.0057	0.0000	0.5400
**Exp. accuracy:**	0.96	0.92	0.93	0.96	0.93	0.62

External links

[dbSNP] [GenBank] [HaploReg] [RegulomeDB] [UCSC] [Ensemble]

**Variant:** $X : 19588684, C{\rightarrow}$T					
**Position:**	chrX:19588684	**Ref. allele:**	C	**Alt. allele:**	T
**Region:**	exonic	**Exonic. func.:**	synonymous	**Gene ID:**	SH3KBP1
**RS number:**	rs199796248	**Clin. sign.:**	-	-	

Results

**Tool:**	**PredictSNP2**	**CADD**	**DANN**	**FATHMM**	**FunSeq2**	**GWAVA**
**Prediction:**	neutral	neutral	neutral	neutral	neutral	deleterious
**Score:**	-1.0000	11.5200	0.7542	0.0234	0.0000	0.5500
**Exp. accuracy:**	0.96	0.88	0.90	0.96	0.93	0.66

External links

[dbSNP] [GenBank] [HaploReg] [RegulomeDB] [UCSC] [Ensemble]

**Variant:** $X : 19588717, G \rightarrow$C					
**Position:**	chrX:19588717	**Ref. allele:**	G	**Alt. allele:**	C
**Region:**	exonic	**Exonic. func.:**	synonymous	**Gene ID:**	SH3KBP1
**RS number:**	-	**Clin. sign.:**	-	-	

Results

**Tool:**	**PredictSNP2**	**CADD**	**DANN**	**FATHMM**	**FunSeq2**	**GWAVA**
**Prediction:**	neutral	neutral	neutral	deleterious	neutral	deleterious
**Score:**	-0.5932	5.9920	0.6062	0.7935	0.0000	0.6400
**Exp. accuracy:**	0.93	0.93	0.96	0.57	0.93	0.68

External links

[GenBank] [UCSC] [Ensemble]

**Variant:** $X : 19588731, G \rightarrow$T					
**Position:**	chrX:19588731	**Ref. allele:**	G	**Alt. allele:**	T
**Region:**	exonic	**Exonic. func.:**	synonymous	**Gene ID:**	SH3KBP1
**RS number:**	-	**Clin. sign.:**	-	-	

Results

**Tool:**	**PredictSNP2**	**CADD**	**DANN**	**FATHMM**	**FunSeq2**	**GWAVA**
**Prediction:**	neutral	neutral	neutral	deleterious	neutral	deleterious
**Score:**	-0.4926	12.8800	0.6286	0.9216	0.0000	0.6700
**Exp. accuracy:**	0.93	0.77	0.97	0.78	0.93	0.70

External links

[GenBank] [UCSC] [Ensemble]

**Variant:** $X : 19588741, T \rightarrow$C					
**Position:**	chrX:19588741	**Ref. allele:**	T	**Alt. allele:**	C
**Region:**	exonic	**Exonic. func.:**	synonymous	**Gene ID:**	SH3KBP1
**RS number:**	-	**Clin. sign.:**	-	-	

Results

**Tool:**	**PredictSNP2**	**CADD**	**DANN**	**FATHMM**	**FunSeq2**	**GWAVA**
**Prediction:**	neutral	neutral	neutral	neutral	neutral	deleterious
**Score:**	-1.0000	3.3960	0.6280	0.4354	0.0000	0.5600
**Exp. accuracy:**	0.96	0.94	0.97	0.72	0.93	0.69

External links

[GenBank] [UCSC] [Ensemble]

**Variant:** $X : 19588741, T \rightarrow$G					
**Position:**	chrX:19588741	**Ref. allele:**	T	**Alt. allele:**	G
**Region:**	exonic	**Exonic. func.:**	synonymous	**Gene ID:**	SH3KBP1
**RS number:**	-	**Clin. sign.:**	-	-	

Results

**Tool:**	**PredictSNP2**	**CADD**	**DANN**	**FATHMM**	**FunSeq2**	**GWAVA**
**Prediction:**	neutral	neutral	neutral	neutral	neutral	deleterious
**Score:**	-1.0000	5.7100	0.6358	0.5045	0.0000	0.5600
**Exp. accuracy:**	0.96	0.92	0.97	0.70	0.93	0.69

External links

[GenBank] [UCSC] [Ensemble]

PredictSNP overview

Date: 2017/03/06
Genome version: GRCh38/hg38
PredictSNP version: 2.1

If you use PredictSNP and obtain scientific results that you publish, we would ask you to acknowledge the usage of PredictSNP by referencing the article below.

Bendl, J., Musil, M., Stourac, J., Zendulka, J., Damborsky, J., Brezovsky, J., 2016: PredictSNP2: A unified platform for accurately evaluating SNP effects by exploiting the different characteristics of variants in distinct genomic regions. *PLOS Computational Biology* 12: e1004962

	PredictSNP2		CADD		DANN		FATHMM		FunSeq2		GWAVA	
	Neu	Del	Neu	Del	Neu	Del	Neu	Del	Neu	Del	Neu	Del
Regulatory	0	0	0	0	0	0	0	0	0	0	0	0
Splicing	0	0	0	0	0	0	0	0	0	0	0	0
Missense	0	0	0	0	0	0	54	0	54	0	54	0
Synonymous	41	13	54	0	29	24	0	0	0	0	0	0
Nonsense	0	0	0	0	0	0	0	0	0	0	0	0
Others	0	0	0	0	0	0	0	0	0	0	0	0

**Variant:** $X: 19588744, C{\rightarrow}$A					
**Position:**	chrX:19588744	**Ref. allele:**	C	**Alt. allele:**	A
**Region:**	exonic	**Exonic. func.:**	synonymous	**Gene ID:**	SH3KBP1
**RS number:**	-	**Clin. sign.:**	-	-	

Results

**Tool:**	**PredictSNP2**	**CADD**	**DANN**	**FATHMM**	**FunSeq2**	**GWAVA**
**Prediction:**	neutral	neutral	neutral	neutral	neutral	deleterious
**Score:**	-1.0000	13.5600	0.6786	0.0281	0.0000	0.5500
**Exp. accuracy:**	0.96	0.73	0.94	0.96	0.93	0.66

External links

[GenBank] [UCSC] [Ensemble]

**Variant:** $X: 19588744, C{\rightarrow}$T					
**Position:**	chrX:19588744	**Ref. allele:**	C	**Alt. allele:**	T
**Region:**	exonic	**Exonic. func.:**	synonymous	**Gene ID:**	SH3KBP1
**RS number:**	-	**Clin. sign.:**	-	-	

Results

**Tool:**	**PredictSNP2**	**CADD**	**DANN**	**FATHMM**	**FunSeq2**	**GWAVA**
**Prediction:**	neutral	neutral	neutral	neutral	neutral	deleterious
**Score:**	-1.0000	12.8100	0.7393	0.0237	0.0000	0.5500
**Exp. accuracy:**	0.96	0.77	0.91	0.96	0.93	0.66

External links

[GenBank] [UCSC] [Ensemble]

**Variant:** $X: 19588756, A{\rightarrow}$G					
**Position:**	chrX:19588756	**Ref. allele:**	A	**Alt. allele:**	G
**Region:**	exonic	**Exonic. func.:**	synonymous	**Gene ID:**	SH3KBP1
**RS number:**	-	**Clin. sign.:**	-	-	

Results

**Tool:**	**PredictSNP2**	**CADD**	**DANN**	**FATHMM**	**FunSeq2**	**GWAVA**
**Prediction:**	neutral	neutral	neutral	neutral	neutral	deleterious
**Score:**	-1.0000	6.8640	0.6973	0.7487	0.0000	0.6700
**Exp. accuracy:**	0.96	0.92	0.94	0.69	0.93	0.70

External links

[GenBank] [UCSC] [Ensemble]

**Variant:** $X: 19588759, G{\rightarrow}A$					
**Position:**	chrX:19588759	**Ref. allele:**	G	**Alt. allele:**	A
**Region:**	exonic	**Exonic. func.:**	synonymous	**Gene ID:**	SH3KBP1
**RS number:**	rs145759622	**Clin. sign.:**	-	-	

Results

**Tool:**	**PredictSNP2**	**CADD**	**DANN**	**FATHMM**	**FunSeq2**	**GWAVA**
**Prediction:**	neutral	neutral	neutral	neutral	neutral	deleterious
**Score:**	-1.0000	12.8800	0.6534	0.0246	0.0000	0.6000
**Exp. accuracy:**	0.96	0.77	0.96	0.96	0.93	0.64

External links

[dbSNP] [GenBank] [HaploReg] [RegulomeDB] [UCSC] [Ensemble]

**Variant:** $X: 19588789, T{\rightarrow}C$					
**Position:**	chrX:19588789	**Ref. allele:**	T	**Alt. allele:**	C
**Region:**	exonic	**Exonic. func.:**	synonymous	**Gene ID:**	SH3KBP1
**RS number:**	-	**Clin. sign.:**	-	-	

Results

**Tool:**	**PredictSNP2**	**CADD**	**DANN**	**FATHMM**	**FunSeq2**	**GWAVA**
**Prediction:**	neutral	neutral	neutral	deleterious	neutral	deleterious
**Score:**	-0.5883	3.2800	0.6627	0.8493	0.0000	0.6600
**Exp. accuracy:**	0.93	0.94	0.95	0.57	0.93	0.70

External links

[GenBank] [UCSC] [Ensemble]

**Variant:** $X: 19592077, T{\rightarrow}C$					
**Position:**	chrX:19592077	**Ref. allele:**	T	**Alt. allele:**	C
**Region:**	exonic	**Exonic. func.:**	synonymous	**Gene ID:**	SH3KBP1
**RS number:**	rs369021832	**Clin. sign.:**	-	-	

Results

**Tool:**	**PredictSNP2**	**CADD**	**DANN**	**FATHMM**	**FunSeq2**	**GWAVA**
**Prediction:**	neutral	neutral	neutral	deleterious	neutral	deleterious
**Score:**	-0.4901	1.6180	0.7888	0.9178	0.0000	0.5800
**Exp. accuracy:**	0.93	0.95	0.89	0.78	0.93	0.65

External links

[dbSNP] [GenBank] [RegulomeDB] [UCSC] [Ensemble]

**Variant:** $X : 19592128, A \rightarrow$G					
**Position:**	chrX:19592128	**Ref. allele:**	A	**Alt. allele:**	G
**Region:**	exonic	**Exonic. func.:**	synonymous	**Gene ID:**	SH3KBP1
**RS number:**	-	**Clin. sign.:**	-	-	

Results

**Tool:**	**PredictSNP2**	**CADD**	**DANN**	**FATHMM**	**FunSeq2**	**GWAVA**
**Prediction:**	neutral	neutral	neutral	deleterious	neutral	deleterious
**Score:**	-0.4832	6.3070	0.6568	0.9261	0.0000	0.6400
**Exp. accuracy:**	0.93	0.93	0.95	0.82	0.93	0.68

External links

[GenBank] [UCSC] [Ensemble]

**Variant:** $X : 19592131, T \rightarrow$C					
**Position:**	chrX:19592131	**Ref. allele:**	T	**Alt. allele:**	C
**Region:**	exonic	**Exonic. func.:**	synonymous	**Gene ID:**	SH3KBP1
**RS number:**	rs372562490	**Clin. sign.:**	-	-	

Results

**Tool:**	**PredictSNP2**	**CADD**	**DANN**	**FATHMM**	**FunSeq2**	**GWAVA**
**Prediction:**	neutral	neutral	neutral	neutral	neutral	deleterious
**Score:**	-1.0000	1.2270	0.6183	0.6865	0.0000	0.6000
**Exp. accuracy:**	0.96	0.95	0.96	0.69	0.93	0.64

External links

[dbSNP] [GenBank] [RegulomeDB] [UCSC] [Ensemble]

**Variant:** $X : 19594965, G \rightarrow$A					
**Position:**	chrX:19594965	**Ref. allele:**	G	**Alt. allele:**	A
**Region:**	exonic	**Exonic. func.:**	synonymous	**Gene ID:**	SH3KBP1
**RS number:**	-	**Clin. sign.:**	-	-	

Results

**Tool:**	**PredictSNP2**	**CADD**	**DANN**	**FATHMM**	**FunSeq2**	**GWAVA**
**Prediction:**	neutral	neutral	neutral	deleterious	neutral	neutral
**Score:**	-0.5583	10.4200	0.7693	0.8699	0.0000	0.3200
**Exp. accuracy:**	0.93	0.88	0.90	0.60	0.93	0.53

External links

[GenBank] [UCSC] [Ensemble]

**Variant:** $X: 19594974, G{\rightarrow}$A					
**Position:**	chrX:19594974	**Ref. allele:**	G	**Alt. allele:**	A
**Region:**	exonic	**Exonic. func.:**	synonymous	**Gene ID:**	SH3KBP1
**RS number:**	-	**Clin. sign.:**	-	-	

Results

**Tool:**	**PredictSNP2**	**CADD**	**DANN**	**FATHMM**	**FunSeq2**	**GWAVA**
**Prediction:**	neutral	neutral	neutral	neutral	neutral	neutral
**Score:**	-1.0000	11.6200	0.6145	0.3520	0.0000	0.2900
**Exp. accuracy:**	0.96	0.88	0.96	0.73	0.93	0.56

External links

[GenBank] [UCSC] [Ensemble]

**Variant:** $X: 19594977, T{\rightarrow}$G					
**Position:**	chrX:19594977	**Ref. allele:**	T	**Alt. allele:**	G
**Region:**	exonic	**Exonic. func.:**	synonymous	**Gene ID:**	SH3KBP1
**RS number:**	rs143742226	**Clin. sign.:**	-	-	

Results

**Tool:**	**PredictSNP2**	**CADD**	**DANN**	**FATHMM**	**FunSeq2**	**GWAVA**
**Prediction:**	neutral	neutral	neutral	neutral	neutral	neutral
**Score:**	-1.0000	11.1400	0.7307	0.7865	0.0000	0.3300
**Exp. accuracy:**	0.96	0.88	0.91	0.69	0.93	0.56

External links

[dbSNP] [GenBank] [HaploReg] [RegulomeDB] [UCSC] [Ensemble]

**Variant:** $X: 19594995, G{\rightarrow}$A					
**Position:**	chrX:19594995	**Ref. allele:**	G	**Alt. allele:**	A
**Region:**	exonic	**Exonic. func.:**	synonymous	**Gene ID:**	SH3KBP1
**RS number:**	-	**Clin. sign.:**	-	-	

Results

**Tool:**	**PredictSNP2**	**CADD**	**DANN**	**FATHMM**	**FunSeq2**	**GWAVA**
**Prediction:**	neutral	neutral	neutral	neutral	neutral	neutral
**Score:**	-1.0000	7.9610	0.6322	0.7596	0.0000	0.3200
**Exp. accuracy:**	0.96	0.91	0.97	0.69	0.93	0.53

External links

[GenBank] [UCSC] [Ensemble]

**Variant:** $X : 19607959, C \rightarrow$T					
**Position:**	chrX:19607959	**Ref. allele:**	C	**Alt. allele:**	T
**Region:**	exonic	**Exonic. func.:**	synonymous	**Gene ID:**	SH3KBP1
**RS number:**	rs3747357	**Clin. sign.:**	-	-	

Results

**Tool:**	**PredictSNP2**	**CADD**	**DANN**	**FATHMM**	**FunSeq2**	**GWAVA**
**Prediction:**	neutral	neutral	neutral	neutral	neutral	neutral
**Score:**	-1.0000	10.3700	0.6051	0.0086	0.0000	0.3200
**Exp. accuracy:**	0.96	0.88	0.96	0.96	0.93	0.53

External links

[dbSNP] [GenBank] [HaploReg] [RegulomeDB] [UCSC] [Ensemble]

**Variant:** $X : 19607962, T \rightarrow$C					
**Position:**	chrX:19607962	**Ref. allele:**	T	**Alt. allele:**	C
**Region:**	exonic	**Exonic. func.:**	synonymous	**Gene ID:**	SH3KBP1
**RS number:**	-	**Clin. sign.:**	-	-	

Results

**Tool:**	**PredictSNP2**	**CADD**	**DANN**	**FATHMM**	**FunSeq2**	**GWAVA**
**Prediction:**	neutral	neutral	neutral	neutral	neutral	neutral
**Score:**	-1.0000	0.2600	0.3323	0.1528	0.0000	0.3400
**Exp. accuracy:**	0.96	0.95	0.97	0.83	0.93	0.56

External links

[GenBank] [UCSC] [Ensemble]

**Variant:** $X : 19607977, G \rightarrow$A					
**Position:**	chrX:19607977	**Ref. allele:**	G	**Alt. allele:**	A
**Region:**	exonic	**Exonic. func.:**	synonymous	**Gene ID:**	SH3KBP1
**RS number:**	-	**Clin. sign.:**	-	-	

Results

**Tool:**	**PredictSNP2**	**CADD**	**DANN**	**FATHMM**	**FunSeq2**	**GWAVA**
**Prediction:**	neutral	neutral	neutral	neutral	neutral	neutral
**Score:**	-1.0000	11.6600	0.6895	0.4237	0.0000	0.3100
**Exp. accuracy:**	0.96	0.83	0.94	0.72	0.93	0.53

External links

[GenBank] [UCSC] [Ensemble]

**Variant:** $X : 19607986, G{\rightarrow}$A					
**Position:**	chrX:19607986	**Ref. allele:**	G	**Alt. allele:**	A
**Region:**	exonic	**Exonic. func.:**	synonymous	**Gene ID:**	SH3KBP1
**RS number:**	rs199869591	**Clin. sign.:**	-	-	

Results

**Tool:**	**PredictSNP2**	**CADD**	**DANN**	**FATHMM**	**FunSeq2**	**GWAVA**
**Prediction:**	neutral	neutral	neutral	neutral	neutral	neutral
**Score:**	-1.0000	4.0450	0.7125	0.0635	0.0000	0.3100
**Exp. accuracy:**	0.96	0.94	0.93	0.92	0.93	0.53

External links

[dbSNP] [GenBank] [HaploReg] [RegulomeDB] [UCSC] [Ensemble]

**Variant:** $X : 19608013, C{\rightarrow}$T					
**Position:**	chrX:19608013	**Ref. allele:**	C	**Alt. allele:**	T
**Region:**	exonic	**Exonic. func.:**	synonymous	**Gene ID:**	SH3KBP1
**RS number:**	-	**Clin. sign.:**	-	-	

Results

**Tool:**	**PredictSNP2**	**CADD**	**DANN**	**FATHMM**	**FunSeq2**	**GWAVA**
**Prediction:**	neutral	neutral	neutral	neutral	neutral	neutral
**Score:**	-1.0000	12.3300	0.6140	0.5219	0.0000	0.3400
**Exp. accuracy:**	0.96	0.83	0.96	0.70	0.93	0.56

External links

[GenBank] [UCSC] [Ensemble]

**Variant:** $X : 19608034, G{\rightarrow}$A					
**Position:**	chrX:19608034	**Ref. allele:**	G	**Alt. allele:**	A
**Region:**	exonic	**Exonic. func.:**	synonymous	**Gene ID:**	SH3KBP1
**RS number:**	rs372715805	**Clin. sign.:**	-	-	

Results

**Tool:**	**PredictSNP2**	**CADD**	**DANN**	**FATHMM**	**FunSeq2**	**GWAVA**
**Prediction:**	neutral	neutral	neutral	neutral	neutral	neutral
**Score:**	-1.0000	9.2320	0.6240	0.0122	0.0000	0.3200
**Exp. accuracy:**	0.96	0.91	0.96	0.96	0.93	0.53

External links

[dbSNP] [GenBank] [RegulomeDB] [UCSC] [Ensemble]

Variant: $X : 19608037, G \rightarrow$A					
**Position:**	chrX:19608037	**Ref. allele:**	G	**Alt. allele:**	A
**Region:**	exonic	**Exonic. func.:**	synonymous	**Gene ID:**	SH3KBP1
**RS number:**	-	**Clin. sign.:**	-	-	

Results

**Tool:**	**PredictSNP2**	**CADD**	**DANN**	**FATHMM**	**FunSeq2**	**GWAVA**
**Prediction:**	neutral	neutral	neutral	neutral	neutral	neutral
**Score:**	-1.0000	11.8700	0.8148	0.2766	0.0000	0.2900
**Exp. accuracy:**	0.96	0.83	0.90	0.73	0.93	0.56

External links

[GenBank] [UCSC] [Ensemble]

Variant: $X : 19608037, G \rightarrow$T					
**Position:**	chrX:19608037	**Ref. allele:**	G	**Alt. allele:**	T
**Region:**	exonic	**Exonic. func.:**	synonymous	**Gene ID:**	SH3KBP1
**RS number:**	-	**Clin. sign.:**	-	-	

Results

**Tool:**	**PredictSNP2**	**CADD**	**DANN**	**FATHMM**	**FunSeq2**	**GWAVA**
**Prediction:**	neutral	neutral	neutral	neutral	neutral	neutral
**Score:**	-1.0000	9.5720	0.7712	0.5145	0.0000	0.2900
**Exp. accuracy:**	0.96	0.91	0.90	0.70	0.93	0.56

External links

[GenBank] [UCSC] [Ensemble]

Variant: $X : 19631879, G \rightarrow$A					
**Position:**	chrX:19631879	**Ref. allele:**	G	**Alt. allele:**	A
**Region:**	exonic	**Exonic. func.:**	synonymous	**Gene ID:**	SH3KBP1
**RS number:**	-	**Clin. sign.:**	-	-	

Results

**Tool:**	**PredictSNP2**	**CADD**	**DANN**	**FATHMM**	**FunSeq2**	**GWAVA**
**Prediction:**	neutral	neutral	neutral	neutral	neutral	neutral
**Score:**	-1.0000	9.7570	0.7735	0.6253	0.0000	0.3300
**Exp. accuracy:**	0.96	0.91	0.90	0.69	0.93	0.56

External links

[GenBank] [UCSC] [Ensemble]

**Variant:** $X : 19631957, G \rightarrow$A					
**Position:**	chrX:19631957	**Ref. allele:**	G	**Alt. allele:**	A
**Region:**	exonic	**Exonic. func.:**	synonymous	**Gene ID:**	SH3KBP1
**RS number:**	-	**Clin. sign.:**	-	-	

Results

**Tool:**	**PredictSNP2**	**CADD**	**DANN**	**FATHMM**	**FunSeq2**	**GWAVA**
**Prediction:**	neutral	neutral	neutral	deleterious	neutral	?
**Score:**	-0.5688	15.0600	0.7694	0.7976	0.0000	0.4800
**Exp. accuracy:**	0.93	0.73	0.90	0.57	0.93	0.43

External links

[GenBank] [UCSC] [Ensemble]

**Variant:** $X : 19683832, C \rightarrow$T					
**Position:**	chrX:19683832	**Ref. allele:**	C	**Alt. allele:**	T
**Region:**	exonic	**Exonic. func.:**	synonymous	**Gene ID:**	SH3KBP1
**RS number:**	rs111744926	**Clin. sign.:**	-	-	

Results

**Tool:**	**PredictSNP2**	**CADD**	**DANN**	**FATHMM**	**FunSeq2**	**GWAVA**
**Prediction:**	neutral	neutral	neutral	neutral	neutral	neutral
**Score:**	-1.0000	9.4010	0.2521	0.0049	0.0000	0.3100
**Exp. accuracy:**	0.96	0.91	0.97	0.96	0.93	0.53

External links

[dbSNP] [GenBank] [HaploReg] [RegulomeDB] [UCSC] [Ensemble]

**Variant:** $X : 19683880, T \rightarrow$C					
**Position:**	chrX:19683880	**Ref. allele:**	T	**Alt. allele:**	C
**Region:**	exonic	**Exonic. func.:**	synonymous	**Gene ID:**	SH3KBP1
**RS number:**	-	**Clin. sign.:**	-	-	

Results

**Tool:**	**PredictSNP2**	**CADD**	**DANN**	**FATHMM**	**FunSeq2**	**GWAVA**
**Prediction:**	neutral	neutral	neutral	neutral	neutral	neutral
**Score:**	-1.0000	5.3740	0.7302	0.6944	0.0000	0.3000
**Exp. accuracy:**	0.96	0.92	0.91	0.69	0.93	0.57

External links

[GenBank] [UCSC] [Ensemble]

**Variant:** $X: 19683892, G \to$A					
**Position:**	chrX:19683892	**Ref. allele:**	G	**Alt. allele:**	A
**Region:**	exonic	**Exonic. func.:**	synonymous	**Gene ID:**	SH3KBP1
**RS number:**	rs375218887	**Clin. sign.:**	-	-	

Results

**Tool:**	**PredictSNP2**	**CADD**	**DANN**	**FATHMM**	**FunSeq2**	**GWAVA**
**Prediction:**	neutral	neutral	neutral	deleterious	neutral	neutral
**Score:**	-0.4858	10.5400	0.6290	0.9262	0.0000	0.4600
**Exp. accuracy:**	0.93	0.88	0.97	0.82	0.93	0.54

External links

[dbSNP] [GenBank] [RegulomeDB] [UCSC] [Ensemble]

**Variant:** $X: 19683916, C \to$T					
**Position:**	chrX:19683916	**Ref. allele:**	C	**Alt. allele:**	T
**Region:**	exonic	**Exonic. func.:**	synonymous	**Gene ID:**	SH3KBP1
**RS number:**	-	**Clin. sign.:**	-	-	

Results

**Tool:**	**PredictSNP2**	**CADD**	**DANN**	**FATHMM**	**FunSeq2**	**GWAVA**
**Prediction:**	neutral	neutral	neutral	neutral	neutral	neutral
**Score:**	-1.0000	12.4300	0.5659	0.5673	0.0000	0.3000
**Exp. accuracy:**	0.96	0.79	0.97	0.70	0.93	0.57

External links

[GenBank] [UCSC] [Ensemble]

**Variant:** $X: 19683952, T \to$C					
**Position:**	chrX:19683952	**Ref. allele:**	T	**Alt. allele:**	C
**Region:**	exonic	**Exonic. func.:**	synonymous	**Gene ID:**	SH3KBP1
**RS number:**	-	**Clin. sign.:**	-	-	

Results

**Tool:**	**PredictSNP2**	**CADD**	**DANN**	**FATHMM**	**FunSeq2**	**GWAVA**
**Prediction:**	neutral	neutral	neutral	neutral	neutral	neutral
**Score:**	-1.0000	0.4100	0.5620	0.0367	0.0000	0.2600
**Exp. accuracy:**	0.96	0.95	0.97	0.96	0.93	0.54

External links

[GenBank] [UCSC] [Ensemble]

**Variant:** $X:19683958, G{\to}$A					
**Position:**	chrX:19683958	**Ref. allele:**	G	**Alt. allele:**	A
**Region:**	exonic	**Exonic. func.:**	synonymous	**Gene ID:**	SH3KBP1
**RS number:**	-	**Clin. sign.:**	-	-	

Results

**Tool:**	**PredictSNP2**	**CADD**	**DANN**	**FATHMM**	**FunSeq2**	**GWAVA**
**Prediction:**	neutral	neutral	neutral	neutral	neutral	neutral
**Score:**	-1.0000	8.2490	0.6158	0.3322	0.0000	0.3400
**Exp. accuracy:**	0.96	0.92	0.96	0.73	0.93	0.56

External links

[GenBank] [UCSC] [Ensemble]

**Variant:** $X:19684006, G{\to}$A					
**Position:**	chrX:19684006	**Ref. allele:**	G	**Alt. allele:**	A
**Region:**	exonic	**Exonic. func.:**	synonymous	**Gene ID:**	SH3KBP1
**RS number:**	rs373105646	**Clin. sign.:**	-	-	

Results

**Tool:**	**PredictSNP2**	**CADD**	**DANN**	**FATHMM**	**FunSeq2**	**GWAVA**
**Prediction:**	neutral	neutral	neutral	neutral	neutral	neutral
**Score:**	-1.0000	11.1000	0.7645	0.0585	0.0000	0.4100
**Exp. accuracy:**	0.96	0.88	0.90	0.92	0.93	0.56

External links

[dbSNP] [GenBank] [RegulomeDB] [UCSC] [Ensemble]

**Variant:** $X:19695622, T{\to}$C					
**Position:**	chrX:19695622	**Ref. allele:**	T	**Alt. allele:**	C
**Region:**	exonic	**Exonic. func.:**	synonymous	**Gene ID:**	SH3KBP1
**RS number:**	-	**Clin. sign.:**	-	-	

Results

**Tool:**	**PredictSNP2**	**CADD**	**DANN**	**FATHMM**	**FunSeq2**	**GWAVA**
**Prediction:**	neutral	neutral	neutral	neutral	neutral	deleterious
**Score:**	-1.0000	1.0740	0.5636	0.1528	0.0000	0.5100
**Exp. accuracy:**	0.96	0.95	0.97	0.83	0.93	0.55

External links

[GenBank] [UCSC] [Ensemble]

**Variant:** $X : 19695622, T{\rightarrow}$G					
**Position:**	chrX:19695622	**Ref. allele:**	T	**Alt. allele:**	G
**Region:**	exonic	**Exonic. func.:**	synonymous	**Gene ID:**	SH3KBP1
**RS number:**	-	**Clin. sign.:**	-	-	

Results

**Tool:**	**PredictSNP2**	**CADD**	**DANN**	**FATHMM**	**FunSeq2**	**GWAVA**
**Prediction:**	neutral	neutral	neutral	neutral	neutral	deleterious
**Score:**	-1.0000	5.6730	0.5976	0.0587	0.0000	0.5100
**Exp. accuracy:**	0.96	0.92	0.96	0.92	0.93	0.55

External links

[GenBank] [UCSC] [Ensemble]

**Variant:** $X : 19695655, C{\rightarrow}$T					
**Position:**	chrX:19695655	**Ref. allele:**	C	**Alt. allele:**	T
**Region:**	exonic	**Exonic. func.:**	synonymous	**Gene ID:**	SH3KBP1
**RS number:**	rs145372202	**Clin. sign.:**	-	-	

Results

**Tool:**	**PredictSNP2**	**CADD**	**DANN**	**FATHMM**	**FunSeq2**	**GWAVA**
**Prediction:**	neutral	neutral	neutral	neutral	neutral	deleterious
**Score:**	-1.0000	12.3200	0.6134	0.0236	0.0000	0.5800
**Exp. accuracy:**	0.96	0.83	0.96	0.96	0.93	0.65

External links

[dbSNP] [GenBank] [HaploReg] [RegulomeDB] [UCSC] [Ensemble]

**Variant:** $X : 19695682, G{\rightarrow}$A					
**Position:**	chrX:19695682	**Ref. allele:**	G	**Alt. allele:**	A
**Region:**	exonic	**Exonic. func.:**	synonymous	**Gene ID:**	SH3KBP1
**RS number:**	-	**Clin. sign.:**	-	-	

Results

**Tool:**	**PredictSNP2**	**CADD**	**DANN**	**FATHMM**	**FunSeq2**	**GWAVA**
**Prediction:**	neutral	neutral	neutral	deleterious	neutral	deleterious
**Score:**	-0.5883	9.9210	0.6962	0.8588	0.0000	0.6700
**Exp. accuracy:**	0.93	0.91	0.94	0.57	0.93	0.70

External links

[GenBank] [UCSC] [Ensemble]

**Variant:** $X:19695706, C\rightarrow$T					
**Position:**	chrX:19695706	**Ref. allele:**	C	**Alt. allele:**	T
**Region:**	exonic	**Exonic. func.:**	synonymous	**Gene ID:**	SH3KBP1
**RS number:**	-	**Clin. sign.:**	-	-	

Results

**Tool:**	**PredictSNP2**	**CADD**	**DANN**	**FATHMM**	**FunSeq2**	**GWAVA**
**Prediction:**	neutral	neutral	neutral	neutral	neutral	deleterious
**Score:**	-1.0000	14.6900	0.6257	0.4316	0.0000	0.5800
**Exp. accuracy:**	0.96	0.73	0.96	0.72	0.93	0.65

External links

[GenBank] [UCSC] [Ensemble]

**Variant:** $X:19695709, G\rightarrow$A					
**Position:**	chrX:19695709	**Ref. allele:**	G	**Alt. allele:**	A
**Region:**	exonic	**Exonic. func.:**	synonymous	**Gene ID:**	SH3KBP1
**RS number:**	rs184591130	**Clin. sign.:**	-	-	

Results

**Tool:**	**PredictSNP2**	**CADD**	**DANN**	**FATHMM**	**FunSeq2**	**GWAVA**
**Prediction:**	neutral	neutral	neutral	neutral	neutral	deleterious
**Score:**	-1.0000	10.0500	0.6394	0.2472	0.0000	0.5800
**Exp. accuracy:**	0.96	0.91	0.97	0.73	0.93	0.65

External links

[dbSNP] [GenBank] [HaploReg] [RegulomeDB] [UCSC] [Ensemble]

**Variant:** $X:19695721, T\rightarrow$C					
**Position:**	chrX:19695721	**Ref. allele:**	T	**Alt. allele:**	C
**Region:**	exonic	**Exonic. func.:**	synonymous	**Gene ID:**	SH3KBP1
**RS number:**	-	**Clin. sign.:**	-	-	

Results

**Tool:**	**PredictSNP2**	**CADD**	**DANN**	**FATHMM**	**FunSeq2**	**GWAVA**
**Prediction:**	neutral	neutral	neutral	deleterious	neutral	deleterious
**Score:**	-0.4678	3.1140	0.7356	0.9472	0.0000	0.7700
**Exp. accuracy:**	0.93	0.94	0.91	0.91	0.93	0.70

External links

[GenBank] [UCSC] [Ensemble]

**Variant:** $X:19706890, C{\rightarrow}$T					
**Position:**	chrX:19706890	**Ref. allele:**	C	**Alt. allele:**	T
**Region:**	exonic	**Exonic. func.:**	synonymous	**Gene ID:**	SH3KBP1
**RS number:**	-	**Clin. sign.:**	-	-	

Results

**Tool:**	**PredictSNP2**	**CADD**	**DANN**	**FATHMM**	**FunSeq2**	**GWAVA**
**Prediction:**	neutral	neutral	neutral	neutral	neutral	neutral
**Score:**	-1.0000	11.7100	0.8190	0.5002	0.0000	0.3200
**Exp. accuracy:**	0.96	0.83	0.90	0.70	0.93	0.53

External links

[GenBank] [UCSC] [Ensemble]

**Variant:** $X:19706905, G{\rightarrow}$A					
**Position:**	chrX:19706905	**Ref. allele:**	G	**Alt. allele:**	A
**Region:**	exonic	**Exonic. func.:**	synonymous	**Gene ID:**	SH3KBP1
**RS number:**	-	**Clin. sign.:**	-	-	

Results

**Tool:**	**PredictSNP2**	**CADD**	**DANN**	**FATHMM**	**FunSeq2**	**GWAVA**
**Prediction:**	neutral	neutral	neutral	neutral	neutral	neutral
**Score:**	-1.0000	10.9800	0.8352	0.0096	0.0000	0.4300
**Exp. accuracy:**	0.96	0.88	0.90	0.96	0.93	0.54

External links

[GenBank] [UCSC] [Ensemble]

**Variant:** $X:19706929, G{\rightarrow}$A					
**Position:**	chrX:19706929	**Ref. allele:**	G	**Alt. allele:**	A
**Region:**	exonic	**Exonic. func.:**	synonymous	**Gene ID:**	SH3KBP1
**RS number:**	rs368141192	**Clin. sign.:**	-	-	

Results

**Tool:**	**PredictSNP2**	**CADD**	**DANN**	**FATHMM**	**FunSeq2**	**GWAVA**
**Prediction:**	neutral	neutral	neutral	neutral	neutral	neutral
**Score:**	-1.0000	6.1360	0.7478	0.0135	0.0000	0.4200
**Exp. accuracy:**	0.96	0.93	0.91	0.96	0.93	0.54

External links

[dbSNP] [GenBank] [RegulomeDB] [UCSC] [Ensemble]

**Variant:** $X : 19706944, G \to A$					
**Position:**	chrX:19706944	**Ref. allele:**	G	**Alt. allele:**	A
**Region:**	exonic	**Exonic. func.:**	synonymous	**Gene ID:**	SH3KBP1
**RS number:**	rs371576734	**Clin. sign.:**	-	-	

Results

**Tool:**	**PredictSNP2**	**CADD**	**DANN**	**FATHMM**	**FunSeq2**	**GWAVA**
**Prediction:**	neutral	neutral	neutral	neutral	neutral	deleterious
**Score:**	-1.0000	9.5030	0.5860	0.7846	0.0000	0.5100
**Exp. accuracy:**	0.96	0.91	0.97	0.69	0.93	0.55

External links

[dbSNP] [GenBank] [RegulomeDB] [UCSC] [Ensemble]

**Variant:** $X : 19706983, G \to A$					
**Position:**	chrX:19706983	**Ref. allele:**	G	**Alt. allele:**	A
**Region:**	exonic	**Exonic. func.:**	synonymous	**Gene ID:**	SH3KBP1
**RS number:**	-	**Clin. sign.:**	-	-	

Results

**Tool:**	**PredictSNP2**	**CADD**	**DANN**	**FATHMM**	**FunSeq2**	**GWAVA**
**Prediction:**	neutral	neutral	neutral	neutral	neutral	neutral
**Score:**	-1.0000	7.3650	0.8381	0.0111	0.0000	0.3700
**Exp. accuracy:**	0.96	0.91	0.90	0.96	0.93	0.56

External links

[GenBank] [UCSC] [Ensemble]

**Variant:** $X : 19746328, G \to A$					
**Position:**	chrX:19746328	**Ref. allele:**	G	**Alt. allele:**	A
**Region:**	exonic	**Exonic. func.:**	synonymous	**Gene ID:**	SH3KBP1
**RS number:**	-	**Clin. sign.:**	-	-	

Results

**Tool:**	**PredictSNP2**	**CADD**	**DANN**	**FATHMM**	**FunSeq2**	**GWAVA**
**Prediction:**	neutral	neutral	neutral	neutral	neutral	neutral
**Score:**	-1.0000	9.4230	0.6730	0.7326	0.0000	0.3600
**Exp. accuracy:**	0.96	0.91	0.94	0.69	0.93	0.55

External links

[GenBank] [UCSC] [Ensemble]

**Variant:** $X : 19746340, C \to$T					
**Position:**	chrX:19746340	**Ref. allele:**	C	**Alt. allele:**	T
**Region:**	exonic	**Exonic. func.:**	synonymous	**Gene ID:**	SH3KBP1
**RS number:**	rs375522869	**Clin. sign.:**	-	-	

Results

**Tool:**	**PredictSNP2**	**CADD**	**DANN**	**FATHMM**	**FunSeq2**	**GWAVA**
**Prediction:**	neutral	neutral	neutral	neutral	neutral	neutral
**Score:**	-1.0000	14.1800	0.7016	0.0201	0.0000	0.3600
**Exp. accuracy:**	0.96	0.73	0.94	0.96	0.93	0.55

External links

[dbSNP] [GenBank] [RegulomeDB] [UCSC] [Ensemble]

**Variant:** $X : 19746376, T \to$C					
**Position:**	chrX:19746376	**Ref. allele:**	T	**Alt. allele:**	C
**Region:**	exonic	**Exonic. func.:**	synonymous	**Gene ID:**	SH3KBP1
**RS number:**	-	**Clin. sign.:**	-	-	

Results

**Tool:**	**PredictSNP2**	**CADD**	**DANN**	**FATHMM**	**FunSeq2**	**GWAVA**
**Prediction:**	neutral	neutral	neutral	neutral	neutral	neutral
**Score:**	-1.0000	1.5860	0.5733	0.4735	0.0000	0.3300
**Exp. accuracy:**	0.96	0.95	0.97	0.72	0.93	0.56

External links

[GenBank] [UCSC] [Ensemble]

**Variant:** $X : 19746379, G \to$A					
**Position:**	chrX:19746379	**Ref. allele:**	G	**Alt. allele:**	A
**Region:**	exonic	**Exonic. func.:**	synonymous	**Gene ID:**	SH3KBP1
**RS number:**	-	**Clin. sign.:**	-	-	

Results

**Tool:**	**PredictSNP2**	**CADD**	**DANN**	**FATHMM**	**FunSeq2**	**GWAVA**
**Prediction:**	neutral	neutral	neutral	neutral	neutral	neutral
**Score:**	-1.0000	1.9240	0.7287	0.0212	0.0000	0.3600
**Exp. accuracy:**	0.96	0.95	0.91	0.96	0.93	0.55

External links

[GenBank] [UCSC] [Ensemble]

**Variant:** $X : 19746397, A \to$G					
**Position:**	chrX:19746397	**Ref. allele:**	A	**Alt. allele:**	G
**Region:**	exonic	**Exonic. func.:**	synonymous	**Gene ID:**	SH3KBP1
**RS number:**	-	**Clin. sign.:**	-	-	

Results

**Tool:**	**PredictSNP2**	**CADD**	**DANN**	**FATHMM**	**FunSeq2**	**GWAVA**
**Prediction:**	neutral	neutral	neutral	neutral	neutral	neutral
**Score:**	-1.0000	11.1800	0.7860	0.7385	0.0000	0.4600
**Exp. accuracy:**	0.96	0.88	0.90	0.69	0.93	0.54

External links

[GenBank] [UCSC] [Ensemble]

**Variant:** $X : 19836149, A \to$C					
**Position:**	chrX:19836149	**Ref. allele:**	A	**Alt. allele:**	C
**Region:**	exonic	**Exonic. func.:**	synonymous	**Gene ID:**	SH3KBP1
**RS number:**	-	**Clin. sign.:**	-	-	

Results

**Tool:**	**PredictSNP2**	**CADD**	**DANN**	**FATHMM**	**FunSeq2**	**GWAVA**
**Prediction:**	neutral	neutral	neutral	deleterious	neutral	deleterious
**Score:**	-0.5585	0.2660	0.7690	0.8673	0.0000	0.6700
**Exp. accuracy:**	0.93	0.95	0.90	0.60	0.93	0.70

External links

[GenBank] [UCSC] [Ensemble]

**Variant:** $X : 19836152, T \to$C					
**Position:**	chrX:19836152	**Ref. allele:**	T	**Alt. allele:**	C
**Region:**	exonic	**Exonic. func.:**	synonymous	**Gene ID:**	SH3KBP1
**RS number:**	-	**Clin. sign.:**	-	-	

Results

**Tool:**	**PredictSNP2**	**CADD**	**DANN**	**FATHMM**	**FunSeq2**	**GWAVA**
**Prediction:**	neutral	neutral	neutral	deleterious	neutral	deleterious
**Score:**	-0.5662	3.2830	0.6961	0.8654	0.0000	0.6700
**Exp. accuracy:**	0.93	0.94	0.94	0.60	0.93	0.70

External links

[GenBank] [UCSC] [Ensemble]

**Variant:** $X : 19836161, G \rightarrow$A					
**Position:**	chrX:19836161	**Ref. allele:**	G	**Alt. allele:**	A
**Region:**	exonic	**Exonic. func.:**	synonymous	**Gene ID:**	SH3KBP1
**RS number:**	-	**Clin. sign.:**	-	-	

Results

**Tool:**	**PredictSNP2**	**CADD**	**DANN**	**FATHMM**	**FunSeq2**	**GWAVA**
**Prediction:**	neutral	neutral	neutral	neutral	neutral	deleterious
**Score:**	-1.0000	9.3650	0.6393	0.6683	0.0000	0.5400
**Exp. accuracy:**	0.96	0.91	0.97	0.69	0.93	0.62

External links

[GenBank] [UCSC] [Ensemble]

**Variant:** $X : 19836200, G \rightarrow$A					
**Position:**	chrX:19836200	**Ref. allele:**	G	**Alt. allele:**	A
**Region:**	exonic	**Exonic. func.:**	synonymous	**Gene ID:**	SH3KBP1
**RS number:**	rs35356139	**Clin. sign.:**	-	-	

Results

**Tool:**	**PredictSNP2**	**CADD**	**DANN**	**FATHMM**	**FunSeq2**	**GWAVA**
**Prediction:**	neutral	neutral	neutral	deleterious	neutral	deleterious
**Score:**	-0.4850	9.8720	0.8514	0.9205	0.0000	0.6300
**Exp. accuracy:**	0.93	0.91	0.90	0.78	0.93	0.66

External links

[dbSNP] [GenBank] [HaploReg] [RegulomeDB] [UCSC] [Ensemble]

**Variant:** $X : 19836224, G \rightarrow$A					
**Position:**	chrX:19836224	**Ref. allele:**	G	**Alt. allele:**	A
**Region:**	exonic	**Exonic. func.:**	synonymous	**Gene ID:**	SH3KBP1
**RS number:**	-	**Clin. sign.:**	-	-	

Results

**Tool:**	**PredictSNP2**	**CADD**	**DANN**	**FATHMM**	**FunSeq2**	**GWAVA**
**Prediction:**	neutral	neutral	neutral	deleterious	neutral	deleterious
**Score:**	-0.5746	13.6100	0.7128	0.7964	0.0000	0.6400
**Exp. accuracy:**	0.93	0.73	0.93	0.57	0.93	0.68

External links

[GenBank] [UCSC] [Ensemble]

Variant: $X : 19836230, C{\rightarrow}$G					
**Position:**	chrX:19836230	**Ref. allele:**	C	**Alt. allele:**	G
**Region:**	exonic	**Exonic. func.:**	synonymous	**Gene ID:**	SH3KBP1
**RS number:**	-	**Clin. sign.:**	-	-	

Results

**Tool:**	**PredictSNP2**	**CADD**	**DANN**	**FATHMM**	**FunSeq2**	**GWAVA**
**Prediction:**	neutral	neutral	neutral	neutral	neutral	deleterious
**Score:**	-1.0000	8.6900	0.7045	0.0284	0.0000	0.6200
**Exp. accuracy:**	0.96	0.92	0.94	0.96	0.93	0.66

External links

[GenBank] [UCSC] [Ensemble]

Variant: $X : 19836245, G{\rightarrow}$A					
**Position:**	chrX:19836245	**Ref. allele:**	G	**Alt. allele:**	A
**Region:**	exonic	**Exonic. func.:**	synonymous	**Gene ID:**	SH3KBP1
**RS number:**	rs7882276	**Clin. sign.:**	-	-	

Results

**Tool:**	**PredictSNP2**	**CADD**	**DANN**	**FATHMM**	**FunSeq2**	**GWAVA**
**Prediction:**	neutral	neutral	neutral	neutral	neutral	deleterious
**Score:**	-1.0000	9.6710	0.6257	0.7752	0.0000	0.6800
**Exp. accuracy:**	0.96	0.91	0.96	0.69	0.93	0.70

External links

[dbSNP] [GenBank] [HaploReg] [RegulomeDB] [UCSC] [Ensemble]

Variant: $X : 19836272, T{\rightarrow}$A					
**Position:**	chrX:19836272	**Ref. allele:**	T	**Alt. allele:**	A
**Region:**	exonic	**Exonic. func.:**	synonymous	**Gene ID:**	SH3KBP1
**RS number:**	-	**Clin. sign.:**	-	-	

Results

**Tool:**	**PredictSNP2**	**CADD**	**DANN**	**FATHMM**	**FunSeq2**	**GWAVA**
**Prediction:**	neutral	neutral	neutral	deleterious	neutral	deleterious
**Score:**	-0.5316	12.4600	0.7807	0.8754	0.0000	0.7200
**Exp. accuracy:**	0.93	0.79	0.90	0.64	0.93	0.70

External links

[GenBank] [UCSC] [Ensemble]
