# Supplementary material for: Computational analysis of functional SNPs in Alzheimer’s disease-associated endocytosis genes
Source: PeerJ. 2019 Sep 30;7:e7667. doi: 10.7717/peerj.7667 (PMC6776068; doi:10.7717/peerj.7667)
Supplement: Table S9 [file peerj-07-7667-s013.docx]

**Supplemental Table S9. The physical and chemical properties of deleterious nsSNPs of**

***PICALM*, *SYNJ1* and *SH3KBP1* proteins.**

| **Variant ID** | **AA Subs.** | **Hydropathy**** | **Polarity** | **Charges** |
| --- | --- | --- | --- | --- |
| ***PICALM*** |  |  |  |  |
| rs750147583 | L106S | - to + | Nonpolar to polar | Neutral to neutral |
| rs780443419 | F109S | - to + | Nonpolar to polar | Neutral to neutral |
| rs145115354 | D144N | + to + | Polar to polar | Negative to neutral |
| rs765338634 | L179P | - to - | Nonpolar to nonpolar | Neutral to neutral |
| ***SYNJ1*** |  |  |  |  |
| rs781675993 | N200K | + to + | Polar to polar | Neutral to positive |
| rs398122403 | R258Q | + to + | Polar to polar | Positive to neutral |
| rs762909719 | R289Q | + to + | Polar to polar | Positive to neutral |
| rs771755243 | V338A | - to - | Nonpolar to nonpolar | Neutral to neutral |
| rs768897710 | Q414R | + to + | Polar to polar | Neutral to positive |
| rs779479360 | G437D | - to + | Zwitterion to polar | Neutral to negative |
| rs775515863 | G487R | - to + | Zwitterion to polar | Neutral to positive |
| rs752563697 | G494D | - to + | Zwitterion to polar | Neutral to negative |
| rs771070426 | I515T | - to + | Nonpolar to polar | Neutral to neutral |
| rs756845805 | G627S | - to + | Zwitterion to polar | Neutral to neutral |
| rs751110096 | C723G | * to - | Polar to zwitterion | Neutral to neutral |
| rs147929290 | I746T | - to + | Nonpolar to polar | Neutral to neutral |
| rs745418083 | L776P | - to - | Nonpolar to nonpolar | Neutral to neutral |
| ***SH3KBP1*** |  |  |  |  |
| rs770229859 | R648Q | + to + | Polar to polar | Positive to neutral |
| **Representation of hydropathy: - = hydrophobic; + = hydrophilic; * = moderate | | | | |
