# Supplementary material for: Computational analysis of functional SNPs in Alzheimer’s disease-associated endocytosis genes
Source: PeerJ. 2019 Sep 30;7:e7667. doi: 10.7717/peerj.7667 (PMC6776068; doi:10.7717/peerj.7667)
Supplement: Table S10 [file peerj-07-7667-s014.docx]

**Supplemental Table S10. Prediction of pathogenic and non-pathogenic**

***PSEN1* nsSNPs.**

| **Variants ID** | **AA Subs** | **SIFT score** | **PolyPhen PSIC score** | **Mutation Assessor** | **I-Mutant DDG** | **SNPs&GO** |
| --- | --- | --- | --- | --- | --- | --- |
| **Pathogenic nsSNPs** | |  | | | | |
| rs63750599 | L85P | 0 | 1 | Medium | -1.79 | 0.797 |
| rs63750004 | I143T | 0 | 0.999 | Medium | -1.99 | 0.621 |
| rs63751010 | W165G | 0 | 0.999 | High | -2.1 | 0.813 |
| rs63750577 | S170F | 0 | 0.983 | Medium | -1.12 | 0.608 |
| rs1555355289 | I238M | 0 | 0.966 | Medium | -1.73 | 0.515 |
| rs63750301 | P264L | 0 | 1 | High | -1.35 | 0.8 |
| rs63750231 | E280A | 0 | 0.998 | Medium | -0.88 | 0.257 |
| rs63750298 | T291P | 0.02 | 0.983 | Medium | -1.07 | 0.578 |
| rs63750687 | L381V | 0 | 0.993 | High | -1.82 | 0.607 |
| rs63750646 | G384A | 0.01 | 0.999 | High | -1.5 | 0.829 |
| **Non-pathogenic nsSNPs** | |  | | | | |
| rs63750592 | R35Q | 0.57 | 0 | Low | -1.61 | 0.036 |
| rs63750771 | F175S | 0.09 | 0.928 | Low | -1.12 | 0.492 |
| rs112451138 | V191A | 0.07 | 0.422 | Medium | -3.05 | 0.232 |
| rs777545298 | A305S | 0.56 | 0.056 | Neutral | -1.18 | 0.032 |
| rs141822345 | A370G | 0.39 | 0 | Low | -1.25 | 0.02 |
