# Supplementary material for: Computational analysis of functional SNPs in Alzheimer’s disease-associated endocytosis genes
Source: PeerJ. 2019 Sep 30;7:e7667. doi: 10.7717/peerj.7667 (PMC6776068; doi:10.7717/peerj.7667)
Supplement: Table S11 [file peerj-07-7667-s015.docx]

**Supplemental Table S11. Deleterious variants that were identified in the**

**NGS studies of AD.**

| **Chr** | **Position** | | **Gene** | **Variants ID** | **Consequences** | **Study** |
| --- | --- | --- | --- | --- | --- | --- |
|  | **GRCh37** | **GRCh38** |  |  |  |  |
| 11 | 85687667 | 85976624 | PICALM | rs768239913 | Splice site | ADSP (WES) |
| 11 | 85779799 | 86068757 | PICALM | rs367839126 | sSNP | ADSP (WES) |
| 21 | 34003354 | 32631044 | SYNJ1 | rs111516740 | 3’UTR | ADSP (WES) |
| 21 | 34017324 | 32645014 | SYNJ1 | rs533064963 | Splice site | ADSP (WES) |
| 21 | 34038278 | 32665968 | SYNJ1 | rs147929290 | nsSNP | ADSP (WES), ADNI |
| X | 19553160 | 19535042 | SH3KBP1 | rs192424738 | 3’UTR | ADNI |
| X | 19564042 | 19545924 | SH3KBP1 | rs61761898 | Splice site | ADNI, MSBB |
